# Supplementary material for: Rewiring of gene expression in circulating white blood cells is associated with pregnancy outcome in heifers (Bos taurus)
Source: Sci Rep. 2020 Oct 8;10:16786. doi: 10.1038/s41598-020-73694-w (PMC7544915; doi:10.1038/s41598-020-73694-w)
Supplement: Supplementary file 14 — Supplementary information. [file 41598_2020_73694_MOESM14_ESM.pdf]

">

Rewiring of gene expression in circulating white blood cells is associated with pregnancy outcome in heifers (Bos taurus)

Sarah E. Moorey, Bailey N. Walker, Michelle F. Elmore, Joshua B. Elmore, Soren P. Rodning and Fernando H. Biase

June 2020

-
-
- Script to count the features according to Ensembl annotation
  - mRNA
  - small RNA
- Script to create the matrix with the counts for all samples
  - mRNA
  - small RNA
- Data Overview
  - Figures 1b,c
- Differential mRNA:mRNA coexpression associated with pregnancy outcome
  - Supplementary Table 1, data used for Figure 1a
  - Supplementary Table 2
  - Supplementary Fig. 1a,b
  - Figures 1b,c
  - Supplementary Table 4
  - Figure 1d
  - Supplementary Table 5
- Differential miRNA:mRNA coexpression associated with pregnancy outcome
  - Supplementary table 6
  - Supplementary Fig. 2
  - Supplementary Table 7
  - Figure 3a
  - Figure 3b
  - Supplementary Fig. 3
  - Supplementary Table 8
  - Figure 3c
  - Supplementary Table 9
  - Supplementary Table 10
- Differential gene expression associated with pregnancy outcome
  - AI Pregnant vs non pregnant
    - Results from edgeR
    - Results from edgeR both years
    - Results from DESeq
    - Results from DESeq from both years
  - Supplementary Fig. 4
  - Supplementary table 11
- DEG Analysis for mRNA
  - AI Pregnant vs Pregnant to Natural Service
    - Results from edgeR
    - Results from DESeq
- DEG Analysis for mRNA
  - Pregnant to Natural Service vs non pregnant
    - Results from edgeR
    - Results from DESeq
  - Supplementary table 12
  - Figure 4a
  - Figure 4b
  - Figure 4c
- DEG Analysis for miRNA
  - Pregnant to Artificial Insemination vs non pregnant
    - Results from edgeR
    - Results from DESeq
  - AI Pregnant vs Pregnant to Natural Service
    - Results from edgeR
    - Results from DESeq
  - Pregnant to Natural Service vs non pregnant

        - Results from edgeR
        - Results from DESeq
- Assessment of transcript levels as predictors of pregnancy outcome
    - Supplementary Fig. 5
    - Figure 5d
    - Figure 5e
    - Figure 5f

This file was created to permit reproducibility of the findings described in the paper. Please direct questions to Fernando Biase: ***fbiase*** at ***vt.edu***
The raw data is deposited on GEO repository under the following access GSE146041 (https://www.ncbi.nlm.nih.gov/geo/query/acc.cgi?acc=GSE146041). For the reproduction of this code please download the R binary file containing all the objects corresponding to the data used in this work: resource_data_2019_06_28.RData (https://github.com/Biase-Lab/heifer_transcriptome/blob/master/resources/resource_data_2019_06_28.RData)

```
library("DESeq2", lib.loc="/usr/lib/R/site-library")
library('edgeR', lib.loc="/usr/lib/R/site-library")
library("statmod", lib.loc="/usr/lib/R/site-library")
library("ggplot2", lib.loc="/usr/lib/R/site-library")
library("ggrepel", lib.loc="/usr/lib/R/site-library")
library("biomaRt", lib.loc="/usr/lib/R/site-library")
library("goseq", lib.loc="/usr/lib/R/site-library")
library("WGCNA", lib.loc="/usr/lib/R/site-library")
library ("reshape2", lib.loc="/usr/lib/R/site-library")
library ("bigmemory", lib.loc="/usr/lib/R/site-library")
library("doParallel", lib.loc="/usr/lib/R/site-library")
library("gtools", lib.loc="/usr/lib/R/site-library")
library('org.Bt.eg.db', lib.loc="/usr/lib/R/site-library")
library('DT', lib.loc="/usr/lib/R/site-library")
library("Rtsne", lib.loc="/usr/lib/R/site-library")
library("ggpubr", lib.loc="/usr/lib/R/site-library")
library('grid', lib.loc="/usr/lib/R/site-library")
library('gridExtra', lib.loc="/usr/lib/R/site-library")
library("flashClust", lib.loc="/usr/lib/R/site-library")
library("parallelDist", lib.loc="/usr/lib/R/site-library")
library("ComplexHeatmap", lib.loc="/usr/lib/R/site-library")
library("circlize", lib.loc="/usr/lib/R/site-library")
library("caret", lib.loc="/usr/lib/R/site-library")
library("caretEnsemble", lib.loc="/usr/lib/R/site-library")
library("foreach", lib.loc="/usr/lib/R/site-library")
library("TestCor", lib.loc="/usr/lib/R/site-library")
library("scales", lib.loc="/usr/lib/R/site-library")
library("stringi", lib.loc="/usr/lib/R/site-library")
library("networkD3" , lib.loc="/usr/lib/R/site-library")

#source("multiplot.R") #from Cookbook for R Multiple graphs on one page
#(http://www.cookbook-r.com/Graphs/Multiple_graphs_on_one_page_%28ggplot2%29/)
```

Script to count the features according to Ensembl annotation

mRNA

Code used to produce the counts for mRNA data for each animal. Note that this was executed outside R as a bash command on the terminal.

```
#!/bin/bash
for folder in /data/auburn/heifer_pregnancy/proj_2018/align_genome/SL*; do
#input files
gff_file=/home/fernando/genomes/Bos_taurus.ARS-UCD1.2.95.gtf
alignment=$folder/Aligned.out.merged.filtered.sorted.bam
#output files
sample=$(echo $folder | awk '{n=split($0,a,"/");print a[7]}')
output=/data/auburn/heifer_pregnancy/proj_2018/count_mRNA/$sample.count
/home/fernando/bioinfo/subread-1.6.2-Linux-x86_64/bin/featureCounts -s2 -a $gff_file -o $output -F 'GTF' -t 'exo
n' -g 'gene_id' --ignoreDup -p -T 10  $alignment
done;
```

small RNA

Code used to produce the counts for small RNA data for each animal. Note that this was executed outside R as a bash command on the terminal.

```
#!/bin/bash
for folder in /data/auburn/heifer_pregnancy/proj_2018/alignment_small_rna_a/heifer*; do
#input files
gff_file=/home/fernando/genomes/Bos_taurus.ARS-UCD1.2.95.gtf
alignment=$folder/aligned.sorted.bam
#output files
sample=$(echo $folder | awk '{n=split($0,a,"/");print a[7]}')
output=/data/auburn/heifer_pregnancy/proj_2018/count_small_rna_ensembl/$sample.count
/home/fernando/bioinfo/subread-1.6.2-Linux-x86_64/bin/featureCounts -a $gff_file -o $output -F 'GTF' -t 'exon' -g
'gene_id' --ignoreDup  -T 10  $alignment
done;
```

Script to create the matrix with the counts for all samples

mRNA

Code used to produce the matrix containing all the counts for mRNA data.

```
setwd("/data/auburn/heifer_pregnancy/proj_2018/count_mRNA")
files<-list.files(".", recursive=T, pattern=".count" )
files<-files[grep("summary", files, invert = TRUE)]
files<-files[grep("featurecount", files, invert = TRUE)]
#create the count data
count<-data.frame()
count_pwbc<-data.frame(matrix(nrow=27570))
for (n in 1:length(files)) {
  count<-read.delim(files[n], header =TRUE, sep = "\t",  stringsAsFactors = FALSE,comment.char = "#")
  count<-count[,c(1,7)]
  count_pwbc<-cbind(count_pwbc,count)
}

dim(count_pwbc) #27570
rownames(count_pwbc)<-count_pwbc[,2]
count_pwbc<-count_pwbc[,c(3,5,7,9,11,13,15,17,19,21,23,25,27,29,31,33,35,37)]
colnames(count_pwbc)<-substr(colnames(count_pwbc), 55,62)
dim(count_pwbc) #27570
head(count_pwbc)

#write.table(count_pwbc,file="/home/fernando/auburn/heifer_pregnancy/proj_2018/analysis/resources/2019_05_02_unfi
ltered_count_pwbc.txt", sep = "\t",append = FALSE, quote = FALSE, row.names = TRUE)
```

Code used to produce the matrix containing all the counts for mRNA data produced in 2017. Original data can be obtained from GSE103628 (https://www.ncbi.nlm.nih.gov/geo/query/acc.cgi?acc=GSE103628)

```
setwd("/data/auburn/heifer_pregnancy/proj_2018/count_mRNA_proj2017")
files<-list.files(".", recursive=T, pattern=".count" )
files<-files[grep("summary", files, invert = TRUE)]
files<-files[grep("featurecount", files, invert = TRUE)]
#create the count data
count<-data.frame()
count_pwbc_2017<-data.frame(matrix(nrow=27570))
for (n in 1:length(files)) {
  count<-read.delim(files[n], header =TRUE, sep = "\t",  stringsAsFactors = FALSE,comment.char = "#")
  count<-count[,c(1,7)]
  count_pwbc_2017<-cbind(count_pwbc_2017,count)
}

dim(count_pwbc_2017) #27570
rownames(count_pwbc_2017)<-count_pwbc_2017[,2]
count_pwbc_2017<-count_pwbc_2017[,c(3,5,7,9,11,13,15,17,19,21,23,25,27,29,31,33,35,37,39,41,43,45,47,49)]
colnames(count_pwbc_2017)<-substr(colnames(count_pwbc_2017),74,81)
dim(count_pwbc_2017) #27570
head(count_pwbc_2017)

#write.table(count_pwbc_2017,file="/home/fernando/auburn/heifer_pregnancy/proj_2018/analysis/resources/2019_05_02_unfiltered_count_pwbc_2017.txt", sep = "\t",append = FALSE, quote = FALSE, row.names = TRUE)
```


```
setwd("/data/auburn/heifer_pregnancy/proj_2018/count_mRNA_proj2017")
files<-list.files(".", recursive=T, pattern=".count" )
files<-files[grep("summary", files, invert = TRUE)]
files<-files[grep("featurecount", files, invert = TRUE)]
#create the count data
count<-data.frame()
count_pwbc_2017<-data.frame(matrix(nrow=27570))
for (n in 1:length(files)) {
  count<-read.delim(files[n], header =TRUE, sep = "\t",  stringsAsFactors = FALSE,comment.char = "#")
  count<-count[,c(1,7)]
  count_pwbc_2017<-cbind(count_pwbc_2017,count)
}

dim(count_pwbc_2017) #27570
rownames(count_pwbc_2017)<-count_pwbc_2017[,2]
count_pwbc_2017<-count_pwbc_2017[,c(3,5,7,9,11,13,15,17,19,21,23,25,27,29,31,33,35,37,39,41,43,45,47,49)]
colnames(count_pwbc_2017)<-substr(colnames(count_pwbc_2017),74,81)
dim(count_pwbc_2017) #27570
head(count_pwbc_2017)

#write.table(count_pwbc_2017,file="/home/fernando/auburn/heifer_pregnancy/proj_2018/analysis/resources/2019_05_02_unfiltered_count_pwbc_2017.txt", sep = "\t",append = FALSE, quote = FALSE, row.names = TRUE)
```


small RNA

Code used to produce the matrix containing all the counts for miRNA data.

```
setwd("/data/auburn/heifer_pregnancy/proj_2018/count_small_rna_ensembl")
files<-list.files(".", recursive=T, pattern=".count" )
files<-files[grep("summary", files, invert = TRUE)]
files<-files[grep("featurecount", files, invert = TRUE)]
#create the count data
count<-data.frame()
count_miRNA_plasma<-data.frame(matrix(nrow=27570))
for (n in 1:length(files)) {
  count<-read.delim(files[n], header =TRUE, sep = "\t",  stringsAsFactors = FALSE,comment.char = "#")
  count<-count[,c(1,7)]
  count_miRNA_plasma<-cbind(count_miRNA_plasma,count)
}

dim(count_miRNA_plasma) #27570
rownames(count_miRNA_plasma)<-count_miRNA_plasma[,2]
count_miRNA_plasma<-count_miRNA_plasma[,c(3,5,7,9,11,13,15,17,19,21,23,25,27,29,31,33,35,37)]
colnames(count_miRNA_plasma)<-substr(colnames(count_miRNA_plasma), 64,73)
dim(count_miRNA_plasma) #27570
head(count_miRNA_plasma)

#write.table(count_miRNA_plasma,file="/home/fernando/auburn/heifer_pregnancy/proj_2018/analysis/resources/2019_05_02_unfiltered_count_miRNA_plasma.txt", sep = "\t",append = FALSE, quote = FALSE, row.names = TRUE)
```

Code to generate objects with annotation. Executed and the files saved on February 05 2019.

```
cow<-useMart("ensembl", dataset = "btaurus_gene_ensembl")
annotation.ensembl.symbol<-getBM(attributes = c('ensembl_gene_id', 'external_gene_name','description','hgnc_symbo
l','gene_biotype','transcript_length'),  values = "*", mart = cow)
annotation.ensembl.symbol<-getBM(attributes = c('ensembl_gene_id','external_gene_name','description','hgnc_symbo
l','gene_biotype','transcript_length'),  values = "*", mart = cow)
annotation.ensembl.symbol<-annotation.ensembl.symbol[order(annotation.ensembl.symbol$ensembl_gene_id, -annotatio
n.ensembl.symbol$transcript_length),]
annotation.ensembl.symbol<-annotation.ensembl.symbol[!duplicated(annotation.ensembl.symbol$ensembl_gene_id),]
annotation.GO.biomart<-getBM(attributes = c('ensembl_gene_id', 'external_gene_name','go_id','name_1006','namespac
e_1003'),  values = "*", mart = cow)
```

Set the working directories according to your path structure.

```
rm(list=ls())
sourcefile_path<-"/mnt/storage/auburn/heifer_pregnancy/proj_2018/analysis/resources"
result_path<-"/mnt/storage/auburn/heifer_pregnancy/proj_2018/analysis/results"
```

Create the .RData with all the data needed

```
count_miRNA_plasma<-read.delim(paste(sourcefile_path, "2019_05_02_unfiltered_count_miRNA_plasma.txt.bz2", sep="/"
), stringsAsFactors=FALSE)
count_pwbc<-read.delim(paste(sourcefile_path, "2019_05_02_unfiltered_count_pwbc.txt.bz2", sep="/"), stringsAsFact
ors=FALSE)
count_pwbc_2017<-read.delim(paste(sourcefile_path, "2019_05_02_unfiltered_count_pwbc_2017.txt.bz2", sep="/"), str
ingsAsFactors=FALSE)

gene.length<-read.delim(paste(sourcefile_path, "gene_length.txt.bz2", sep="/"), stringsAsFactors=FALSE)
annotation.ensembl.symbol<-read.delim(paste(sourcefile_path, "2019_02_05_anontation_cow.txt.bz2", sep="/"), strin
gsAsFactors=FALSE)
annotation.GO.biomart<-read.delim(paste(sourcefile_path, "2019_02_05_anontation_GO_cow.txt.bz2", sep="/"), string
sAsFactors=FALSE)

bta_miRWalk_CDS<-read.delim(paste(sourcefile_path, "bta_miRWalk_CDS.txt.bz2", sep="/"), stringsAsFactors=FALSE)
bta_miRWalk_3UTR<-read.delim(paste(sourcefile_path, "bta_miRWalk_3UTR.txt.bz2", sep="/"), stringsAsFactors=FALSE)
bta_miRWalk_5UTR<-read.delim(paste(sourcefile_path, "bta_miRWalk_5UTR.txt.bz2", sep="/"), stringsAsFactors=FALSE)
gc()
#save(count_miRNA_plasma, count_pwbc, count_pwbc_2017, gene.length, annotation.ensembl.symbol, annotation.GO.biom
art,bta_miRWalk_CDS,bta_miRWalk_3UTR,bta_miRWalk_5UTR, file=paste(sourcefile_path, "resource_data_2019_06_28.RDat
a", sep="/"), compress="bzip2")
```

Load the files needed for analyses.

```
load( file=paste(sourcefile_path,"resource_data_2019_06_28.RData", sep="/"), verbose =TRUE)
```

```
## Loading objects:
##   count_miRNA_plasma
##   count_pwbc
##   count_pwbc_2017
##   gene.length
##   annotation.ensembl.symbol
##   annotation.GO.biomart
##   bta_miRWalk_CDS
##   bta_miRWalk_3UTR
##   bta_miRWalk_5UTR
```

```
source(paste(sourcefile_path,"multiplot.R", sep="/"))
```

Filtering of the mRNA dataset.

```
#Filter to remove the library with extremely low read counts
count_pwbc<-count_pwbc[,-3]
#Filter to remove lowly expressed genes
keep<-rowSums( cpm(count_pwbc) >= 2 ) >= 5
count_pwbc<-count_pwbc[keep,]

#Filter to retain only protein coding genes
count_pwbc<-merge(count_pwbc,annotation.ensembl.symbol, by.x="row.names", by.y="ensembl_gene_id", all=FALSE)
count_pwbc<-count_pwbc[ which(count_pwbc$gene_biotype=='protein_coding'), ]
rownames(count_pwbc)<-count_pwbc$Row.names
count_pwbc<-count_pwbc[,c(10,4,16,18,8,5,9,15,11,2,6,12,3,13,14,7,17)]
colnames(count_pwbc) <- c("AI_92", "AI_99", "AI_114", "AI_117", "AI_119", "AI_120", "NB_93", "NB_103", "NB_108",
"NB_111", "NB_116", "NB_121", "NP_94", "NP_101", "NP_107", "NP_113", "NP_115")
```

```
#Filter 2017 data to remove lowly expressed genes
count_pwbc_2017<-count_pwbc_2017[,c(13,14,15,16,17,18,19,20,21,22,23,24)]
keep<-rowSums( cpm(count_pwbc_2017) >= 2 ) >= 6
count_pwbc_2017<-count_pwbc_2017[keep,]
dim(count_pwbc_2017) #10602
```

```
## [1] 10602    12
```

```
#Filter 2017 data to retain only protein coding genes
count_pwbc_2017<-merge(count_pwbc_2017,annotation.ensembl.symbol, by.x="row.names", by.y="ensembl_gene_id", all=F
ALSE)
count_pwbc_2017<-count_pwbc_2017[ which(count_pwbc_2017$gene_biotype=='protein_coding'), ]
rownames(count_pwbc_2017)<-count_pwbc_2017[,1]
count_pwbc_2017<-count_pwbc_2017[,c(2:13)]
colnames(count_pwbc_2017) <- c("AI_63", "NP_59", "NP_61", "NP_53", "AI_62", "AI_49", "AI_42", "AI_55", "NP_39",
"AI_36","NP_64", "NP_58")
```

```
merged_datasets<-merge(count_pwbc,count_pwbc_2017, by="row.names", all=FALSE)
rownames(merged_datasets)<-merged_datasets[,1]
merged_datasets<-merged_datasets[,2:30]
```

Calculate counts per million reads and fragments per kilobase per million reads.

```
gene_length<-subset(gene.length, Geneid %in% rownames(count_pwbc))
gene_length<-gene_length[order(gene_length$Geneid),]
count_pwbc<-count_pwbc[order(rownames(count_pwbc)),]
table(rownames(count_pwbc)==gene_length$Geneid) #all True
```

```
##
##  TRUE
## 10496
```

```
cpm_pwbc<-cpm(count_pwbc,normalized.lib.sizes = TRUE,log = FALSE)
rpkm_pwbc<-rpkm(count_pwbc, gene.length = gene_length$Length, normalized.lib.sizes = TRUE,log = FALSE)

rpkm_AIpreg<-rpkm_pwbc[,c(1,2,3,4,5,6)]
#dim(rpkm_AIpreg) #10496 X 6
#head(rpkm_AIpreg)
rpkm_NBpreg<-rpkm_pwbc[,c(7,8,9,10,11,12)]
#dim(rpkm_NBpreg) #10496 X 6
#head(rpkm_NBpreg)
rpkm_NOTpreg<-rpkm_pwbc[,c(13,14,15,16,17)]
#dim(rpkm_NOTpreg) #10496 X 5
#head(rpkm_NOTpreg)

cpm_AIpreg<-cpm_pwbc[,c(1,2,3,4,5,6)]
cpm_NBpreg<-cpm_pwbc[,c(7,8,9,10,11,12)]
cpm_NOTpreg<-cpm_pwbc[,c(13,14,15,16,17)]
```

```
#for merged dataset
gene_length_merged<-subset(gene.length, Geneid %in% rownames(merged_datasets))
gene_length_merged<-gene_length_merged[order(gene_length_merged$Geneid),]
merged_datasets<-merged_datasets[order(rownames(merged_datasets)),]
table(rownames(merged_datasets)==gene_length_merged$Geneid) #all True
```

```
##
## TRUE
## 9960
```

```
cpm_merged_datasets<-cpm(merged_datasets,normalized.lib.sizes = TRUE,log = FALSE)
rpkm_merged_datasets<-rpkm(merged_datasets, gene.length = gene_length_merged$Length, normalized.lib.sizes = TRUE,
log = FALSE)

rpkm_AIpreg_merged<-rpkm_merged_datasets[,c(1,2,3,4,5,6,18,22,23,24,25,27)]
#dim(rpkm_AIpreg_merged) #9960 X 6
#head(rpkm_AIpreg_merged)

rpkm_NOTpreg_merged<-rpkm_merged_datasets[,c(13,14,15,16,17,19,20,21,26,28,29)]
#dim(rpkm_NOTpreg_merged) #9960 X 5
#head(rpkm_NOTpreg_merged)
```

Filtering of the miRNA dataset.

```
#Filter to remove the library with extremely low read counts
count_miRNA_plasma<-count_miRNA_plasma[,-17]
#Filter to remove data with low read counts
keep<-rowSums( cpm(count_miRNA_plasma) > 1 ) >= 5
count_miRNA_plasma<-count_miRNA_plasma[keep,]
dim(count_miRNA_plasma) #1086
```

```
## [1] 1086   17
```

```
#Fiter to retain only microRNAs
count_miRNA_plasma<-merge(count_miRNA_plasma,annotation.ensembl.symbol, by.x="row.names", by.y="ensembl_gene_id",
all=FALSE)
count_miRNA_plasma<-count_miRNA_plasma[ which(count_miRNA_plasma$gene_biotype=='miRNA'), ]
rownames(count_miRNA_plasma)<-count_miRNA_plasma$Row.names
count_miRNA_plasma<-count_miRNA_plasma[,c(15,18,8,11,12,13,16,3,5,6,10,14,17,2,4,7,9)]
names(count_miRNA_plasma) <- c("AI_92", "AI_99", "AI_114", "AI_117", "AI_119", "AI_120", "NB_93", "NB_103", "NB_1
08", "NB_111", "NB_116", "NB_121", "NP_94", "NP_101", "NP_107", "NP_113", "NP_115")
```

Calculate counts per million reads and fragments per kilobase per million reads.

```
gene_length_miRNA<-subset(gene.length, Geneid %in% rownames(count_miRNA_plasma))
gene_length_miRNA<-gene_length_miRNA[order(gene_length_miRNA$Geneid),]
count_miRNA_plasma<-count_miRNA_plasma[order(rownames(count_miRNA_plasma)),]
table(rownames(count_miRNA_plasma)==gene_length_miRNA$Geneid) #all TRUE
```

```
##
## TRUE
##  290
```

```
cpm_miRNA<-cpm(count_miRNA_plasma,normalized.lib.sizes = TRUE,log = FALSE)
rpkm_miRNA<-rpkm(count_miRNA_plasma, gene.length = gene_length_miRNA$Length, normalized.lib.sizes = TRUE,log = FA
LSE)

#write.table(cpm_miRNA,file="/mnt/storage/auburn/heifer_pregnancy/proj_2018/analysis/results/2020_02_17_filtered_
count_miRNA_plasma.txt", sep = "\t",append = FALSE, quote = FALSE, row.names = TRUE)

rpkm_AIpreg_miRNA<-rpkm_miRNA[,c(1,2,3,4,5,6)]
rpkm_NBpreg_miRNA<-rpkm_miRNA[,c(7,8,9,10,11,12)]
rpkm_NOTpreg_miRNA<-rpkm_miRNA[,c(13,14,15,16,17)]

cpm_AIpreg_miRNA<-cpm_miRNA[,c(1,2,3,4,5,6)]
cpm_NBpreg_miRNA<-cpm_miRNA[,c(7,8,9,10,11,12)]
cpm_NOTpreg_miRNA<-cpm_miRNA[,c(13,14,15,16,17)]
```

Data Overview

Figures 1b,c

```
data.for.tsne.mRNA<-t(log2(rpkm_pwbc+1))
data.for.tsne.miRNA<-t(log2(rpkm_miRNA+1))
set.seed(9434)
tsne_mRNA <- Rtsne(data.for.tsne.mRNA, dims = 2, theta = 0, perplexity=2, verbose=FALSE, max_iter = 500000)
tsne_miRNA <- Rtsne(data.for.tsne.miRNA, dims = 2, theta = 0, perplexity=2, verbose=FALSE, max_iter = 500000)
```

```
group<-factor(c("Preg_AI","Preg_AI","Preg_AI","Preg_AI","Preg_AI","Preg_AI","Preg_NB","Preg_NB","Preg_NB","Preg_N
B","Preg_NB","Preg_NB","Not_Preg","Not_Preg","Not_Preg","Not_Preg","Not_Preg"), levels=c("Preg_AI","Preg_NB", "No
t_Preg"))

TSNE_mRNA<-data.frame("DIM1"=tsne_mRNA$Y[,1], "DIM2"=tsne_mRNA$Y[,2], "group"=group )
TSNE_miRNA<-data.frame("DIM1"=tsne_miRNA$Y[,1], "DIM2"=tsne_miRNA$Y[,2], "group"=group )

plot1<-ggplot(data=TSNE_mRNA, aes(x=DIM1,y=DIM2) ) +
  geom_point(aes(colour = factor(group)), size=3 , alpha =0.8 ) +
  scale_color_manual(values= c("#0072B2", "#000000","#D55E00"), labels=c("AI-preg","NB-preg", "non preg") ,name
 =NULL) +
  scale_y_continuous(name="t-SNE dim 2")+
  scale_x_continuous(name="t-SNE dim 1")+
  ggtitle("10496 protein coding genes")+
  theme_bw(base_size = 11)+
  theme(aspect.ratio =1,
        axis.text=element_blank(),
        legend.position = "none",
        plot.title = element_text(lineheight=.8, hjust=0.5, vjust=-1,size= 11),
        plot.margin = unit(c(0,0.2,0,0),"cm")
  )

plot2<-ggplot(data=TSNE_miRNA, aes(x=DIM1,y=DIM2) ) +
  geom_point(aes(colour = factor(group)), size=3 , alpha =0.8 ) +
  scale_color_manual(values= c("#0072B2", "#000000","#D55E00"), labels=c("AI-preg","NB-preg", "not-preg") ,name
 =NULL) +
  scale_y_continuous(name="t-SNE dim 2")+
  scale_x_continuous(name="t-SNE dim 1")+
  ggtitle("290 miRNAs")+
  theme_bw(base_size = 11)+
  theme(aspect.ratio =1,
        axis.text=element_blank(),
        legend.position = "none",
        plot.title = element_text(lineheight=.8, hjust=0.5,vjust=-1, size= 11),
        plot.margin = unit(c(0,0,0,0.2),"cm")
  )
leg <- get_legend(
  ggplot(data=TSNE_miRNA, aes(x=DIM1,y=DIM2) ) +
  geom_point(aes(colour = factor(group)), size=3 , alpha =0.8 ) +
  scale_color_manual(values= c("#0072B2", "#000000","#D55E00"), labels=c("AI-preg","NB-preg", "non preg") ,name
 =NULL) +
  theme_bw(base_size = 12)+
  theme(legend.position = "top",legend.direction="horizontal"))


#font_size 12 for figure
#pdf(paste(result_path,"/2019_06_29_tsne_plots.pdf", sep=""), width=4.5, height=3.2)
#grid.arrange(plot1,plot2, as_ggplot(leg), layout_matrix=rbind(c(1:2),3), heights=unit(c(3,2), c("in", "mm")))
#dev.off()
grid.arrange(plot1,plot2, as_ggplot(leg), layout_matrix=rbind(c(1:2),3), heights=unit(c(3,2), c("in", "mm")))
```

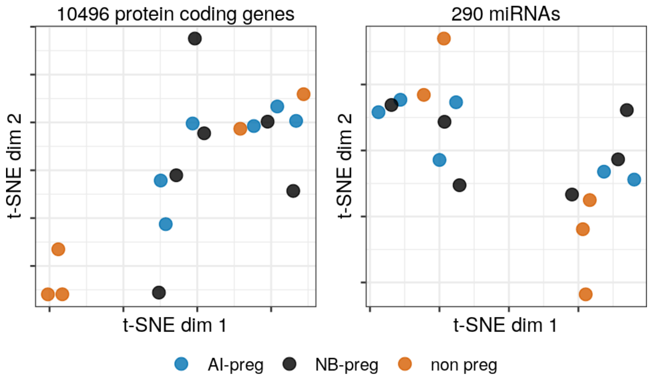


```
rm(plot1,plot2, leg, TSNE_miRNA, TSNE_mRNA, data.for.tsne.mRNA, data.for.tsne.miRNA, tsne_mRNA, tsne_miRNA)
```

Differential mRNA:mRNA coexpression associated with pregnancy outcome

Supplementary Table 1, data used for Figure 1a

Create correlation matrices for all animals.

```
correlation_all_animals<-corAndPvalue(t(log2(rpkm_pwbc+1)),method="pearson", use="pairwise.complete.obs")
correlation_all_animals_a<-melt(correlation_all_animals$cor)
correlation_all_animals_a$pvalue<-melt(correlation_all_animals$p)$value
rm(correlation_all_animals)
correlation_all_animals_a<-correlation_all_animals_a[!(correlation_all_animals_a$Var1 == correlation_all_animals_
a$Var2),]
correlation_all_animals_a<-correlation_all_animals_a[!duplicated(t(apply(correlation_all_animals_a[,c(1:2)], 1, s
ort))),]

correlation_sig_fdr0.02<-ApplyFdrCor(t(log2(rpkm_pwbc+1)), alpha=0.02, stat_test="empirical", method="LCTnorm", v
ect=TRUE)

correlation_all_animals_a$eFDR0.02<-correlation_sig_fdr0.02

correlation_all_animals_b<-correlation_all_animals_a[abs(correlation_all_animals_a$value) > 0.98,]

correlation_all_animals_b$gene_1<-annotation.ensembl.symbol$external_gene_name[match( correlation_all_animals_b$V
ar1, annotation.ensembl.symbol$ensembl_gene_id )]
correlation_all_animals_b$gene_2<-annotation.ensembl.symbol$external_gene_name[match( correlation_all_animals_b$V
ar2, annotation.ensembl.symbol$ensembl_gene_id )]
correlation_all_animals_b$Var1<-as.character(correlation_all_animals_b$Var1)
correlation_all_animals_b$Var2<-as.character(correlation_all_animals_b$Var2)

correlation_all_animals_b$gene_1<-ifelse(correlation_all_animals_b$gene_1=="", correlation_all_animals_b$Var1,cor
relation_all_animals_b$gene_1)
correlation_all_animals_b$gene_2<-ifelse(correlation_all_animals_b$gene_2=="", correlation_all_animals_b$Var2,cor
relation_all_animals_b$gene_2)
#write.table(correlation_all_animals_b, file= paste(result_path,"/2020_01_16_gene_coexpression_all_animals_b.tx
t", sep=""), append = FALSE, quote = FALSE, sep = "\t" ,row.names = FALSE)
knitr::kable(head(correlation_all_animals_b), caption = "First five rows of Supplementary table 1",format = 'pand
oc')
```

First five rows of Supplementary table 1

	Var1	Var2	value	pvalue	eFDR0.02	gene_1	gene_2
149546	ENSBTAG00000006914	ENSBTAG00000000032	0.9847809	0	TRUE	PRR3	ABI3
151225	ENSBTAG00000011444	ENSBTAG00000000032	0.9808916	0	TRUE	HINT2	ABI3
236218	ENSBTAG00000014262	ENSBTAG00000000064	0.9861767	0	TRUE	BZW2	FEN1
276256	ENSBTAG00000008954	ENSBTAG00000000077	0.9835958	0	TRUE	PSMB9	ADSL
277968	ENSBTAG00000013623	ENSBTAG00000000077	0.9842698	0	TRUE	TIMM13	ADSL
307043	ENSBTAG00000007084	ENSBTAG00000000080	0.9821463	0	TRUE	MAP3K14	GRWD1

Supplementary Table 2

Test biological processes for enrichment with genes that are highly correlated in PWBCs in all heifers.

```
all_genes<-data.frame( gene=rownames(count_pwbc), stringsAsFactors=FALSE )
rownames(all_genes)<-all_genes$gene

N_expressed_genes<-length(all_genes$gene)

annotation.genelength.biomart_vector<-gene_length[,2]
names(annotation.genelength.biomart_vector)<-gene_length[,1]

annotation.GO.BP.biomart<-annotation.GO.biomart[annotation.GO.biomart$namespace_1003=="biological_process", c(1,3
)]
annotation.GO.BP.biomart<-annotation.GO.BP.biomart[annotation.GO.BP.biomart$ensembl_gene_id %in% rownames(all_gen
es),]
annotation.GO.MF.biomart<-annotation.GO.biomart[annotation.GO.biomart$namespace_1003=="molecular_function", c(1,3
)]
annotation.GO.MF.biomart<-annotation.GO.MF.biomart[annotation.GO.MF.biomart$ensembl_gene_id %in% rownames(all_gen
es),]
```

```
test.genes<-data.frame(a=unique(c(as.character(correlation_all_animals_b$Var1),as.character(correlation_all_anima
ls_b$Var2))), stringsAsFactors=FALSE)
all_genes_numeric<-as.integer(all_genes$gene %in%test.genes$a)
names(all_genes_numeric)<-all_genes$gene

N_DEGs<-length(test.genes$a)

set.seed(9830)
pwf<-nullp(all_genes_numeric, bias.data=annotation.genelength.biomart_vector, plot.fit=FALSE )
GO_BP_Cats_mRNA_all_heifers<-goseq(pwf,gene2cat=annotation.GO.BP.biomart, method ="Sampling", repcnt = 7000, use_
genes_without_cat=FALSE)
GO_BP_Cats_mRNA_all_heifers<-GO_BP_Cats_mRNA_all_heifers[GO_BP_Cats_mRNA_all_heifers$numDEInCat>4,]
GO_BP_Cats_mRNA_all_heifers$BY_FDR<-p.adjust(GO_BP_Cats_mRNA_all_heifers$over_represented_pvalue, method ="fdr")
GO_BP_Cats_mRNA_all_heifers<-GO_BP_Cats_mRNA_all_heifers[with(GO_BP_Cats_mRNA_all_heifers, order(BY_FDR,over_repr
esented_pvalue, -numDEInCat)), ]
#head(GO_BP_Cats_mRNA_all_heifers, n=20)

GO_BP_Cats_mRNA_all_heifers$fold_enrichment<-(GO_BP_Cats_mRNA_all_heifers$numDEInCat/N_DEGs)/(GO_BP_Cats_mRNA_all
_heifers$numInCat/N_expressed_genes)
annotation.GO.BP.biomart_testgenes<-annotation.GO.BP.biomart[annotation.GO.BP.biomart$ensembl_gene_id %in% test.g
enes$a, ]
GO_BP_Cats_mRNA_all_heifers<-merge(GO_BP_Cats_mRNA_all_heifers,annotation.GO.BP.biomart_testgenes, by.x="categor
y", by.y="go_id", all.x=TRUE, all.y=FALSE)
GO_BP_Cats_mRNA_all_heifers<-merge(GO_BP_Cats_mRNA_all_heifers, annotation.ensembl.symbol, by.x="ensembl_gene_id"
, by.y="ensembl_gene_id", all=FALSE, all.x=TRUE, all.y=FALSE)
GO_BP_Cats_mRNA_all_heifers<-GO_BP_Cats_mRNA_all_heifers[with(GO_BP_Cats_mRNA_all_heifers, order(BY_FDR,term)), ]
GO_BP_Cats_mRNA_all_heifers<-GO_BP_Cats_mRNA_all_heifers[GO_BP_Cats_mRNA_all_heifers$BY_FDR<0.2,]

#write.table(GO_BP_Cats_mRNA_all_heifers, file= paste(result_path,"2019_11_10_GO_BP_Cats_mRNA_cor_above98_all_hei
fers.txt", sep="/"), append = FALSE, quote = FALSE, sep = "\t" ,row.names = FALSE)
rm( correlation_all_animals_a)
```

```
knitr::kable(head(GO_BP_Cats_mRNA_all_heifers), caption = "First five rows of Supplementary Table 2")
```

First five rows of Supplementary Table 2

	ensembl_gene_id	category	over_represented_pvalue	under_represented_pvalue	numDEInCat	numInCat	term
36	ENSBTAG00000000359	GO:0002183 (GO:0002183)	0.0001428	1	8	13	cytoplasm translatior initiation
170	ENSBTAG00000001988	GO:0002183 (GO:0002183)	0.0001428	1	8	13	cytoplasm translatior initiation
212	ENSBTAG00000002734	GO:0002183 (GO:0002183)	0.0001428	1	8	13	cytoplasm translatior initiation
369	ENSBTAG00000004861	GO:0002183 (GO:0002183)	0.0001428	1	8	13	cytoplasm translatior initiation

	ensembl_gene_id	category	over_represented_pvalue	under_represented_pvalue	numDEInCat	numInCat	term
553	ENSBTAG00000006543	GO:0002183 (GO:0002183)	0.0001428	1	8	13	cytoplasm translatior initiation
885	ENSBTAG00000010790	GO:0002183 (GO:0002183)	0.0001428	1	8	13	cytoplasm translatior initiation

Create correlation matrices for AI pregnant, NB pregnant and non pregnant animals.

```
correlation_AIpreg<-corAndPvalue(t(log2(rpkm_AIpreg+1)),method="pearson", use="pairwise.complete.obs")
correlation_AIpreg_a<-melt(correlation_AIpreg$cor)
correlation_AIpreg_a$pvalue<-melt(correlation_AIpreg$p)$value
rm(correlation_AIpreg)

correlation_preg_NB<-corAndPvalue(t(log2(rpkm_NBpreg+1)),method="pearson", use="pairwise.complete.obs")
correlation_preg_NB_a<-melt(correlation_preg_NB$cor)
correlation_preg_NB_a$pvalue<-melt(correlation_preg_NB$p)$value
rm(correlation_preg_NB)

correlation_NOTpreg<-corAndPvalue(t(log2(rpkm_NOTpreg+1)),method="pearson", use="pairwise.complete.obs")
correlation_NOTpreg_a<-melt(correlation_NOTpreg$cor)
correlation_NOTpreg_a$pvalue<-melt(correlation_NOTpreg$p)$value
rm(correlation_NOTpreg)
```

Merge the correlation matrices

```
pearson_correlation<-cbind(correlation_AIpreg_a, correlation_preg_NB_a, correlation_NOTpreg_a)
pearson_correlation<-pearson_correlation[,c(1:4,7,8,11,12)]
colnames(pearson_correlation)<-c('gene_1', 'gene_2', 'cor_AI', 'pvalue_AI', 'cor_NB', 'pvalue_NB','cor_NP', 'pval
ue_NP')
#head(pearson_correlation)

pearson_correlation<-pearson_correlation[!(pearson_correlation$gene_1 == pearson_correlation$gene_2),]
dim(pearson_correlation) # 110155520
```

```
## [1] 110155520         8
```

```
pearson_correlation<-pearson_correlation[!duplicated(t(apply(pearson_correlation[,c(1:2)], 1, sort))),]
dim(pearson_correlation) # 55077760
```

```
## [1] 55077760        8
```

Supplementary Fig. 1a,b

Calculate the empirical false discovery rate for co-expression and differential coexpression.

```
permutation_matrix<-matrix(,nrow=200100,ncol=17)
#head(permutation_matrix)
#dim(permutation_matrix)

for (i in seq(1:200100)){
  sampling <- sample(1:17,17, replace = FALSE)
  if ( ! identical(sampling , c(1:17))){
    permutation_matrix[i,]<-sample(1:17,17, replace = FALSE)
  }}

permutation_matrix<-permutation_matrix[!duplicated(permutation_matrix),]
dim(permutation_matrix)
head(permutation_matrix)

permutation_matrix<-permutation_matrix[sample(1:dim(permutation_matrix)[1], 5000),]
rand<-dim(permutation_matrix)[1]

#diff.coexp<-seq(0.005, 0.05, 0.005)
diff.coexp<-seq(1.6, 2, 0.05)

results <- filebacked.big.matrix(length(diff.coexp),rand, type="double", init=0, separated=FALSE,
                                 backingfile="incidence_matrix.bin",
                                 descriptor="incidence_matrix.desc")
mdesc_result<- describe(results)

cl <- makeCluster(10)
registerDoParallel(cl)

results[,]<-foreach(i = diff.coexp, .combine='rbind',.packages=c("reshape2","bigmemory"), .inorder=TRUE, .verbose
=TRUE ) %:%
            foreach(j = 1:rand, .combine='cbind',.packages=c("reshape2","bigmemory"), .inorder=FALSE, .verbose=TR
UE ) %dopar% {

   rpkm_pwbc_scramble<-log2(rpkm_pwbc[,permutation_matrix[j,]] +1)
   cor_1<-(cor(t(rpkm_pwbc_scramble[,1:6])))
   cor_2<-(cor(t(rpkm_pwbc_scramble[,7:11])))
   length(which(abs(cor_1-cor_2) > i))/2
  }
stopCluster(cl)

total.rand <-  5000 * 55077760

qvalue<-data.frame(diff_correlation = diff.coexp,
                   e.pvalue= (rowSums(results[,]+1))/(total.rand+1),
                   e.pvalue.round= round((rowSums(results[,]+1))/(total.rand+1) ,6))

write.table(qvalue, file= paste(result_path,"/2019_06_26_gene_coexpression_inverted_empiricalFDR.txt", sep=""), a
ppend = FALSE, quote = FALSE, sep = "\t" ,row.names = FALSE)

rm(results)
system("rm incidence_matrix.bin")
system("rm incidence_matrix.desc")

coexp<-seq(0.9, 1, 0.01)

results <- filebacked.big.matrix(length(coexp),rand, type="double", init=0, separated=FALSE,
                                 backingfile="incidence_matrix.bin",
                                 descriptor="incidence_matrix.desc")
mdesc_result<- describe(results)

cl <- makeCluster(10)
registerDoParallel(cl)

results[,]<-foreach(i = coexp, .combine='rbind',.packages=c("reshape2","bigmemory"), .inorder=TRUE, .verbose=TRUE
) %:%
            foreach(j = 1:rand, .combine='cbind',.packages=c("reshape2","bigmemory"), .inorder=FALSE, .verbose=TR
UE ) %dopar% {

   rpkm_pwbc_scramble<-log2(rpkm_pwbc[,permutation_matrix[j,]] +1)
   cor_1<-(cor(t(rpkm_pwbc_scramble[,1:6])))
   length(which(abs(cor_1) > i))/2
  }
stopCluster(cl)

total.rand <-  5000 * 55077760

qvalue<-data.frame(correlation = coexp,
                   e.pvalue= (rowSums(results[,]+1))/(total.rand+1),
                   e.pvalue.round= round((rowSums(results[,]+1))/(total.rand+1) ,6))

#write.table(qvalue, file= paste(result_path,"/2019_06_27_gene_coexpression_empiricalFDR.txt", sep=""), append =
```

```
 FALSE, quote = FALSE, sep = "\t" ,row.names = FALSE)

rm(results)
system("rm incidence_matrix.bin")
system("rm incidence_matrix.desc")
rm(permutation_matrix)
```

```
eFDR_coexpression<-read.delim(paste(result_path,"/2019_06_27_gene_coexpression_empiricalFDR.txt", sep=""), row.na
mes=NULL,header =TRUE, stringsAsFactors =FALSE)
eFDR_coexpression_inverted<-read.delim(paste(result_path,"/2019_06_26_gene_coexpression_inverted_empiricalFDR.tx
t", sep=""), row.names=NULL,header =TRUE, stringsAsFactors =FALSE)
```

```
font_size<-9

plot1<-ggplot()+
  geom_point(data=eFDR_coexpression, aes(x=correlation , y=e.pvalue.round),color="black", size=3, shape=16)+
  geom_line(data=eFDR_coexpression, aes(x=correlation , y=e.pvalue.round),color="black", size=0.1,linetype=3)+
  scale_y_continuous(name="empirical FDR", limits = c(0, 0.1), breaks=seq(0,0.1, 0.005))+
  scale_x_continuous(name="correlation (r)", limits = c(0.9, 1), breaks=seq(0.9,1, 0.01))+
  ggtitle("Gene coexpression within a group of heifers \n of same reproductive outcome")+
  theme_bw()+
  theme(panel.grid= element_blank(),
        panel.background = element_blank(),
        panel.grid.minor = element_blank(),
        panel.grid.major = element_line(color="lightgray"),
        plot.background = element_blank(),
        axis.title=element_text(color="black", size=font_size),
        axis.text=element_text(color="black", size=font_size),
        panel.spacing = unit(c(0.4,0.4,0.4,0.4),"cm"),
        plot.margin = unit(c(0.5,0.5,0.5,0.5),"cm"),
        legend.position="none",
        plot.title = element_text(lineheight=.8, hjust=0.5, size= font_size))

plot2<-ggplot()+
  geom_point(data=eFDR_coexpression_inverted, aes(x=diff_correlation , y=e.pvalue.round),color="black", size=3, s
hape=16)+
  geom_line(data=eFDR_coexpression_inverted, aes(x=diff_correlation , y=e.pvalue.round),color="black", size=0.1,l
inetype=3)+
  scale_y_continuous(name="empirical FDR", limits = c(0, 0.01), breaks=seq(0,0.01, 0.001))+
  scale_x_continuous(name="absolute difference between correlation values", limits = c(1.5, 2), breaks=seq(1.5, 2
, 0.05))+
  ggtitle("Inverted gene coexpression \n between groups of heifers")+
  theme_bw()+
  theme(panel.grid= element_blank(),
        panel.background = element_blank(),
        panel.grid.minor = element_blank(),
        panel.grid.major = element_line(color="lightgray"),
        plot.background = element_blank(),
        axis.title=element_text(color="black", size=font_size),
        axis.text=element_text(color="black", size=font_size),
        panel.spacing = unit(c(0.4,0.4,0.4,0.4),"cm"),
        plot.margin = unit(c(0.5,0.5,0.5,0.5),"cm"),
        legend.position="none",
        plot.title = element_text(lineheight=.8, hjust=0.5 , size= font_size ))

#font_size 12 for figure
#pdf(paste(result_path,"2019_11_10_eFDR_plots.pdf", sep="/"), width=10, height=5)
#grid.arrange(plot1,plot2, ncol = 2)
#dev.off()

grid.arrange(plot1,plot2, ncol = 2)
```

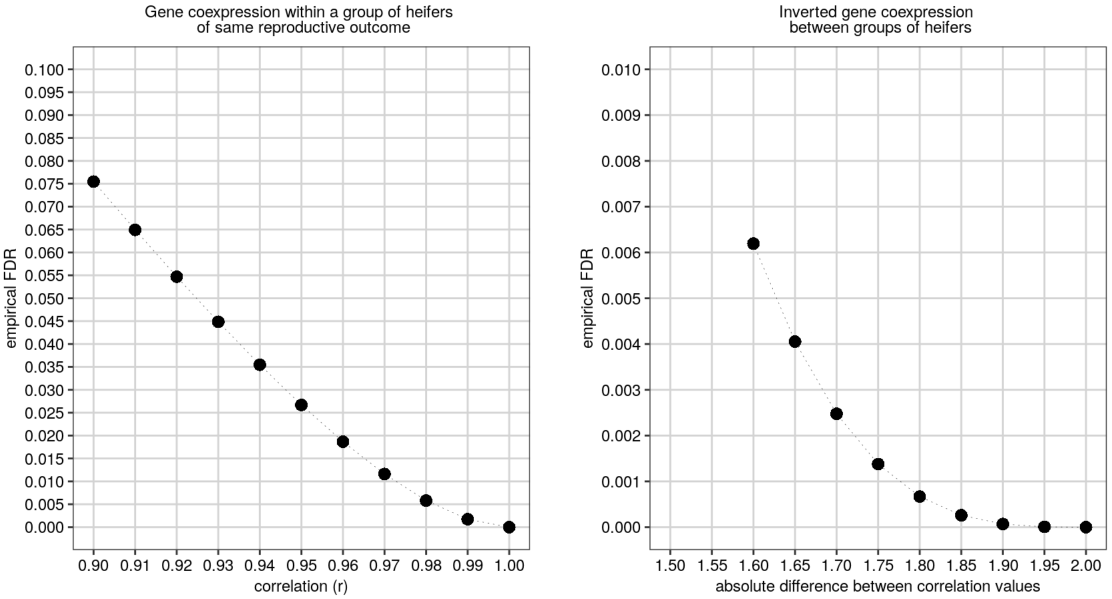

Perform calculations to determine differential coexpression.

```
pearson_correlation$subtraction_cor_AI_cor_NP<-pearson_correlation$cor_AI - pearson_correlation$cor_NP
pearson_correlation$subtraction_cor_NB_cor_NP<-pearson_correlation$cor_NB - pearson_correlation$cor_NP
pearson_correlation$subtraction_cor_AI_cor_NB<-pearson_correlation$cor_AI - pearson_correlation$cor_NB


pearson_correlation$result_cor_AI_cor_NP<-ifelse(abs(pearson_correlation$subtraction_cor_AI_cor_NP) > 1.95 , "inv
erted",
                                   ifelse(abs(pearson_correlation$cor_AI) >0.99 & (pearson_correlation$cor_NP > -
0.1 & pearson_correlation$cor_NP < 0.1) , "loss",
                                          ifelse( (pearson_correlation$cor_AI > -0.1 & pearson_correlation$cor_AI
< 0.1)  &  abs(pearson_correlation$cor_NP) >0.99, "gain" , "nothing")))

pearson_correlation$result_cor_NB_cor_NP<-ifelse(abs(pearson_correlation$subtraction_cor_NB_cor_NP) > 1.95 , "inv
erted",
                                   ifelse(abs(pearson_correlation$cor_NB) >0.99 & (pearson_correlation$cor_NP > -
0.1 & pearson_correlation$cor_NP < 0.1) , "loss",
                                          ifelse( (pearson_correlation$cor_NB > -0.1 & pearson_correlation$cor_NB
< 0.1)  &  abs(pearson_correlation$cor_NP) >0.99, "gain" , "nothing")))

pearson_correlation$result_cor_AI_cor_NB<-ifelse(abs(pearson_correlation$subtraction_cor_AI_cor_NB) > 1.95 , "inv
erted",
                                   ifelse(abs(pearson_correlation$cor_AI) >0.99 & (pearson_correlation$cor_NB > -
0.1 & pearson_correlation$cor_NB < 0.1) , "loss",
                                          ifelse( (pearson_correlation$cor_AI > -0.1 & pearson_correlation$cor_AI
< 0.1)  &  abs(pearson_correlation$cor_NB) >0.99, "gain" , "nothing")))


pearson_correlation$result_cor_AI_and_NB_cor_NP<-ifelse( (abs(pearson_correlation$subtraction_cor_AI_cor_NP) + ab
s(pearson_correlation$subtraction_cor_NB_cor_NP)) / 2 > 1.9 , "inverted",
                                   ifelse( (abs(pearson_correlation$cor_AI) >0.99  & abs(pearson_correlation$cor_
NB) >0.99)  & (pearson_correlation$cor_NP > -0.1 & pearson_correlation$cor_NP < 0.1) , "loss",
                                          ifelse( (pearson_correlation$cor_AI > -0.1 & pearson_correlation$cor_AI
< 0.1 & pearson_correlation$cor_NB > -0.1 & pearson_correlation$cor_NB < 0.1 )  &  abs(pearson_correlation$cor_N
P) > 0.99, "gain" , "nothing")))

pearson_correlation$result_cor_AI_and_NB_cor_NP_a<-ifelse( (abs(pearson_correlation$subtraction_cor_AI_cor_NP) +
 abs(pearson_correlation$subtraction_cor_NB_cor_NP)) / 2 > 1.9 , "inverted",
                                   ifelse( (pearson_correlation$cor_AI >0.99  & pearson_correlation$cor_NB >0.99)
& (pearson_correlation$cor_NP > -0.1 & pearson_correlation$cor_NP < 0.1) , "loss_positive",
                                   ifelse( (pearson_correlation$cor_AI < -0.99  & pearson_correlation$cor_NB < -
0.99)  & (pearson_correlation$cor_NP > -0.1 & pearson_correlation$cor_NP < 0.1) , "loss_negative",
                                   ifelse( (pearson_correlation$cor_AI > -0.1 & pearson_correlation$cor_AI < 0.1
 & pearson_correlation$cor_NB > -0.1 & pearson_correlation$cor_NB < 0.1 )  &  pearson_correlation$cor_NP > 0.99,
"gain_positive" ,
                                   ifelse( (pearson_correlation$cor_AI > -0.1 & pearson_correlation$cor_AI < 0.1
 & pearson_correlation$cor_NB > -0.1 & pearson_correlation$cor_NB < 0.1 )  &  pearson_correlation$cor_NP < -0.99,
"gain_negative" ,"nothing")))))

#write.table(pearson_correlation, file= paste(result_path,"/2020_01_28_differential_gene_coexpression.txt", sep
=""), append = FALSE, quote = FALSE, sep = "\t" ,row.names = FALSE)


table(pearson_correlation$result_cor_AI_and_NB_cor_NP)
```

```
##
##     gain inverted     loss  nothing
##     1424       23        2 55076311
```

```
table(pearson_correlation$result_cor_AI_and_NB_cor_NP_a)
```

```
##
## gain_negative gain_positive      inverted loss_positive       nothing
##           716           708            23             2      55076311
```

Figures 1b,c

Subset the matrix of correlation based on the results from pearson_correlation$result_cor_AI_and_NB_cor_NP_a

```
correlation_AIpreg<-cor(t(log2(rpkm_AIpreg+1)),method="pearson", use="pairwise.complete.obs")
correlation_preg_NB<-cor(t(log2(rpkm_NBpreg+1)),method="pearson", use="pairwise.complete.obs")
correlation_NOTpreg<-cor(t(log2(rpkm_NOTpreg+1)),method="pearson", use="pairwise.complete.obs")

correlation_AI_NB_preg<-(correlation_AIpreg + correlation_preg_NB)/2

pearson_correlation_a<-pearson_correlation[!(pearson_correlation$result_cor_AI_and_NB_cor_NP_a == "nothing"), ]

#write.table(pearson_correlation_a, file= paste(result_path,"/2020_01_28_differential_gene_coexpression_only_sign
ificant.txt", sep=""), append = FALSE, quote = FALSE, sep = "\t" ,row.names = FALSE)


correlation_AI_NB_preg<-correlation_AI_NB_preg[row.names(correlation_AI_NB_preg) %in% unique(c(as.character(pears
on_correlation_a$gene_1), as.character(pearson_correlation_a$gene_2))),
                                              colnames(correlation_AI_NB_preg)  %in% unique(c(as.character(pearso
n_correlation_a$gene_1), as.character(pearson_correlation_a$gene_2)))]
correlation_NOTpreg<-correlation_NOTpreg[row.names(correlation_NOTpreg) %in% unique(c(as.character(pearson_correl
ation_a$gene_1), as.character(pearson_correlation_a$gene_2))),
                                        colnames(correlation_NOTpreg)  %in% unique(c(as.character(pearson_correla
tion_a$gene_1), as.character(pearson_correlation_a$gene_2)))]



#rm(correlation_AIpreg,correlation_preg_NB,correlation_NOTpreg)

dissimilarity<-as.matrix(parDist(x= correlation_AI_NB_preg, method = "euclidean", diag = TRUE, upper = FALSE))
rownames(dissimilarity)<-colnames(correlation_AI_NB_preg)
colnames(dissimilarity)<-colnames(correlation_AI_NB_preg)
geneTree <- flashClust(as.dist(dissimilarity), method = "complete")
rm(dissimilarity)

subtraction_matrix <- correlation_NOTpreg - correlation_AI_NB_preg

correlation_AI_NB_preg<-correlation_AI_NB_preg[geneTree$order,geneTree$order ]
subtraction_matrix<-subtraction_matrix[geneTree$order,geneTree$order ]


subtraction_matrix[(subtraction_matrix >-1.8 & subtraction_matrix < -1.09) |  (subtraction_matrix > -0.89 & subtr
action_matrix < 0.89) | (subtraction_matrix > 1.09 & subtraction_matrix < 1.8)] <- 0

subtraction_matrix[upper.tri(subtraction_matrix)]<-0

correlation_AI_NB_preg[upper.tri(correlation_AI_NB_preg)]<-0
```

```
ht<-Heatmap(correlation_AI_NB_preg,
        name="correlation",
        cluster_rows= FALSE,
        cluster_columns = FALSE,
        show_row_names = FALSE,
        show_column_names = FALSE,
        col = colorRamp2(c(-2, -1,0, 1, 2), c("midnightblue","cyan1", "white", "magenta", "mediumvioletred"), spa
ce = "LAB"),
        heatmap_legend_param = list(title = "correlation  ",title_position = "lefttop",legend_direction = "horizo
ntal", title_gp = gpar(col = "black", fontsize = 5),labels_gp = gpar(col = "black", fontsize = 4 ),grid_height=un
it(0.5, "mm"),legend_width=unit(1.5, "cm"))
)
#png(paste(result_path,"/2019_07_08_AI_NB_heatmap_a.png", sep=""),width = 570, height = 625,res = 300)
#draw(ht, heatmap_legend_side = "bottom")
#dev.off()
draw(ht, heatmap_legend_side = "bottom")
```

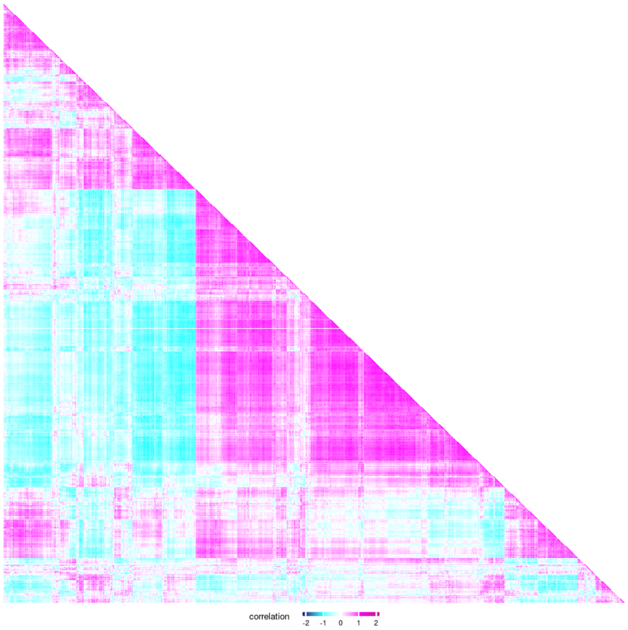


```
ht<-Heatmap(subtraction_matrix,
        name="correlation",
        cluster_rows= FALSE,
        cluster_columns = FALSE,
        show_row_names = FALSE,
        show_column_names = FALSE,
        col = colorRamp2(c(-2, -1,0, 1, 2), c("midnightblue","cyan1", "white", "magenta", "mediumvioletred"), spa
ce = "LAB"),
        heatmap_legend_param = list(title = "correlation  ",title_position = "lefttop",legend_direction = "horizo
ntal", title_gp = gpar(col = "black", fontsize = 5),labels_gp = gpar(col = "black", fontsize = 4 ),grid_height=un
it(0.5, "mm"),legend_width=unit(1.5, "cm"))
)

#png(paste(result_path,"/2019_07_08_non_preg_differential_coex_heatmap.png", sep=""),width = 570, height = 625,re
s = 300)
#draw(ht, heatmap_legend_side = "bottom")
#dev.off()
draw(ht, heatmap_legend_side = "bottom")
```

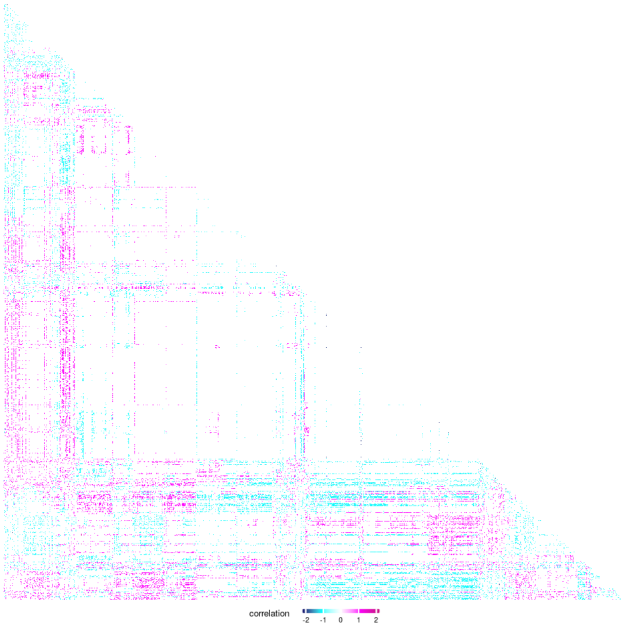

Test biological processes for enrichment with mRNAs that formed negative coexpression and calculate fold change enrichment. (AI and NB vs NP)

```
pearson_correlation_a<-pearson_correlation[pearson_correlation$result_cor_AI_and_NB_cor_NP_a == "gain_positive",]
test.genes<-data.frame(a=unique(c(as.character(pearson_correlation_a$gene_1),as.character(pearson_correlation_a$g
ene_2))), stringsAsFactors=FALSE)
all_genes_numeric<-as.integer(all_genes$gene %in%test.genes$a)
names(all_genes_numeric)<-all_genes$gene

N_DEGs<-length(test.genes$a)

set.seed(9830)
pwf<-nullp(all_genes_numeric, bias.data=annotation.genelength.biomart_vector, plot.fit=FALSE )
GO_BP_Cats_mRNA_AI_and_NB_and_NP<-goseq(pwf,gene2cat=annotation.GO.BP.biomart, method ="Sampling", repcnt = 7000,
use_genes_without_cat=FALSE)
GO_BP_Cats_mRNA_AI_and_NB_and_NP<-GO_BP_Cats_mRNA_AI_and_NB_and_NP[GO_BP_Cats_mRNA_AI_and_NB_and_NP$numDEInCat>4
,]
GO_BP_Cats_mRNA_AI_and_NB_and_NP$BY_FDR<-p.adjust(GO_BP_Cats_mRNA_AI_and_NB_and_NP$over_represented_pvalue, metho
d ="fdr")
GO_BP_Cats_mRNA_AI_and_NB_and_NP<-GO_BP_Cats_mRNA_AI_and_NB_and_NP[with(GO_BP_Cats_mRNA_AI_and_NB_and_NP, order(B
Y_FDR,over_represented_pvalue, -numDEInCat)), ]
head(GO_BP_Cats_mRNA_AI_and_NB_and_NP, n=20)

GO_BP_Cats_mRNA_AI_and_NB_and_NP$fold_enrichment<-(GO_BP_Cats_mRNA_AI_and_NB_and_NP$numDEInCat/N_DEGs)/(GO_BP_Cat
s_mRNA_AI_and_NB_and_NP$numInCat/N_expressed_genes)
annotation.GO.BP.biomart_testgenes<-annotation.GO.BP.biomart[annotation.GO.BP.biomart$ensembl_gene_id %in% test.g
enes$a, ]
GO_BP_Cats_mRNA_AI_and_NB_and_NP<-merge(GO_BP_Cats_mRNA_AI_and_NB_and_NP,annotation.GO.BP.biomart_testgenes, by.x
="category", by.y="go_id", all.x=TRUE, all.y=FALSE)
GO_BP_Cats_mRNA_AI_and_NB_and_NP<-merge(GO_BP_Cats_mRNA_AI_and_NB_and_NP, annotation.ensembl.symbol, by.x="ensemb
l_gene_id", by.y="ensembl_gene_id", all=FALSE, all.x=TRUE, all.y=FALSE)
GO_BP_Cats_mRNA_AI_and_NB_and_NP<-GO_BP_Cats_mRNA_AI_and_NB_and_NP[with(GO_BP_Cats_mRNA_AI_and_NB_and_NP, order(B
Y_FDR,term)), ]
GO_BP_Cats_mRNA_AI_and_NB_and_NP<-GO_BP_Cats_mRNA_AI_and_NB_and_NP[GO_BP_Cats_mRNA_AI_and_NB_and_NP$BY_FDR<0.2,]
```

Supplementary Table 4

```
pearson_correlation_a<-pearson_correlation[pearson_correlation$result_cor_AI_and_NB_cor_NP_a == "gain_negative",]
test.genes<-data.frame(a=unique(c(as.character(pearson_correlation_a$gene_1),as.character(pearson_correlation_a$g
ene_2))), stringsAsFactors=FALSE)
all_genes_numeric<-as.integer(all_genes$gene %in%test.genes$a)
names(all_genes_numeric)<-all_genes$gene

N_DEGs<-length(test.genes$a)

set.seed(9830)
pwf<-nullp(all_genes_numeric, bias.data=annotation.genelength.biomart_vector, plot.fit=FALSE )
GO_BP_Cats_mRNA_AI_and_NB_and_NP<-goseq(pwf,gene2cat=annotation.GO.BP.biomart, method ="Sampling", repcnt = 7000,
use_genes_without_cat=FALSE)
GO_BP_Cats_mRNA_AI_and_NB_and_NP<-GO_BP_Cats_mRNA_AI_and_NB_and_NP[GO_BP_Cats_mRNA_AI_and_NB_and_NP$numDEInCat>4
,]
GO_BP_Cats_mRNA_AI_and_NB_and_NP$BY_FDR<-p.adjust(GO_BP_Cats_mRNA_AI_and_NB_and_NP$over_represented_pvalue, metho
d ="fdr")
GO_BP_Cats_mRNA_AI_and_NB_and_NP<-GO_BP_Cats_mRNA_AI_and_NB_and_NP[with(GO_BP_Cats_mRNA_AI_and_NB_and_NP, order(B
Y_FDR,over_represented_pvalue, -numDEInCat)), ]
#head(GO_BP_Cats_mRNA_AI_and_NB_and_NP, n=20)

GO_BP_Cats_mRNA_AI_and_NB_and_NP$fold_enrichment<-(GO_BP_Cats_mRNA_AI_and_NB_and_NP$numDEInCat/N_DEGs)/(GO_BP_Cat
s_mRNA_AI_and_NB_and_NP$numInCat/N_expressed_genes)
annotation.GO.BP.biomart_testgenes<-annotation.GO.BP.biomart[annotation.GO.BP.biomart$ensembl_gene_id %in% test.g
enes$a, ]
GO_BP_Cats_mRNA_AI_and_NB_and_NP<-merge(GO_BP_Cats_mRNA_AI_and_NB_and_NP,annotation.GO.BP.biomart_testgenes, by.x
="category", by.y="go_id", all.x=TRUE, all.y=FALSE)
GO_BP_Cats_mRNA_AI_and_NB_and_NP<-merge(GO_BP_Cats_mRNA_AI_and_NB_and_NP, annotation.ensembl.symbol, by.x="ensemb
l_gene_id", by.y="ensembl_gene_id", all=FALSE, all.x=TRUE, all.y=FALSE)
GO_BP_Cats_mRNA_AI_and_NB_and_NP<-GO_BP_Cats_mRNA_AI_and_NB_and_NP[with(GO_BP_Cats_mRNA_AI_and_NB_and_NP, order(B
Y_FDR,term)), ]
GO_BP_Cats_mRNA_AI_and_NB_and_NP<-GO_BP_Cats_mRNA_AI_and_NB_and_NP[GO_BP_Cats_mRNA_AI_and_NB_and_NP$BY_FDR<0.1,]
#write.table(GO_BP_Cats_mRNA_AI_and_NB_and_NP, paste(result_path, "2019_12_06_GO_BP_Cats_mRNA_diff_coexpression_g
ain_neg_AI_and_NB_and_NP.txt", sep="/"), quote=FALSE, sep="\t", row.names=FALSE)
```

```
knitr::kable(head(GO_BP_Cats_mRNA_AI_and_NB_and_NP), caption = "First five rows of Supplementary Table 4",format
 = 'pandoc')
```

First five rows of Supplementary Table 4

ensembl_gene_id	category	over_represented_pvalue	under_represented_pvalue	numDEInCat	numInCat	term

	ensembl_gene_id	category	over_represented_pvalue	under_represented_pvalue	numDEInCat	numInCat	term
10	ENSBTAG00000000131	GO:0006508 (GO:0006508)	0.0005713	0.9998572	38	232	proteolysis
51	ENSBTAG00000000725	GO:0006508 (GO:0006508)	0.0005713	0.9998572	38	232	proteolysis
83	ENSBTAG00000001134	GO:0006508 (GO:0006508)	0.0005713	0.9998572	38	232	proteolysis
104	ENSBTAG00000001353	GO:0006508 (GO:0006508)	0.0005713	0.9998572	38	232	proteolysis
230	ENSBTAG00000003039	GO:0006508 (GO:0006508)	0.0005713	0.9998572	38	232	proteolysis
266	ENSBTAG00000003395	GO:0006508 (GO:0006508)	0.0005713	0.9998572	38	232	proteolysis

Figure 1d

```
proteolysis_genes<-GO_BP_Cats_mRNA_AI_and_NB_and_NP[GO_BP_Cats_mRNA_AI_and_NB_and_NP$term == "proteolysis", 1]
pearson_correlation_b<-pearson_correlation_a[pearson_correlation_a$gene_1 %in% proteolysis_genes | pearson_correl
ation_a$gene_2 %in% proteolysis_genes ,]
pearson_correlation_b<-pearson_correlation_b[with(pearson_correlation_b, order(cor_NP)), ]

data_for_chart<-data.frame()
data_for_chart_a<-data.frame()
for(i in 1:6 ){
  expression_gene_1_AI<-rpkm_AIpreg[row.names(rpkm_AIpreg)==as.character(pearson_correlation_b$gene_1)[i],]
  expression_gene_2_AI<-rpkm_AIpreg[row.names(rpkm_AIpreg)==as.character(pearson_correlation_b$gene_2)[i],]
  expression_gene_1_NB<-rpkm_NBpreg[row.names(rpkm_NBpreg)==as.character(pearson_correlation_b$gene_1)[i],]
  expression_gene_2_NB<-rpkm_NBpreg[row.names(rpkm_NBpreg)==as.character(pearson_correlation_b$gene_2)[i],]
  expression_gene_1_NP<-rpkm_NOTpreg[row.names(rpkm_NOTpreg)==as.character(pearson_correlation_b$gene_1)[i],]
  expression_gene_2_NP<-rpkm_NOTpreg[row.names(rpkm_NOTpreg)==as.character(pearson_correlation_b$gene_2)[i],]

  symbol_gene_1<-annotation.ensembl.symbol[annotation.ensembl.symbol$ensembl_gene_id == as.character(pearson_corr
elation_b$gene_1)[i] , 2]
  symbol_gene_2<-annotation.ensembl.symbol[annotation.ensembl.symbol$ensembl_gene_id == as.character(pearson_corr
elation_b$gene_2)[i] , 2]
  chart<-i

  data_for_chart<-data.frame(gene_1=c(expression_gene_1_AI,expression_gene_1_NB,expression_gene_1_NP) ,
                             gene_2=c(expression_gene_2_AI,expression_gene_2_NB,expression_gene_2_NP),
                             symbol_gene_1,symbol_gene_2 ,chart,
                             group=rep(c("AI", "NB", "NP"), c(length(expression_gene_1_AI),length(expression_gene
_1_NB),length(expression_gene_1_NP))))

  data_for_chart_a<-rbind(data_for_chart_a,data_for_chart)
}

font_axis<-5
font_title<-8
ptsize<-0.05

plots<-list()
k<-1
for (j in c(1:6)){
  data_for_chart_b<-data_for_chart_a[data_for_chart_a$chart %in% j,]

plot<-ggplot(data_for_chart_b, aes(x=gene_1,y=gene_2))+
  geom_point()+
  geom_smooth(method=lm, size=ptsize, fill="lightgray")+
  scale_y_continuous(name=data_for_chart_b$symbol_gene_1[1])+
  scale_x_continuous(name=data_for_chart_b$symbol_gene_2[1])+
  facet_wrap(~group, ncol=3, scales="free")+
  theme(aspect.ratio = 1,
            panel.grid.major = element_blank(),
            panel.grid.minor = element_blank(),
            panel.background = element_blank(),
            plot.background  = element_blank(),
            axis.text = element_text( colour = 'black',size = font_axis),
            axis.title = element_text( colour = 'black' ,size = font_title, face="italic"),
            axis.ticks = element_line(size=0.1),
            panel.spacing.y = unit(0, "mm"),
            panel.spacing.x = unit(1.5, "mm"),
            legend.position="none",
            axis.line=element_line(size = 0.1, colour = "black"),
            strip.background=element_blank(),
            strip.text.x = element_blank())

 plots[[k]]<-plot
  k<-k+1
}

#png(filename =paste(result_path,"2019_11_14_plot_diff_cor_AI_NB_and_NP_gain_negative.png", sep="/"), width = 5.
2, height =3, units = 'in', res = 300,bg = "transparent")
#ggarrange(plotlist=plots, ncol=2, nrow=3)
#dev.off()
ggarrange(plotlist=plots, ncol=2, nrow=3)
```

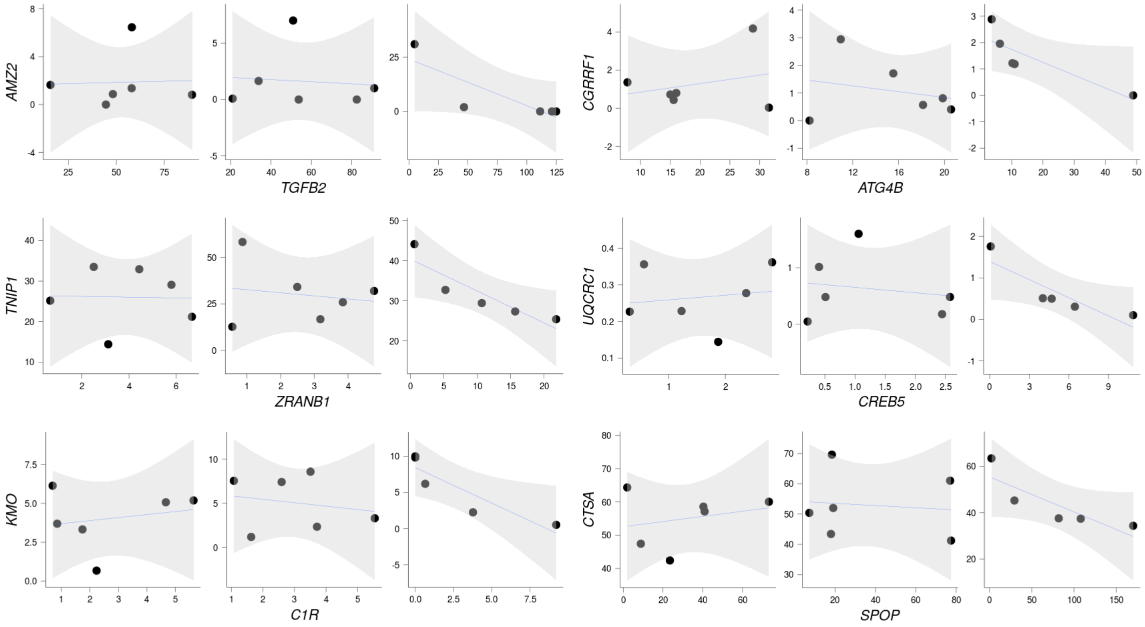

Supplementary Table 5

```
pearson_correlation_a<-pearson_correlation[pearson_correlation$result_cor_AI_and_NB_cor_NP_a == "inverted",]
test.genes<-data.frame(a=unique(c(as.character(pearson_correlation_a$gene_1),as.character(pearson_correlation_a$g
ene_2))), stringsAsFactors=FALSE)
all_genes_numeric<-as.integer(all_genes$gene %in%test.genes$a)
names(all_genes_numeric)<-all_genes$gene

N_DEGs<-length(test.genes$a)

set.seed(9830)
pwf<-nullp(all_genes_numeric, bias.data=annotation.genelength.biomart_vector, plot.fit=FALSE )
GO_BP_Cats_mRNA_AI_and_NB_and_NP<-goseq(pwf,gene2cat=annotation.GO.BP.biomart, method ="Sampling", repcnt = 7000,
use_genes_without_cat=FALSE)
GO_BP_Cats_mRNA_AI_and_NB_and_NP<-GO_BP_Cats_mRNA_AI_and_NB_and_NP[GO_BP_Cats_mRNA_AI_and_NB_and_NP$numDEInCat>4
,]
GO_BP_Cats_mRNA_AI_and_NB_and_NP$BY_FDR<-p.adjust(GO_BP_Cats_mRNA_AI_and_NB_and_NP$over_represented_pvalue, metho
d ="fdr")
GO_BP_Cats_mRNA_AI_and_NB_and_NP<-GO_BP_Cats_mRNA_AI_and_NB_and_NP[with(GO_BP_Cats_mRNA_AI_and_NB_and_NP, order(B
Y_FDR,over_represented_pvalue, -numDEInCat)), ]
#head(GO_BP_Cats_mRNA_AI_and_NB_and_NP, n=20)

GO_BP_Cats_mRNA_AI_and_NB_and_NP$fold_enrichment<-(GO_BP_Cats_mRNA_AI_and_NB_and_NP$numDEInCat/N_DEGs)/(GO_BP_Cat
s_mRNA_AI_and_NB_and_NP$numInCat/N_expressed_genes)
annotation.GO.BP.biomart_testgenes<-annotation.GO.BP.biomart[annotation.GO.BP.biomart$ensembl_gene_id %in% test.g
enes$a, ]
GO_BP_Cats_mRNA_AI_and_NB_and_NP<-merge(GO_BP_Cats_mRNA_AI_and_NB_and_NP,annotation.GO.BP.biomart_testgenes, by.x
="category", by.y="go_id", all.x=TRUE, all.y=FALSE)
GO_BP_Cats_mRNA_AI_and_NB_and_NP<-merge(GO_BP_Cats_mRNA_AI_and_NB_and_NP, annotation.ensembl.symbol, by.x="ensemb
l_gene_id", by.y="ensembl_gene_id", all=FALSE, all.x=TRUE, all.y=FALSE)
GO_BP_Cats_mRNA_AI_and_NB_and_NP<-GO_BP_Cats_mRNA_AI_and_NB_and_NP[with(GO_BP_Cats_mRNA_AI_and_NB_and_NP, order(B
Y_FDR,term)), ]
GO_BP_Cats_mRNA_AI_and_NB_and_NP<-GO_BP_Cats_mRNA_AI_and_NB_and_NP[GO_BP_Cats_mRNA_AI_and_NB_and_NP$BY_FDR<0.2,]
#write.table(GO_BP_Cats_mRNA_AI_and_NB_and_NP, paste(result_path, "2019_12_06_GO_BP_Cats_mRNA_diff_coexpression_i
nverted_AI_and_NB_and_NP.txt", sep="/"), quote=FALSE, sep="\t", row.names=FALSE)
```

```
knitr::kable(head(GO_BP_Cats_mRNA_AI_and_NB_and_NP), caption = "First five rows of Supplementary Table 5",format
 = 'pandoc')
```

First five rows of Supplementary Table 5

ensembl_gene_id	category	over_represented_pvalue	under_represented_pvalue	numDEInCat	numInCat	term	o

ensembl_gene_id	category	over_represented_pvalue	under_represented_pvalue	numDEInCat	numInCat	term	o
ENSBTAG00000000023	GO:0000122 (GO:0000122)	0.0204257	0.9950007	5	408	negative regulation of transcription by RNA polymerase II	B
ENSBTAG00000001163	GO:0000122 (GO:0000122)	0.0204257	0.9950007	5	408	negative regulation of transcription by RNA polymerase II	B
ENSBTAG00000005564	GO:0000122 (GO:0000122)	0.0204257	0.9950007	5	408	negative regulation of transcription by RNA polymerase II	B
ENSBTAG00000012178	GO:0000122 (GO:0000122)	0.0204257	0.9950007	5	408	negative regulation of transcription by RNA polymerase II	B
ENSBTAG00000021427	GO:0000122 (GO:0000122)	0.0204257	0.9950007	5	408	negative regulation of transcription by RNA polymerase II	B

Figure 1e

```
pearson_correlation_b<-pearson_correlation_a[pearson_correlation_a$gene_1 %in% GO_BP_Cats_mRNA_AI_and_NB_and_NP$e
nsembl_gene_id | pearson_correlation_a$gene_2 %in% GO_BP_Cats_mRNA_AI_and_NB_and_NP$ensembl_gene_id ,]

data_for_chart<-data.frame()
data_for_chart_a<-data.frame()
for(i in 1:dim(pearson_correlation_b)[1] ){
  expression_gene_1_AI<-rpkm_AIpreg[row.names(rpkm_AIpreg)==as.character(pearson_correlation_b$gene_1)[i],]
  expression_gene_2_AI<-rpkm_AIpreg[row.names(rpkm_AIpreg)==as.character(pearson_correlation_b$gene_2)[i],]
  expression_gene_1_NB<-rpkm_NBpreg[row.names(rpkm_NBpreg)==as.character(pearson_correlation_b$gene_1)[i],]
  expression_gene_2_NB<-rpkm_NBpreg[row.names(rpkm_NBpreg)==as.character(pearson_correlation_b$gene_2)[i],]
  expression_gene_1_NP<-rpkm_NOTpreg[row.names(rpkm_NOTpreg)==as.character(pearson_correlation_b$gene_1)[i],]
  expression_gene_2_NP<-rpkm_NOTpreg[row.names(rpkm_NOTpreg)==as.character(pearson_correlation_b$gene_2)[i],]

  symbol_gene_1<-annotation.ensembl.symbol[annotation.ensembl.symbol$ensembl_gene_id == as.character(pearson_corr
elation_b$gene_1)[i] , 2]
  symbol_gene_2<-annotation.ensembl.symbol[annotation.ensembl.symbol$ensembl_gene_id == as.character(pearson_corr
elation_b$gene_2)[i] , 2]
  chart<-i

  data_for_chart<-data.frame(gene_1=c(expression_gene_1_AI,expression_gene_1_NB,expression_gene_1_NP) ,
                             gene_2=c(expression_gene_2_AI,expression_gene_2_NB,expression_gene_2_NP),
                             symbol_gene_1,symbol_gene_2 ,chart,
                             group=rep(c("AI", "NB", "NP"), c(length(expression_gene_1_AI),length(expression_gene
_1_NB),length(expression_gene_1_NP))))

  data_for_chart_a<-rbind(data_for_chart_a,data_for_chart)
}

font_axis<-5
font_title<-8
ptsize<-0.05

plots<-list()
k<-1
for (j in c(1:4)){
  data_for_chart_b<-data_for_chart_a[data_for_chart_a$chart %in% j,]

plot<-ggplot(data_for_chart_b, aes(x=gene_1,y=gene_2))+
  geom_point()+
  geom_smooth(method=lm, size=ptsize, fill="lightgray")+
  scale_y_continuous(name=data_for_chart_b$symbol_gene_1[1])+
  scale_x_continuous(name=data_for_chart_b$symbol_gene_2[1])+
  facet_wrap(~group, ncol=3, scales="free")+
  theme(aspect.ratio = 1,
            panel.grid.major = element_blank(),
            panel.grid.minor = element_blank(),
            panel.background = element_blank(),
            plot.background  = element_blank(),
            axis.text = element_text( colour = 'black',size = font_axis),
            axis.title = element_text( colour = 'black' ,size = font_title, face="italic"),
            axis.ticks = element_line(size=0.1),
            panel.spacing.y = unit(0, "mm"),
            panel.spacing.x = unit(2, "mm"),
            legend.position="none",
            axis.line=element_line(size = 0.1, colour = "black"),
            strip.background=element_blank(),
            strip.text.x = element_blank())

 plots[[k]]<-plot
  k<-k+1
}

#png(filename =paste(result_path,"2019_11_14_plot_diff_cor_AI_NB_and_NP_inverted.png", sep="/"), width = 5.2, hei
ght =1.8, units = 'in', res = 300,bg = "transparent")
#ggarrange(plotlist=plots, ncol=2, nrow=2)
#dev.off()
ggarrange(plotlist=plots, ncol=2, nrow=2)
```

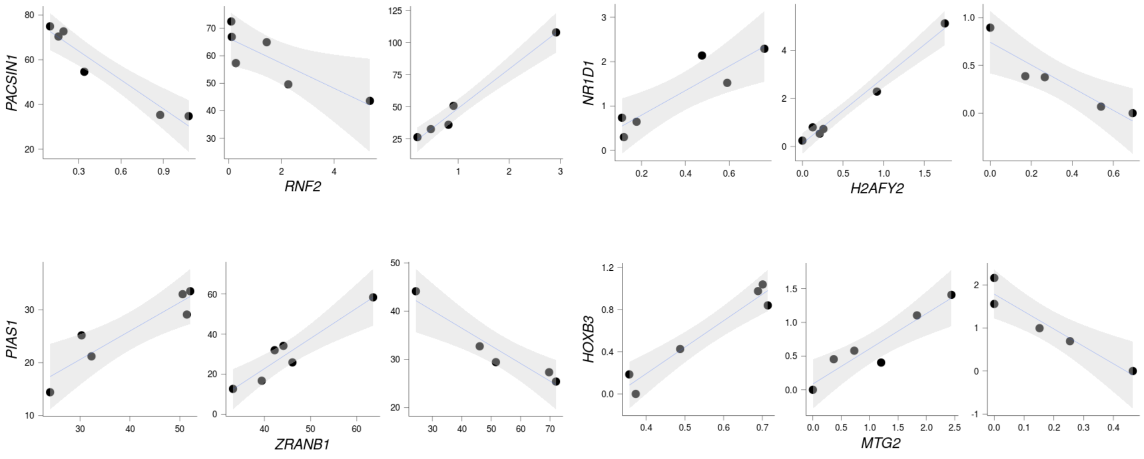


```
gc()
```

Differential miRNA:mRNA coexpression associated with pregnancy outcome

Supplementary table 6

```
correlation_all_animals_miRNA<-corAndPvalue( t(log2(rpkm_pwbc +1)), t(log2(rpkm_miRNA +1)) ,method="pearson", use
="pairwise.complete.obs")
correlation_all_animals_miRNA_a<-melt(correlation_all_animals_miRNA$cor)
correlation_all_animals_miRNA_a$pvalue<-melt(correlation_all_animals_miRNA$p)$value

correlation_all_animals_miRNA_a<-correlation_all_animals_miRNA_a[!duplicated(t(apply(correlation_all_animals_miRN
A_a[,c(1:2)], 1, sort))),]

correlation_all_animals_miRNA_b<-correlation_all_animals_miRNA_a

correlation_all_animals_miRNA_b$gene_1<-annotation.ensembl.symbol$external_gene_name[match( correlation_all_anima
ls_miRNA_b$Var1, annotation.ensembl.symbol$ensembl_gene_id )]
correlation_all_animals_miRNA_b$gene_2<-annotation.ensembl.symbol$external_gene_name[match( correlation_all_anima
ls_miRNA_b$Var2, annotation.ensembl.symbol$ensembl_gene_id )]
correlation_all_animals_miRNA_b$Var1<-as.character(correlation_all_animals_miRNA_b$Var1)
correlation_all_animals_miRNA_b$Var2<-as.character(correlation_all_animals_miRNA_b$Var2)

correlation_all_animals_miRNA_b$gene_1<-ifelse(correlation_all_animals_miRNA_b$gene_1=="", correlation_all_animal
s_miRNA_b$Var1,correlation_all_animals_miRNA_b$gene_1)
correlation_all_animals_miRNA_b$gene_2<-ifelse(correlation_all_animals_miRNA_b$gene_2=="", correlation_all_animal
s_miRNA_b$Var2,correlation_all_animals_miRNA_b$gene_2)

#write.table(correlation_all_animals_miRNA_b, file= paste(result_path,"2019_12_20_mRNA_miNRA_correlation_all_anim
als_miRNA.txt", sep="/"), append = FALSE, quote = FALSE, sep = "\t" ,row.names = FALSE)
#system("bzip2 -9 /mnt/storage/auburn/heifer_pregnancy/proj_2018/analysis/results/2019_12_20_mRNA_miNRA_correlati
on_all_animals_miRNA.txt")


stri1 <- function(x){
  ind <- stri_endswith_fixed(x, "-1")
  x[ind] <- stri_sub(x[ind],1, -3)
  x
}

stri2 <- function(x){
  ind <- stri_endswith_fixed(x, "-2")
  x[ind] <- stri_sub(x[ind],1, -3)
  x
}

stri3 <- function(x){
  ind <- stri_endswith_fixed(x, "-3")
  x[ind] <- stri_sub(x[ind],1, -3)
  x
}

stri4 <- function(x){
  ind <- stri_endswith_fixed(x, "-4")
  x[ind] <- stri_sub(x[ind],1, -3)
  x
}

stri5 <- function(x){
  ind <- stri_endswith_fixed(x, "-5")
  x[ind] <- stri_sub(x[ind],1, -3)
  x
}

correlation_all_animals_miRNA_b$gene_2<-stri1(correlation_all_animals_miRNA_b$gene_2)
correlation_all_animals_miRNA_b$gene_2<-stri2(correlation_all_animals_miRNA_b$gene_2)
correlation_all_animals_miRNA_b$gene_2<-stri3(correlation_all_animals_miRNA_b$gene_2)
correlation_all_animals_miRNA_b$gene_2<-stri4(correlation_all_animals_miRNA_b$gene_2)
correlation_all_animals_miRNA_b$gene_2<-stri5(correlation_all_animals_miRNA_b$gene_2)


correlation_all_animals_miRNA_b<-correlation_all_animals_miRNA_b[abs(correlation_all_animals_miRNA_b$value) > 0.8
5,]
#write.table(correlation_all_animals_miRNA_b, file= paste(result_path,"2020_01_29_mRNA_miNRA_sig_correlation_all_
animals_miRNA.txt", sep="/"), append = FALSE, quote = FALSE, sep = "\t" ,row.names = FALSE)
```

```
knitr::kable(head(correlation_all_animals_miRNA_b), caption = "First five rows of Supplementary Table 6",format =
'pandoc')
```

First five rows of Supplementary Table 6

	Var1	Var2	value	pvalue	gene_1	gene_2
1695	ENSBTAG00000004457	ENSBTAG00000029760	-0.8568286	1.12e-05	ORAI1	bta-mir-320a

	Var1	Var2	value	pvalue	gene_1	gene_2
3125	ENSBTAG00000008309	ENSBTAG00000029760	-0.8552897	1.21e-05	ATP13A2	bta-mir-320a
4135	ENSBTAG00000011064	ENSBTAG00000029760	-0.8519537	1.42e-05	ADCK5	bta-mir-320a
4551	ENSBTAG00000012172	ENSBTAG00000029760	-0.8553540	1.21e-05	MIDN	bta-mir-320a
10155	ENSBTAG00000051917	ENSBTAG00000029760	-0.8514464	1.45e-05	ZNF579	bta-mir-320a
12191	ENSBTAG00000004457	ENSBTAG00000029761	-0.8749960	4.30e-06	ORAI1	bta-mir-320a

Supplementary Fig. 2

Calculate the empirical false discovery rate for Pearson’s co-expression and differential coexpression

```
permutation_matrix<-matrix(,nrow=200100,ncol=17)
#head(permutation_matrix)
#dim(permutation_matrix)

for (i in seq(1:200100)){
  sampling <- sample(1:17,17, replace = FALSE)
  if ( ! identical(sampling , c(1:17))){
    permutation_matrix[i,]<-sample(1:17,17, replace = FALSE)
  }}

permutation_matrix<-permutation_matrix[!duplicated(permutation_matrix),]
dim(permutation_matrix)
head(permutation_matrix)

permutation_matrix<-permutation_matrix[sample(1:dim(permutation_matrix)[1], 5000),]
rand<-dim(permutation_matrix)[1]

coexp<-seq(0.8, 1, 0.01)

results <- filebacked.big.matrix(length(coexp),rand, type="double", init=0, separated=FALSE,
                                 backingfile="incidence_matrix.bin",
                                 descriptor="incidence_matrix.desc")
mdesc_result<- describe(results)

cl <- makeCluster(20)
registerDoParallel(cl)

results[,]<-foreach(i = coexp, .combine='rbind',.packages=c("reshape2","bigmemory"), .inorder=TRUE, .verbose=TRUE
) %:%
            foreach(j = 1:rand, .combine='cbind',.packages=c("reshape2","bigmemory"), .inorder=FALSE, .verbose=TR
UE ) %dopar% {

   rpkm_pwbc_scramble<-log2(rpkm_pwbc[,permutation_matrix[j,]] +1)
   cor_1<-cor( t(rpkm_pwbc_scramble), t(log2(rpkm_miRNA +1)) ,method="pearson", use="pairwise.complete.obs")
   length(which(abs(cor_1) > i))
  }
stopCluster(cl)

total.rand <-  5000 * 10496 * 290

qvalue<-data.frame(correlation = coexp,
                   e.pvalue= (rowSums(results[,]+1))/(total.rand+1),
                   e.pvalue.round= round((rowSums(results[,]+1))/(total.rand+1) ,6))

qvalue


write.table(qvalue, file= paste(result_path,"2019_12_09_mRNA_miNRA_coexpression_empiricalFDR.txt", sep="/"), appe
nd = FALSE, quote = FALSE, sep = "\t" ,row.names = FALSE)


rm(results)
system("rm incidence_matrix.bin")
system("rm incidence_matrix.desc")
rm(permutation_matrix)
```

```
eFDR_coexpression<-read.delim(paste(result_path,"2019_12_09_mRNA_miNRA_coexpression_empiricalFDR.txt", sep="/"),
 row.names=NULL,header =TRUE, stringsAsFactors =FALSE)
```

```
font_size<-12 #font_size 12 for pdf  figure

scaleFUN <- function(x) sprintf("%.2f", x)

plot1<-ggplot()+
  geom_point(data=eFDR_coexpression, aes(x=correlation , y=e.pvalue.round),color="black", size=3, shape=16)+
  geom_line(data=eFDR_coexpression, aes(x=correlation , y=e.pvalue.round),color="black", size=0.1,linetype=3)+
  scale_y_continuous(name="empirical FDR", limits = c(0, 0.0002), breaks=seq(0,0.0002, 0.00001))+
  scale_x_continuous(name="correlation (r)", limits = c(0.8, 1), breaks=seq(0.8,1, 0.01), labels=scaleFUN)+
  ggtitle("mRNA and miRNA coexpression all heifers")+
  theme_bw()+
  theme(panel.grid= element_blank(),
        panel.background = element_blank(),
        panel.grid.minor = element_blank(),
        panel.grid.major = element_line(color="lightgray"),
        plot.background = element_blank(),
        axis.title=element_text(color="black", size=font_size),
        axis.text.y=element_text(color="black", size=font_size),
        axis.text.x=element_text(color="black", size=font_size, angle=90),
        panel.spacing = unit(c(0.4,0.4,0.4,0.4),"cm"),
        plot.margin = unit(c(0.5,0.5,0.5,0.5),"cm"),
        legend.position="none",
        plot.title = element_text(lineheight=.8, hjust=0.5, size= font_size))


#font_size 12 for figure
#pdf(paste(result_path,"2019_12_11_mRNA_miRNA_eFDR_plots.pdf", sep="/"), width=9, height=5)
#plot1
#dev.off()

plot1
```

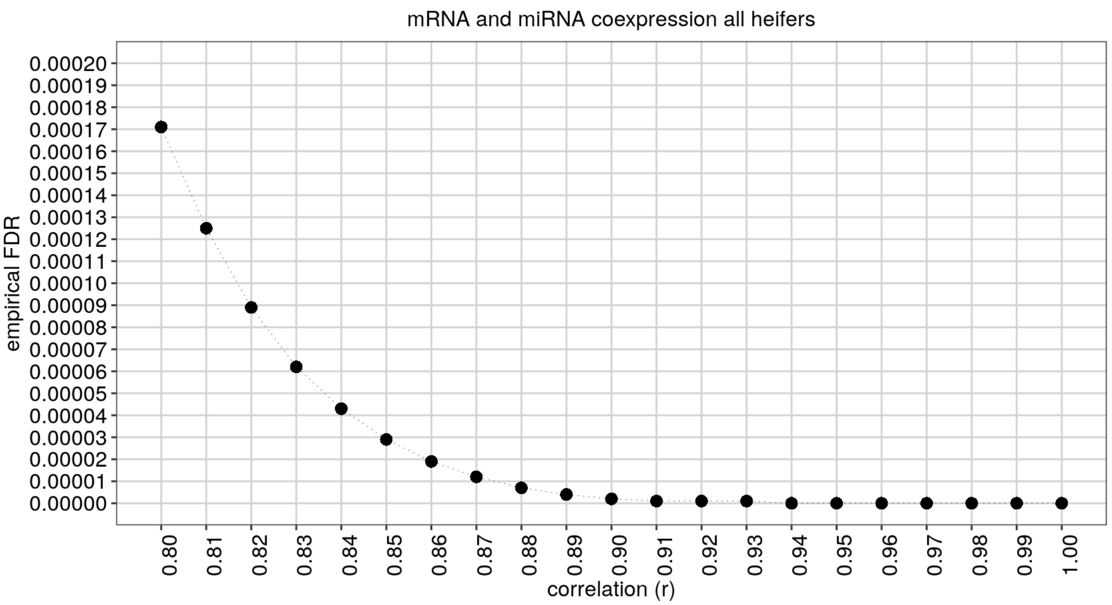

Supplementary Table 7

```
test.genes<-data.frame(a=unique(as.character(correlation_all_animals_miRNA_b$Var1)), stringsAsFactors=FALSE)
all_genes_numeric<-as.integer(all_genes$gene %in%test.genes$a)
names(all_genes_numeric)<-all_genes$gene

N_DEGs<-length(test.genes$a)

set.seed(54345)
pwf<-nullp(all_genes_numeric, bias.data=annotation.genelength.biomart_vector, plot.fit=FALSE )
GO_BP_Cats_mRNA_miRNA_all_animals<-goseq(pwf,gene2cat=annotation.GO.BP.biomart, method ="Sampling", repcnt = 7000
, use_genes_without_cat=FALSE)
GO_BP_Cats_mRNA_miRNA_all_animals<-GO_BP_Cats_mRNA_miRNA_all_animals[GO_BP_Cats_mRNA_miRNA_all_animals$numDEInCat
>=4,]
GO_BP_Cats_mRNA_miRNA_all_animals$BY_FDR<-p.adjust(GO_BP_Cats_mRNA_miRNA_all_animals$over_represented_pvalue, met
hod ="fdr")
GO_BP_Cats_mRNA_miRNA_all_animals<-GO_BP_Cats_mRNA_miRNA_all_animals[with(GO_BP_Cats_mRNA_miRNA_all_animals, orde
r(BY_FDR,over_represented_pvalue, -numDEInCat)), ]
#head(GO_BP_Cats_mRNA_miRNA_all_animals, n=20)

GO_BP_Cats_mRNA_miRNA_all_animals$fold_enrichment<-(GO_BP_Cats_mRNA_miRNA_all_animals$numDEInCat/N_DEGs)/(GO_BP_C
ats_mRNA_miRNA_all_animals$numInCat/N_expressed_genes)
annotation.GO.BP.biomart_testgenes<-annotation.GO.BP.biomart[annotation.GO.BP.biomart$ensembl_gene_id %in% test.g
enes$a, ]
GO_BP_Cats_mRNA_miRNA_all_animals<-merge(GO_BP_Cats_mRNA_miRNA_all_animals,annotation.GO.BP.biomart_testgenes, b
y.x="category", by.y="go_id", all.x=TRUE, all.y=FALSE)
GO_BP_Cats_mRNA_miRNA_all_animals<-merge(GO_BP_Cats_mRNA_miRNA_all_animals, annotation.ensembl.symbol, by.x="ense
mbl_gene_id", by.y="ensembl_gene_id", all=FALSE, all.x=TRUE, all.y=FALSE)
GO_BP_Cats_mRNA_miRNA_all_animals<-GO_BP_Cats_mRNA_miRNA_all_animals[with(GO_BP_Cats_mRNA_miRNA_all_animals, orde
r(BY_FDR,term)), ]
GO_BP_Cats_mRNA_miRNA_all_animals<-GO_BP_Cats_mRNA_miRNA_all_animals[GO_BP_Cats_mRNA_miRNA_all_animals$BY_FDR<0.1
,]
#write.table(GO_BP_Cats_mRNA_miRNA_all_animals, paste(result_path, "2020_01_29_GO_BP_Cats_mRNA_miRNA_all_animals.
txt", sep="/"), quote=FALSE, sep="\t", row.names=FALSE)
```

```
knitr::kable(head(GO_BP_Cats_mRNA_miRNA_all_animals), caption = "First five rows of Supplementary Table 7",format
= 'pandoc')
```

First five rows of Supplementary Table 7

	ensembl_gene_id	category	over_represented_pvalue	under_represented_pvalue	numDEInCat	numInCat	term
17	ENSBTAG00000005408	GO:0018107 (GO:0018107)	0.0004285	1.0000000	4	42	peptidyl-threonine phosphoryl
29	ENSBTAG00000017401	GO:0018107 (GO:0018107)	0.0004285	1.0000000	4	42	peptidyl-threonine phosphoryl
48	ENSBTAG00000033333	GO:0018107 (GO:0018107)	0.0004285	1.0000000	4	42	peptidyl-threonine phosphoryl
49	ENSBTAG00000033801	GO:0018107 (GO:0018107)	0.0004285	1.0000000	4	42	peptidyl-threonine phosphoryl
35	ENSBTAG00000019915	GO:0060271 (GO:0060271)	0.0164262	0.9972861	4	106	cilium asse
46	ENSBTAG00000030175	GO:0060271 (GO:0060271)	0.0164262	0.9972861	4	106	cilium asse

Figure 3a

```
correlation_all_animals_miRNA_c<-correlation_all_animals_miRNA_b[,c("gene_2", "gene_1", "value")]
correlation_all_animals_miRNA_c<-correlation_all_animals_miRNA_c[correlation_all_animals_miRNA_c$gene_1 %in% GO_B
P_Cats_mRNA_miRNA_all_animals$external_gene_name,]
colnames(correlation_all_animals_miRNA_c)<- c("source", "target", "value")

correlation_all_animals_miRNA_c<-rbind(correlation_all_animals_miRNA_c, data.frame(source=GO_BP_Cats_mRNA_miRNA_a
ll_animals[,c("external_gene_name")],target=GO_BP_Cats_mRNA_miRNA_all_animals[,c("term")], value=1))

ids <- unique(as.character(c(correlation_all_animals_miRNA_c$source, correlation_all_animals_miRNA_c$target)))
nodes <- data.frame(node = c(0:23),
                    name = ids)

results <- merge(correlation_all_animals_miRNA_c, nodes, by.x = "source", by.y = "name")
results <- merge(results, nodes, by.x = "target", by.y = "name")
links <- results[ , c("node.x", "node.y", "value")]
colnames(links) <- c("source", "target", "value")

links$value<-1

networkD3::sankeyNetwork(Links = links, Nodes = nodes, Source = 'source',
                         Target = 'target',  NodeID = 'name', Value = "value",
                         fontSize = 15,
                        nodeWidth = 5,
                        nodePadding = 5
                        )
```

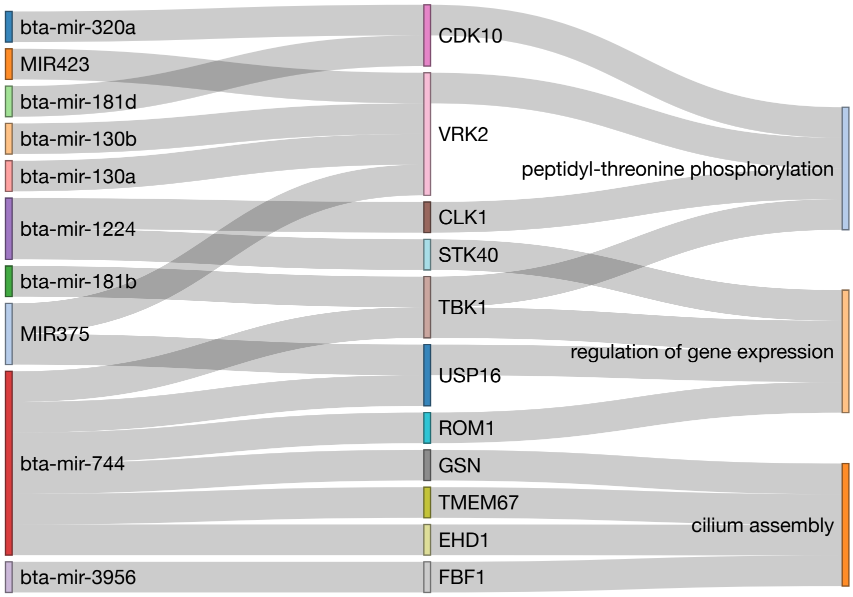

```
correlation_all_animals_miRNA_c<-correlation_all_animals_miRNA_b[correlation_all_animals_miRNA_b$Var1 %in% GO_BP_
Cats_mRNA_miRNA_all_animals$ensembl_gene_id[GO_BP_Cats_mRNA_miRNA_all_animals$term=='regulation of gene expressio
n'],]

data_for_chart<-data.frame()
data_for_chart_a<-data.frame()
for(i in 1:dim(correlation_all_animals_miRNA_c)[1]){
  expression_mRNA<-cpm_pwbc[as.character(correlation_all_animals_miRNA_c[i,1]),]
  expresson_miRNA<-cpm_miRNA[as.character(correlation_all_animals_miRNA_c[i,2]),]
  symbol_mRNA<-correlation_all_animals_miRNA_c[i,5]
  symbol_miRNA<-correlation_all_animals_miRNA_c[i,6]
  chart<-i
  data_for_chart<-data.frame(symbol_mRNA,symbol_miRNA,expression_mRNA,expresson_miRNA,chart)
  data_for_chart_a<-rbind(data_for_chart_a,data_for_chart)
}

data_for_chart_a[is.na(data_for_chart_a)]<-0

plots<-list()
k<-1
for (j in c(1:6)){
data_for_chart_b<-data_for_chart_a[data_for_chart_a$chart %in% j,]
plot<-ggplot(data_for_chart_b, aes(x=log(expresson_miRNA+1),y=log(expression_mRNA+1)))+
  geom_point()+
  geom_smooth(method=lm, size=0.1, fill="lightgray")+
  scale_y_continuous(name=data_for_chart_b$symbol_mRNA[1])+
  scale_x_continuous(name=data_for_chart_b$symbol_miRNA[1])+
  theme(aspect.ratio = 1,
            panel.grid.major = element_blank(),
            panel.grid.minor = element_blank(),
            panel.background = element_blank(),
            plot.background  = element_blank(),
            axis.text.x = element_text( colour = 'black' ,size = 7, angle=90),
            axis.text.y = element_text( colour = 'black',size = 7),
            axis.title= element_text( colour = 'black' ,size = 7, face="italic"),
            axis.ticks = element_line(size=0.1),
            panel.spacing = unit(0, "mm"),
            plot.margin = ggplot2::margin(1,1,1,1,unit= "mm"),
            legend.position="none",
            axis.line=element_line(size = 0.1, colour = "black"))

plots[[k]]<-plot
k<-k+1
}
#png(filename =paste(result_path,"2019_12_20_plot_mrna_mirna_all_samples_regulation_gene_expression.png", sep
="/"), width = 3, height = 2, units = 'in', res = 300)
#ggarrange(plotlist=plots, ncol=3, nrow=2)
#dev.off()
ggarrange(plotlist=plots, ncol=3, nrow=2)
```

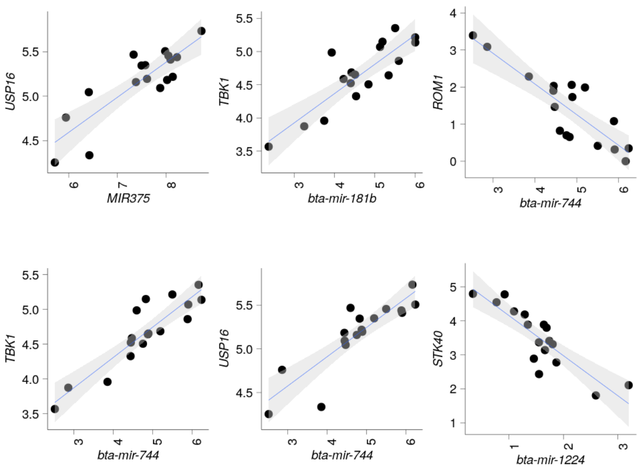

```
correlation_all_animals_miRNA_c<-correlation_all_animals_miRNA_b[correlation_all_animals_miRNA_b$Var1 %in% GO_BP_
Cats_mRNA_miRNA_all_animals$ensembl_gene_id[GO_BP_Cats_mRNA_miRNA_all_animals$term=='peptidyl-threonine phosphory
lation'],]

data_for_chart<-data.frame()
data_for_chart_a<-data.frame()
for(i in 1:dim(correlation_all_animals_miRNA_c)[1]){
  expression_mRNA<-cpm_pwbc[as.character(correlation_all_animals_miRNA_c[i,1]),]
  expresson_miRNA<-cpm_miRNA[as.character(correlation_all_animals_miRNA_c[i,2]),]
  symbol_mRNA<-correlation_all_animals_miRNA_c[i,5]
  symbol_miRNA<-correlation_all_animals_miRNA_c[i,6]
  chart<-i
  data_for_chart<-data.frame(symbol_mRNA,symbol_miRNA,expression_mRNA,expresson_miRNA,chart)
  data_for_chart_a<-rbind(data_for_chart_a,data_for_chart)
}

data_for_chart_a[is.na(data_for_chart_a)]<-0

plots<-list()
k<-1
for (j in c(1:9)){
data_for_chart_b<-data_for_chart_a[data_for_chart_a$chart %in% j,]
plot<-ggplot(data_for_chart_b, aes(x=log(expresson_miRNA+1),y=log(expression_mRNA+1)))+
  geom_point()+
  geom_smooth(method=lm, size=0.1, fill="lightgray")+
  scale_y_continuous(name=data_for_chart_b$symbol_mRNA[1])+
  scale_x_continuous(name=data_for_chart_b$symbol_miRNA[1])+
  theme(aspect.ratio = 1,
            panel.grid.major = element_blank(),
            panel.grid.minor = element_blank(),
            panel.background = element_blank(),
            plot.background  = element_blank(),
            axis.text.x = element_text( colour = 'black' ,size = 7, angle=90),
            axis.text.y = element_text( colour = 'black',size = 7),
            axis.title= element_text( colour = 'black' ,size = 7, face="italic"),
            axis.ticks = element_line(size=0.1),
            panel.spacing = unit(0, "mm"),
            plot.margin = ggplot2::margin(1,1,1,1,unit= "mm"),
            legend.position="none",
            axis.line=element_line(size = 0.1, colour = "black"))

plots[[k]]<-plot
k<-k+1
}
#png(filename =paste(result_path,"2019_12_20_plot_mrna_mirna_all_samples_peptidyl_threonine_phosphorylation.png",
sep="/"), width = 3, height = 3, units = 'in', res = 300)
#ggarrange(plotlist=plots, ncol=3, nrow=3)
#dev.off()
ggarrange(plotlist=plots, ncol=3, nrow=3)
```

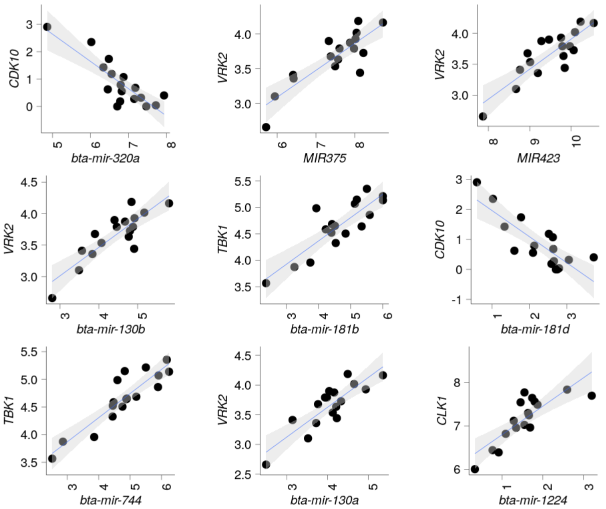

Figure 3b

```
bta_miRWalk_3UTR$type<-"3UTR"
bta_miRWalk_5UTR$type<-"5UTR"
bta_miRWalk_CDS$type<-"CDS"

combined_bta_miRWalk<-rbind(bta_miRWalk_3UTR,rbind(bta_miRWalk_5UTR,bta_miRWalk_CDS))

combined_bta_miRWalk$miRNA_lower_case<-tolower(combined_bta_miRWalk$miRNA)
correlation_all_animals_miRNA_b$gene_2_lower_case<-tolower(correlation_all_animals_miRNA_b$gene_2)
correlation_all_animals_miRNA_c<-merge(correlation_all_animals_miRNA_b, combined_bta_miRWalk, by.x=c("gene_2_lowe
r_case", "gene_1" ), by.y=c("miRNA_lower_case", "Genesymbol" ), all=FALSE)
correlation_all_animals_miRNA_c<-correlation_all_animals_miRNA_c[!duplicated(correlation_all_animals_miRNA_c[,c(
"gene_2_lower_case", "gene_1")]),]
correlation_all_animals_miRNA_c<-correlation_all_animals_miRNA_c[correlation_all_animals_miRNA_c$value < -0.85,]
correlation_all_animals_miRNA_c<-correlation_all_animals_miRNA_c[order(correlation_all_animals_miRNA_c$gene_2_low
er_case, correlation_all_animals_miRNA_c$gene_1),]

#write.table(correlation_all_animals_miRNA_c, file= paste(result_path,"2019_12_24_mRNA_miNRA_correlation_all_anim
als_mirwalk.txt", sep="/"), append = FALSE, quote = FALSE, sep = "\t" ,row.names = FALSE)
#system("bzip2 -9 /mnt/storage/auburn/heifer_pregnancy/proj_2018/analysis/results/2019_12_24_mRNA_miNRA_correlati
on_all_animals_mirwalk.txt")


data_for_chart<-data.frame()
data_for_chart_a<-data.frame()
for(i in 1:dim(correlation_all_animals_miRNA_c)[1]){
  expression_mRNA<-cpm_pwbc[as.character(correlation_all_animals_miRNA_c[i,3]),]
  expresson_miRNA<-cpm_miRNA[as.character(correlation_all_animals_miRNA_c[i,4]),]
  symbol_mRNA<-correlation_all_animals_miRNA_c[i,2]
  symbol_miRNA<-correlation_all_animals_miRNA_c[i,1]
  chart<-i
  data_for_chart<-data.frame(symbol_mRNA,symbol_miRNA,expression_mRNA,expresson_miRNA,chart)
  data_for_chart_a<-rbind(data_for_chart_a,data_for_chart)
}

data_for_chart_a[is.na(data_for_chart_a)]<-0

plots<-list()
k<-1
for (j in c(1:30)){
data_for_chart_b<-data_for_chart_a[data_for_chart_a$chart %in% j,]
plot<-ggplot(data_for_chart_b, aes(x=log(expresson_miRNA+1),y=log(expression_mRNA+1)))+
  geom_point()+
  geom_smooth(method=lm, size=0.1, fill="lightgray")+
  scale_y_continuous(name=data_for_chart_b$symbol_mRNA[1])+
  scale_x_continuous(name=data_for_chart_b$symbol_miRNA[1])+
  theme(aspect.ratio = 1,
            panel.grid.major = element_blank(),
            panel.grid.minor = element_blank(),
            panel.background = element_blank(),
            plot.background  = element_blank(),
            axis.text.x = element_text( colour = 'black' ,size = 7, angle=90),
            axis.text.y = element_text( colour = 'black',size = 7),
            axis.title= element_text( colour = 'black' ,size = 7, face="italic"),
            axis.ticks = element_line(size=0.1),
            panel.spacing = unit(0, "mm"),
            plot.margin = ggplot2::margin(1,1,1,1,unit= "mm"),
            legend.position="none",
            axis.line=element_line(size = 0.1, colour = "black"))

plots[[k]]<-plot
k<-k+1
}
#png(filename =paste(result_path,"2019_12_21_plot_mrna_mirna_all_samples_binding_sites.png", sep="/"), width = 1
2, height = 3, units = 'in', res = 300)
#ggarrange(plotlist=plots, ncol=10, nrow=3)
#dev.off()
ggarrange(plotlist=plots, ncol=10, nrow=3)
```

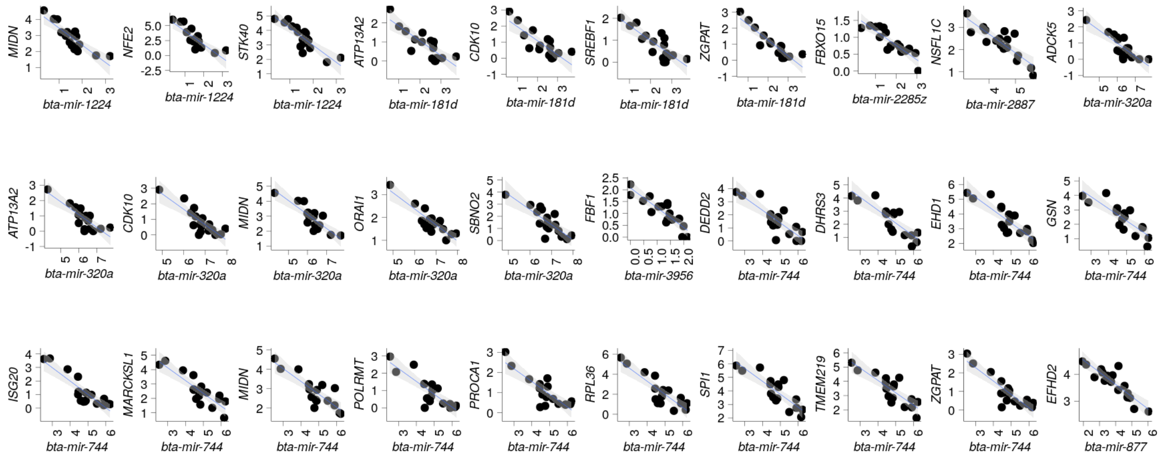

Create correlation matrices for AI pregnant NB pregnant and non pregnant animals.

```
rpkm_AIpreg_a<-t(log2(rpkm_AIpreg + 1))
rpkm_AIpreg_miRNA_a<-t(log2(rpkm_AIpreg_miRNA + 1))

rpkm_NBpreg_a<-t(log2(rpkm_NBpreg + 1))
rpkm_NBpreg_miRNA_a<-t(log2(rpkm_NBpreg_miRNA + 1))

rpkm_NOTpreg_a<-t(log2(rpkm_NOTpreg + 1))
rpkm_NOTpreg_miRNA_a<-t(log2(rpkm_NOTpreg_miRNA + 1))


correlation_AIpreg_mRNA_miRNA<-corAndPvalue(rpkm_AIpreg_a, rpkm_AIpreg_miRNA_a,method="pearson", use="pairwise.co
mplete.obs")
correlation_AIpreg_mRNA_miRNAa<-melt(correlation_AIpreg_mRNA_miRNA$cor)
correlation_AIpreg_mRNA_miRNAa$pvalue<-melt(correlation_AIpreg_mRNA_miRNA$p)$value
rm(correlation_AIpreg_mRNA_miRNA)
dim(correlation_AIpreg_mRNA_miRNAa)#3043840
```

```
## [1] 3043840       4
```

```
correlation_NBpreg_mRNA_miRNA<-corAndPvalue(rpkm_NBpreg_a, rpkm_NBpreg_miRNA_a,method="pearson", use="pairwise.co
mplete.obs")
correlation_NBpreg_mRNA_miRNAa<-melt(correlation_NBpreg_mRNA_miRNA$cor)
correlation_NBpreg_mRNA_miRNAa$pvalue<-melt(correlation_NBpreg_mRNA_miRNA$p)$value
rm(correlation_NBpreg_mRNA_miRNA)
dim(correlation_NBpreg_mRNA_miRNAa)#3043840
```

```
## [1] 3043840       4
```

```
correlation_NOTpreg_mRNA_miRNA<-corAndPvalue(rpkm_NOTpreg_a, rpkm_NOTpreg_miRNA_a,method="pearson", use="pairwis
e.complete.obs")
correlation_NOTpreg_mRNA_miRNAa<-melt(correlation_NOTpreg_mRNA_miRNA$cor)
correlation_NOTpreg_mRNA_miRNAa$pvalue<-melt(correlation_NOTpreg_mRNA_miRNA$p)$value
rm(correlation_NOTpreg_mRNA_miRNA)
dim(correlation_NOTpreg_mRNA_miRNAa) #3043840
```

```
## [1] 3043840       4
```

```
merged_correlation_mRNA_miRNA<-cbind(correlation_AIpreg_mRNA_miRNAa,cbind(correlation_NBpreg_mRNA_miRNAa,correlat
ion_NOTpreg_mRNA_miRNAa))
merged_correlation_mRNA_miRNA<-merged_correlation_mRNA_miRNA[,c(1,2,3,4,7,8,11,12)]
colnames(merged_correlation_mRNA_miRNA)<-c("mRNA_gene","miRNA_gene","cor_AI","pvalue_AI","cor_NB","pvalue_NB","co
r_NP","pvalue_NP")

merged_correlation_mRNA_miRNA$mRNA_symbol<-annotation.ensembl.symbol$external_gene_name[match( merged_correlation
_mRNA_miRNA$mRNA_gene, annotation.ensembl.symbol$ensembl_gene_id )]
merged_correlation_mRNA_miRNA$miRNA_symbol<-annotation.ensembl.symbol$external_gene_name[match( merged_correlatio
n_mRNA_miRNA$miRNA_gene, annotation.ensembl.symbol$ensembl_gene_id )]

merged_correlation_mRNA_miRNA$subtraction_cor_AI_cor_NP<-merged_correlation_mRNA_miRNA$cor_AI - merged_correlatio
n_mRNA_miRNA$cor_NP
merged_correlation_mRNA_miRNA$subtraction_cor_NB_cor_NP<-merged_correlation_mRNA_miRNA$cor_NB - merged_correlatio
n_mRNA_miRNA$cor_NP
merged_correlation_mRNA_miRNA$subtraction_cor_AI_cor_NB<-merged_correlation_mRNA_miRNA$cor_AI - merged_correlatio
n_mRNA_miRNA$cor_NB
```

Supplementary Fig. 3

```
setwd("/mnt/storage/auburn/heifer_pregnancy/proj_2018/analysis/results")

permutation_matrix<-matrix(,nrow=200100,ncol=17)
#head(permutation_matrix)
#dim(permutation_matrix)

for (i in seq(1:200100)){
  sampling <- sample(1:17,17, replace = FALSE)
  if ( ! identical(sampling , c(1:17))){
    permutation_matrix[i,]<-sample(1:17,17, replace = FALSE)
  }}

permutation_matrix<-permutation_matrix[!duplicated(permutation_matrix),]
dim(permutation_matrix)
head(permutation_matrix)

permutation_matrix<-permutation_matrix[sample(1:dim(permutation_matrix)[1], 5000),]
rand<-dim(permutation_matrix)[1]

sequence.pvalue<-seq(0.7, 1, 0.01)

results <- filebacked.big.matrix(length(sequence.pvalue),rand, type="double", init=0, separated=FALSE,
                                 backingfile="incidence_matrix.bin",
                                 descriptor="incidence_matrix.desc")  #this does not overwrite, create once and d
elete if want to create again
mdesc_result<- describe(results)

cl <- makeCluster(20)
registerDoParallel(cl)

results[,]<-foreach(i = sequence.pvalue, .combine='rbind', .inorder=TRUE,  .packages=c("bigmemory"), .verbose=FAL
SE) %:%
  foreach(j = 1:rand, .combine='cbind', .inorder=FALSE,.packages=c("reshape2","bigmemory"), .verbose=FALSE ) %dop
ar% {

   rpkm_pwbc_scramble<-log2(rpkm_pwbc[,permutation_matrix[j,]] +1)
   cor_1<-cor( t(rpkm_pwbc_scramble[,sample(17,6)]), t(log2(rpkm_miRNA +1)[,sample(17,6)]) ,method="pearson", use
="pairwise.complete.obs")
   length(which(abs(cor_1) > i))
  }

stopCluster(cl)

results[1:5,1:5]


total.rand <- rand * 10496 * 290

qvalue<-data.frame(correlation = sequence.pvalue,
                   e.pvalue= (rowSums(results[,]+1))/(total.rand+1),
                   e.pvalue.round= round((rowSums(results[,]+1))/(total.rand+1) ,6))

write.table(qvalue, file= paste(result_path,"/2019_12_23_mRNA_miRNA_within_group_coexpression_empiricalFDR.txt",
 sep=""), append = FALSE, quote = FALSE, sep = "\t" ,row.names = FALSE)

qvalue_two_tailled<-data.frame(correlation = sequence.pvalue,
                   e.pvalue= ( (rowSums(results[,]+1))/(total.rand+1) )/2  ,
                   e.pvalue.round= ( round((rowSums(results[,]+1))/(total.rand+1) ,6) )/2 )

write.table(qvalue, file= paste(result_path,"/2019_12_23_mRNA_miRNA_within_group_coexpression_empiricalFDR_twotai
l.txt", sep=""), append = FALSE, quote = FALSE, sep = "\t" ,row.names = FALSE)


rm(results)
system("rm incidence_matrix.bin")
system("rm incidence_matrix.desc")
```

```
eFDR_coexpression<-read.delim(paste(result_path,"2019_12_23_mRNA_miRNA_within_group_coexpression_empiricalFDR_two
tail.txt", sep="/"), row.names=NULL,header =TRUE, stringsAsFactors =FALSE)
```

```
font_size<-12 #font_size 12 for pdf  figure

scaleFUN <- function(x) sprintf("%.2f", x)

plot1<-ggplot()+
  geom_point(data=eFDR_coexpression, aes(x=correlation , y=e.pvalue.round),color="black", size=3, shape=16)+
  geom_line(data=eFDR_coexpression, aes(x=correlation , y=e.pvalue.round),color="black", size=0.1,linetype=3)+
  scale_y_continuous(name="empirical FDR", limits = c(0, 0.15), breaks=seq(0,0.2, 0.01))+
  scale_x_continuous(name="correlation (r)", limits = c(0.7, 1), breaks=seq(0.7,1, 0.01), labels=scaleFUN)+
  ggtitle("mRNA and miRNA coexpression within a group of heifers")+
  theme_bw()+
  theme(panel.grid= element_blank(),
        panel.background = element_blank(),
        panel.grid.minor = element_blank(),
        panel.grid.major = element_line(color="lightgray"),
        plot.background = element_blank(),
        axis.title=element_text(color="black", size=font_size),
        axis.text.y=element_text(color="black", size=font_size),
        axis.text.x=element_text(color="black", size=font_size, angle=90),
        panel.spacing = unit(c(0.4,0.4,0.4,0.4),"cm"),
        plot.margin = unit(c(0.5,0.5,0.5,0.5),"cm"),
        legend.position="none",
        plot.title = element_text(lineheight=.8, hjust=0.5, size= font_size))


#font_size 12 for figure
#pdf(paste(result_path,"2020_01_29_mRNA_miRNA_within_group_eFDR_plots.pdf", sep="/"), width=9, height=5)
#plot1
#dev.off()
plot1
```

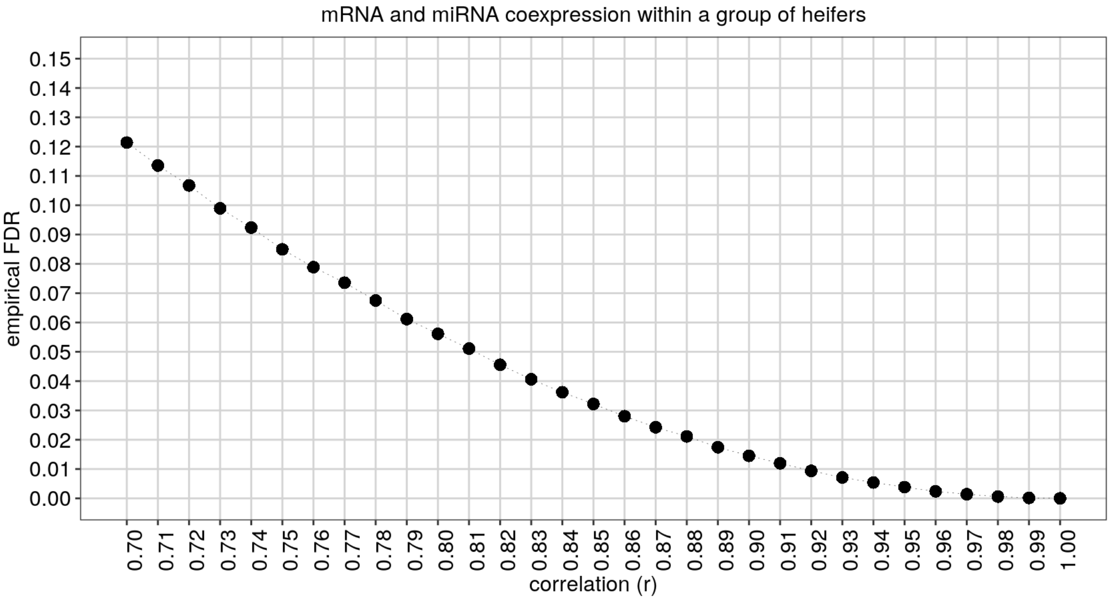

Supplementary Table 8

```
merged_correlation_mRNA_miRNA$result_cor_AI_and_NB_cor_NP_a<-ifelse( (abs(merged_correlation_mRNA_miRNA$subtracti
on_cor_AI_cor_NP) + abs(merged_correlation_mRNA_miRNA$subtraction_cor_NB_cor_NP)) / 2 > 1.8 , "inverted",
                                                                    ifelse( (merged_correlation_mRNA_miRNA$cor_A
I >0.90  & merged_correlation_mRNA_miRNA$cor_NB >0.90)  & (merged_correlation_mRNA_miRNA$cor_NP > -0.1 & merged_c
orrelation_mRNA_miRNA$cor_NP < 0.1) , "loss_positive",
                                   ifelse( (merged_correlation_mRNA_miRNA$cor_AI < -0.90  & merged_correlation_mR
NA_miRNA$cor_NB < -0.90)  & (merged_correlation_mRNA_miRNA$cor_NP > -0.1 & merged_correlation_mRNA_miRNA$cor_NP <
0.1) , "loss_negative",
                                   ifelse( (merged_correlation_mRNA_miRNA$cor_AI > -0.1 & merged_correlation_mRNA
_miRNA$cor_AI < 0.1 & merged_correlation_mRNA_miRNA$cor_NB > -0.1 & merged_correlation_mRNA_miRNA$cor_NB < 0.1 )
&  merged_correlation_mRNA_miRNA$cor_NP > 0.90, "gain_positive" ,
                                   ifelse( (merged_correlation_mRNA_miRNA$cor_AI > -0.1 & merged_correlation_mRNA
_miRNA$cor_AI < 0.1 & merged_correlation_mRNA_miRNA$cor_NB > -0.1 & merged_correlation_mRNA_miRNA$cor_NB < 0.1 )
&  merged_correlation_mRNA_miRNA$cor_NP < -0.90, "gain_negative" ,"nothing")))))

table(merged_correlation_mRNA_miRNA$result_cor_AI_and_NB_cor_NP_a)
```

```
##
## gain_negative gain_positive      inverted loss_negative loss_positive
##          2330          2738            51             2             3
##       nothing
##       3038716
```

```
#head(merged_correlation_mRNA_miRNA)

merged_correlation_mRNA_miRNA<-merged_correlation_mRNA_miRNA[!(merged_correlation_mRNA_miRNA$result_cor_AI_and_NB
_cor_NP_a== "nothing"),]

#write.table(merged_correlation_mRNA_miRNA, file= paste(result_path,"/2020_01_29_mRNA_miRNA_sig_differential_coex
pression.txt", sep=""), append = FALSE, quote = FALSE, sep = "\t" ,row.names = FALSE)


merged_correlation_mRNA_miRNA_gain_positive<-merged_correlation_mRNA_miRNA[(merged_correlation_mRNA_miRNA$result_
cor_AI_and_NB_cor_NP_a== "gain_positive"),]
merged_correlation_mRNA_miRNA_gain_negative<-merged_correlation_mRNA_miRNA[(merged_correlation_mRNA_miRNA$result_
cor_AI_and_NB_cor_NP_a== "gain_negative"),]
merged_correlation_mRNA_miRNA_inverted<-merged_correlation_mRNA_miRNA[(merged_correlation_mRNA_miRNA$result_cor_A
I_and_NB_cor_NP_a== "inverted"),]
```

```
knitr::kable(head(merged_correlation_mRNA_miRNA), caption = "First five rows of Supplementary Table 8",format =
'pandoc')
```

First five rows of Supplementary Table 8

	mRNA_gene	miRNA_gene	cor_AI	pvalue_AI	cor_NB	pvalue_NB	cor_NP	pvalue_NP	mRNA
6583	ENSBTAG00000017803	ENSBTAG00000029760	-0.0688122	0.8969446	-0.0915422	0.8630703	0.9104692	0.0317229	RAB1
10880	ENSBTAG00000000993	ENSBTAG00000029761	-0.0444719	0.9333361	-0.0853261	0.8723215	0.9038630	0.0352619	N4BP
14977	ENSBTAG00000011994	ENSBTAG00000029761	0.0547957	0.9178887	0.0724871	0.8914598	-0.9244153	0.0246603	NDEL
15320	ENSBTAG00000012972	ENSBTAG00000029761	0.0031738	0.9952393	0.0484562	0.9273727	0.9230475	0.0253274	CYP2
15513	ENSBTAG00000013475	ENSBTAG00000029761	0.0925901	0.8615118	0.0820110	0.8772593	0.9289427	0.0224938	TRAF
18569	ENSBTAG00000021859	ENSBTAG00000029761	-0.0431445	0.9353233	0.0254350	0.9618558	0.9867504	0.0018271	PDSS

Figure 3c

```
plot<-ggplot(merged_correlation_mRNA_miRNA, aes(y=(cor_AI+ cor_NB)/2, x=cor_NP))+
geom_point(alpha=0.2, size=1)+
scale_x_continuous("non pregnant")+
scale_y_continuous("Pregnant")+
coord_fixed(ratio = 1)+
#ggtitle("mRNA-miRNA differential coexpression")+
theme_bw()+
theme(
text = element_text(size=10, color="black"),
panel.background = element_rect(fill = "transparent"),
plot.background = element_rect(fill = "transparent", color = NA),
)
#png(filename =paste(result_path,"2019_12_23_plot_mrna_mirna_diff_coexpression.png", sep="/"), width = 2, height
 = 2, units = 'in', res = 300, bg = "transparent")
#plot
#dev.off()
plot
```

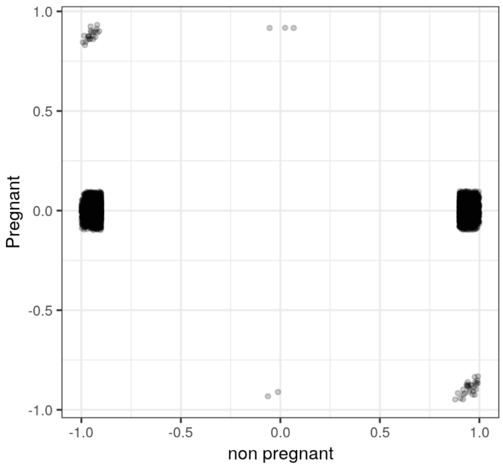

```
merged_correlation_mRNA_miRNA_gain_positive<-merged_correlation_mRNA_miRNA[(merged_correlation_mRNA_miRNA$result_
cor_AI_and_NB_cor_NP_a== "gain_positive"),]
merged_correlation_mRNA_miRNA_gain_positive<-merged_correlation_mRNA_miRNA_gain_positive[order(-merged_correlatio
n_mRNA_miRNA_gain_positive$cor_NP),]


data_for_chart<-data.frame()
i=1
  expression_gene_1_AI<-cpm_AIpreg[row.names(cpm_AIpreg)==as.character(merged_correlation_mRNA_miRNA_gain_positiv
e$mRNA_gene)[i],]
  expression_gene_2_AI<-cpm_AIpreg_miRNA[row.names(cpm_AIpreg_miRNA)==as.character(merged_correlation_mRNA_miRNA_
gain_positive$miRNA_gene)[i],]
  expression_gene_1_NB<-cpm_NBpreg[row.names(cpm_NBpreg)==as.character(merged_correlation_mRNA_miRNA_gain_positiv
e$mRNA_gene)[i],]
  expression_gene_2_NB<-cpm_NBpreg_miRNA[row.names(cpm_NBpreg_miRNA)==as.character(merged_correlation_mRNA_miRNA_
gain_positive$miRNA_gene)[i],]
  expression_gene_1_NP<-cpm_NOTpreg[row.names(cpm_NOTpreg)==as.character(merged_correlation_mRNA_miRNA_gain_posit
ive$mRNA_gene)[i],]
  expression_gene_2_NP<-cpm_NOTpreg_miRNA[row.names(cpm_NOTpreg_miRNA)==as.character(merged_correlation_mRNA_miRN
A_gain_positive$miRNA_gene)[i],]

  symbol_gene_1<-annotation.ensembl.symbol[annotation.ensembl.symbol$ensembl_gene_id == as.character(merged_corre
lation_mRNA_miRNA_gain_positive$mRNA_gene)[i] , 2]
  symbol_gene_2<-annotation.ensembl.symbol[annotation.ensembl.symbol$ensembl_gene_id == as.character(merged_corre
lation_mRNA_miRNA_gain_positive$miRNA_gene)[i] , 2]

  data_for_chart<-data.frame(gene_1=c(expression_gene_1_AI,expression_gene_1_NB,expression_gene_1_NP) ,
                             gene_2=c(expression_gene_2_AI,expression_gene_2_NB,expression_gene_2_NP),
                             symbol_gene_1,symbol_gene_2 ,chart,
                             group=rep(c("AI", "NB", "NP"), c(length(expression_gene_1_AI),length(expression_gene
_1_NB),length(expression_gene_1_NP))))

font_axis<-5
font_title<-8
ptsize<-0.05

plot<-ggplot(data_for_chart, aes(x=log(gene_1+1),y=log(gene_2+1)))+
  geom_point()+
  geom_smooth(method=lm, size=ptsize, fill="lightgray")+
  scale_y_continuous(name=data_for_chart$symbol_gene_1[1])+
  scale_x_continuous(name=data_for_chart$symbol_gene_2[1])+
  facet_wrap(~group, ncol=3, scales="free")+
  theme(aspect.ratio = 1,
            panel.grid.major = element_blank(),
            panel.grid.minor = element_blank(),
            panel.background = element_blank(),
            plot.background  = element_blank(),
            axis.text.y = element_text( colour = 'black',size = font_axis),
            axis.text.x = element_text( colour = 'black',size = font_axis, angle=90),
            axis.title = element_text( colour = 'black' ,size = font_title, face="italic"),
            axis.ticks = element_line(size=0.1),
            panel.spacing.y = unit(0, "mm"),
            panel.spacing.x = unit(2, "mm"),
            legend.position="none",
            axis.line=element_line(size = 0.1, colour = "black"),
            strip.background=element_blank(),
            strip.text.x = element_blank())

#png(filename =paste(result_path,"2019_12_23_plot_diff_cor_mRNA_miRNA_AI_NB_and_NP_gain_positive.png", sep="/"),
 width = 2.5, height =1, units = 'in', res = 300,bg = "transparent")
#plot
#dev.off()
plot
```

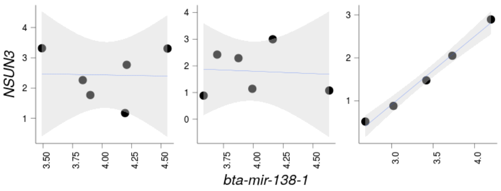

```
merged_correlation_mRNA_miRNA_gain_negative<-merged_correlation_mRNA_miRNA[(merged_correlation_mRNA_miRNA$result_
cor_AI_and_NB_cor_NP_a== "gain_negative"),]
merged_correlation_mRNA_miRNA_gain_negative<-merged_correlation_mRNA_miRNA_gain_negative[order(merged_correlation
_mRNA_miRNA_gain_negative$cor_NP),]


data_for_chart<-data.frame()
i=1
  expression_gene_1_AI<-cpm_AIpreg[row.names(cpm_AIpreg)==as.character(merged_correlation_mRNA_miRNA_gain_negativ
e$mRNA_gene)[i],]
  expression_gene_2_AI<-cpm_AIpreg_miRNA[row.names(cpm_AIpreg_miRNA)==as.character(merged_correlation_mRNA_miRNA_
gain_negative$miRNA_gene)[i],]
  expression_gene_1_NB<-cpm_NBpreg[row.names(cpm_NBpreg)==as.character(merged_correlation_mRNA_miRNA_gain_negativ
e$mRNA_gene)[i],]
  expression_gene_2_NB<-cpm_NBpreg_miRNA[row.names(cpm_NBpreg_miRNA)==as.character(merged_correlation_mRNA_miRNA_
gain_negative$miRNA_gene)[i],]
  expression_gene_1_NP<-cpm_NOTpreg[row.names(cpm_NOTpreg)==as.character(merged_correlation_mRNA_miRNA_gain_negat
ive$mRNA_gene)[i],]
  expression_gene_2_NP<-cpm_NOTpreg_miRNA[row.names(cpm_NOTpreg_miRNA)==as.character(merged_correlation_mRNA_miRN
A_gain_negative$miRNA_gene)[i],]

  symbol_gene_1<-annotation.ensembl.symbol[annotation.ensembl.symbol$ensembl_gene_id == as.character(merged_corre
lation_mRNA_miRNA_gain_negative$mRNA_gene)[i] , 2]
  symbol_gene_2<-annotation.ensembl.symbol[annotation.ensembl.symbol$ensembl_gene_id == as.character(merged_corre
lation_mRNA_miRNA_gain_negative$miRNA_gene)[i] , 2]

  data_for_chart<-data.frame(gene_1=c(expression_gene_1_AI,expression_gene_1_NB,expression_gene_1_NP) ,
                             gene_2=c(expression_gene_2_AI,expression_gene_2_NB,expression_gene_2_NP),
                             symbol_gene_1,symbol_gene_2 ,chart,
                             group=rep(c("AI", "NB", "NP"), c(length(expression_gene_1_AI),length(expression_gene
_1_NB),length(expression_gene_1_NP))))

font_axis<-5
font_title<-8
ptsize<-0.05

plot<-ggplot(data_for_chart, aes(x=log(gene_1+1),y=log(gene_2+1)))+
  geom_point()+
  geom_smooth(method=lm, size=ptsize, fill="lightgray")+
  scale_y_continuous(name=data_for_chart$symbol_gene_1[1])+
  scale_x_continuous(name=data_for_chart$symbol_gene_2[1])+
  facet_wrap(~group, ncol=3, scales="free")+
  theme(aspect.ratio = 1,
            panel.grid.major = element_blank(),
            panel.grid.minor = element_blank(),
            panel.background = element_blank(),
            plot.background  = element_blank(),
            axis.text.y = element_text( colour = 'black',size = font_axis),
            axis.text.x = element_text( colour = 'black',size = font_axis, angle=90),
            axis.title = element_text( colour = 'black' ,size = font_title, face="italic"),
            axis.ticks = element_line(size=0.1),
            panel.spacing.y = unit(0, "mm"),
            panel.spacing.x = unit(2, "mm"),
            legend.position="none",
            axis.line=element_line(size = 0.1, colour = "black"),
            strip.background=element_blank(),
            strip.text.x = element_blank())

#png(filename =paste(result_path,"2019_12_23_plot_diff_cor_mRNA_miRNA_AI_NB_and_NP_gain_negative.png", sep="/"),
 width = 2.5, height =1, units = 'in', res = 300,bg = "transparent")
#plot
#dev.off()
plot
```

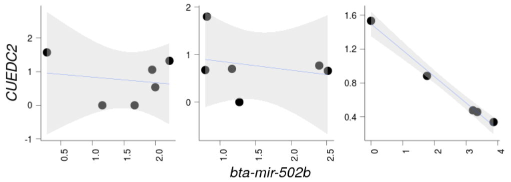

```
merged_correlation_mRNA_miRNA_inverted<-merged_correlation_mRNA_miRNA[(merged_correlation_mRNA_miRNA$result_cor_A
I_and_NB_cor_NP_a== "inverted"),]
merged_correlation_mRNA_miRNA_inverted<-merged_correlation_mRNA_miRNA_inverted[order(merged_correlation_mRNA_miRN
A_inverted$cor_NP),]


data_for_chart<-data.frame()
i=1
  expression_gene_1_AI<-cpm_AIpreg[row.names(cpm_AIpreg)==as.character(merged_correlation_mRNA_miRNA_inverted$mRN
A_gene)[i],]
  expression_gene_2_AI<-cpm_AIpreg_miRNA[row.names(cpm_AIpreg_miRNA)==as.character(merged_correlation_mRNA_miRNA_
inverted$miRNA_gene)[i],]
  expression_gene_1_NB<-cpm_NBpreg[row.names(cpm_NBpreg)==as.character(merged_correlation_mRNA_miRNA_inverted$mRN
A_gene)[i],]
  expression_gene_2_NB<-cpm_NBpreg_miRNA[row.names(cpm_NBpreg_miRNA)==as.character(merged_correlation_mRNA_miRNA_
inverted$miRNA_gene)[i],]
  expression_gene_1_NP<-cpm_NOTpreg[row.names(cpm_NOTpreg)==as.character(merged_correlation_mRNA_miRNA_inverted$m
RNA_gene)[i],]
  expression_gene_2_NP<-cpm_NOTpreg_miRNA[row.names(cpm_NOTpreg_miRNA)==as.character(merged_correlation_mRNA_miRN
A_inverted$miRNA_gene)[i],]

  symbol_gene_1<-annotation.ensembl.symbol[annotation.ensembl.symbol$ensembl_gene_id == as.character(merged_corre
lation_mRNA_miRNA_inverted$mRNA_gene)[i] , 2]
  symbol_gene_2<-annotation.ensembl.symbol[annotation.ensembl.symbol$ensembl_gene_id == as.character(merged_corre
lation_mRNA_miRNA_inverted$miRNA_gene)[i] , 2]

  data_for_chart<-data.frame(gene_1=c(expression_gene_1_AI,expression_gene_1_NB,expression_gene_1_NP) ,
                             gene_2=c(expression_gene_2_AI,expression_gene_2_NB,expression_gene_2_NP),
                             symbol_gene_1,symbol_gene_2 ,chart,
                             group=rep(c("AI", "NB", "NP"), c(length(expression_gene_1_AI),length(expression_gene
_1_NB),length(expression_gene_1_NP))))

font_axis<-5
font_title<-8
ptsize<-0.05

plot<-ggplot(data_for_chart, aes(x=log(gene_1+1),y=log(gene_2+1)))+
  geom_point()+
  geom_smooth(method=lm, size=ptsize, fill="lightgray")+
  scale_y_continuous(name=data_for_chart$symbol_gene_1[1])+
  scale_x_continuous(name=data_for_chart$symbol_gene_2[1])+
  facet_wrap(~group, ncol=3, scales="free")+
  theme(aspect.ratio = 1,
          panel.grid.major = element_blank(),
          panel.grid.minor = element_blank(),
          panel.background = element_blank(),
          plot.background  = element_blank(),
          axis.text.y = element_text( colour = 'black',size = font_axis),
          axis.text.x = element_text( colour = 'black',size = font_axis, angle=90),
          axis.title = element_text( colour = 'black' ,size = font_title, face="italic"),
          axis.ticks = element_line(size=0.1),
          panel.spacing.y = unit(0, "mm"),
          panel.spacing.x = unit(2, "mm"),
          legend.position="none",
          axis.line=element_line(size = 0.1, colour = "black"),
          strip.background=element_blank(),
          strip.text.x = element_blank())

#png(filename =paste(result_path,"2019_12_23_plot_diff_cor_mRNA_miRNA_AI_NB_and_NP_inverted_negative.png", sep
="/"), width = 2.5, height =1, units = 'in', res = 300,bg = "transparent")
#plot
#dev.off()
plot
```

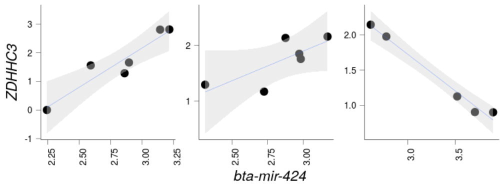

```
merged_correlation_mRNA_miRNA_inverted<-merged_correlation_mRNA_miRNA_inverted[order(-merged_correlation_mRNA_miR
NA_inverted$cor_NP),]

data_for_chart<-data.frame()
i=5
  expression_gene_1_AI<-cpm_AIpreg[row.names(cpm_AIpreg)==as.character(merged_correlation_mRNA_miRNA_inverted$mRN
A_gene)[i],]
  expression_gene_2_AI<-cpm_AIpreg_miRNA[row.names(cpm_AIpreg_miRNA)==as.character(merged_correlation_mRNA_miRNA_
inverted$miRNA_gene)[i],]
  expression_gene_1_NB<-cpm_NBpreg[row.names(cpm_NBpreg)==as.character(merged_correlation_mRNA_miRNA_inverted$mRN
A_gene)[i],]
  expression_gene_2_NB<-cpm_NBpreg_miRNA[row.names(cpm_NBpreg_miRNA)==as.character(merged_correlation_mRNA_miRNA_
inverted$miRNA_gene)[i],]
  expression_gene_1_NP<-cpm_NOTpreg[row.names(cpm_NOTpreg)==as.character(merged_correlation_mRNA_miRNA_inverted$m
RNA_gene)[i],]
  expression_gene_2_NP<-cpm_NOTpreg_miRNA[row.names(cpm_NOTpreg_miRNA)==as.character(merged_correlation_mRNA_miRN
A_inverted$miRNA_gene)[i],]

  symbol_gene_1<-annotation.ensembl.symbol[annotation.ensembl.symbol$ensembl_gene_id == as.character(merged_corre
lation_mRNA_miRNA_inverted$mRNA_gene)[i] , 2]
  symbol_gene_2<-annotation.ensembl.symbol[annotation.ensembl.symbol$ensembl_gene_id == as.character(merged_corre
lation_mRNA_miRNA_inverted$miRNA_gene)[i] , 2]

  data_for_chart<-data.frame(gene_1=c(expression_gene_1_AI,expression_gene_1_NB,expression_gene_1_NP) ,
                             gene_2=c(expression_gene_2_AI,expression_gene_2_NB,expression_gene_2_NP),
                             symbol_gene_1,symbol_gene_2 ,chart,
                             group=rep(c("AI", "NB", "NP"), c(length(expression_gene_1_AI),length(expression_gene
_1_NB),length(expression_gene_1_NP))))

font_axis<-5
font_title<-8
ptsize<-0.05

plot<-ggplot(data_for_chart, aes(x=log(gene_1+1),y=log(gene_2+1)))+
  geom_point()+
  geom_smooth(method=lm, size=ptsize, fill="lightgray")+
  scale_y_continuous(name=data_for_chart$symbol_gene_1[1])+
  scale_x_continuous(name=data_for_chart$symbol_gene_2[1])+
  facet_wrap(~group, ncol=3, scales="free")+
  theme(aspect.ratio = 1,
            panel.grid.major = element_blank(),
            panel.grid.minor = element_blank(),
            panel.background = element_blank(),
            plot.background  = element_blank(),
            axis.text.y = element_text( colour = 'black',size = font_axis),
            axis.text.x = element_text( colour = 'black',size = font_axis, angle=90),
            axis.title = element_text( colour = 'black' ,size = font_title, face="italic"),
            axis.ticks = element_line(size=0.1),
            panel.spacing.y = unit(0, "mm"),
            panel.spacing.x = unit(2, "mm"),
            legend.position="none",
            axis.line=element_line(size = 0.1, colour = "black"),
            strip.background=element_blank(),
            strip.text.x = element_blank())

#png(filename =paste(result_path,"2019_12_23_plot_diff_cor_mRNA_miRNA_AI_NB_and_NP_inverted_positive.png", sep
="/"), width = 2.5, height =1, units = 'in', res = 300,bg = "transparent")
#plot
#dev.off()
plot
```

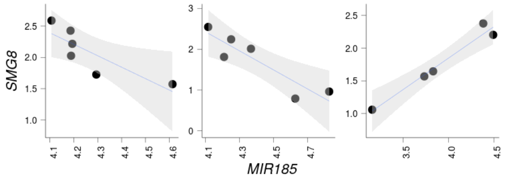

Supplementary Table 9

```
test.genes<-data.frame(a=unique(c(as.character(merged_correlation_mRNA_miRNA_gain_negative$mRNA_gene))), stringsA
sFactors=FALSE)
all_genes_numeric<-as.integer(all_genes$gene %in%test.genes$a)
names(all_genes_numeric)<-all_genes$gene

N_DEGs<-length(test.genes$a)

set.seed(9830)
pwf<-nullp(all_genes_numeric, bias.data=annotation.genelength.biomart_vector, plot.fit=FALSE )
GO_BP_Cats_mRNA_AI_NB_Preg_NP<-goseq(pwf,gene2cat=annotation.GO.BP.biomart, method ="Sampling", repcnt = 7000, us
e_genes_without_cat=FALSE)
GO_BP_Cats_mRNA_AI_NB_Preg_NP<-GO_BP_Cats_mRNA_AI_NB_Preg_NP[GO_BP_Cats_mRNA_AI_NB_Preg_NP$numDEInCat>4,]
GO_BP_Cats_mRNA_AI_NB_Preg_NP$BY_FDR<-p.adjust(GO_BP_Cats_mRNA_AI_NB_Preg_NP$over_represented_pvalue, method ="fd
r")
GO_BP_Cats_mRNA_AI_NB_Preg_NP<-GO_BP_Cats_mRNA_AI_NB_Preg_NP[with(GO_BP_Cats_mRNA_AI_NB_Preg_NP, order(BY_FDR,ove
r_represented_pvalue, -numDEInCat)), ]
#head(GO_BP_Cats_mRNA_AI_NB_Preg_NP, n=20)

GO_BP_Cats_mRNA_AI_NB_Preg_NP$fold_enrichment<-(GO_BP_Cats_mRNA_AI_NB_Preg_NP$numDEInCat/N_DEGs)/(GO_BP_Cats_mRNA
_AI_NB_Preg_NP$numInCat/N_expressed_genes)
annotation.GO.BP.biomart_testgenes<-annotation.GO.BP.biomart[annotation.GO.BP.biomart$ensembl_gene_id %in% test.g
enes$a, ]
GO_BP_Cats_mRNA_AI_NB_Preg_NP<-merge(GO_BP_Cats_mRNA_AI_NB_Preg_NP,annotation.GO.BP.biomart_testgenes, by.x="cate
gory", by.y="go_id", all.x=TRUE, all.y=FALSE)
GO_BP_Cats_mRNA_AI_NB_Preg_NP<-merge(GO_BP_Cats_mRNA_AI_NB_Preg_NP, annotation.ensembl.symbol, by.x="ensembl_gene
_id", by.y="ensembl_gene_id", all=FALSE, all.x=TRUE, all.y=FALSE)
GO_BP_Cats_mRNA_AI_NB_Preg_NP<-GO_BP_Cats_mRNA_AI_NB_Preg_NP[with(GO_BP_Cats_mRNA_AI_NB_Preg_NP, order(BY_FDR,ter
m)), ]
GO_BP_Cats_mRNA_AI_NB_Preg_NP<-GO_BP_Cats_mRNA_AI_NB_Preg_NP[GO_BP_Cats_mRNA_AI_NB_Preg_NP$BY_FDR < 0.1,]

#write.table(GO_BP_Cats_mRNA_AI_NB_Preg_NP, paste(result_path, "2019_12_23_GO_BP_Cats_mRNA_miRNA_gain_neg_coexpre
ssion_AI_NB_Preg_NP.txt", sep="/"), quote=FALSE, sep="\t", row.names=FALSE)
```

```
knitr::kable(head(GO_BP_Cats_mRNA_AI_NB_Preg_NP), caption = "First five rows of Supplementary Table 9",format =
'pandoc')
```

First five rows of Supplementary Table 9

	ensembl_gene_id	category	over_represented_pvalue	under_represented_pvalue	numDEInCat	numInCat	term	ontology
55	ENSBTAG00000000563	GO:0016064 (GO:0016064)	0.0005713	1.0000000	5	8	immunoglobulin mediated immune response	BP
1343	ENSBTAG00000015228	GO:0016064 (GO:0016064)	0.0005713	1.0000000	5	8	immunoglobulin mediated immune response	BP
1979	ENSBTAG00000024503	GO:0016064 (GO:0016064)	0.0005713	1.0000000	5	8	immunoglobulin mediated immune response	BP
2126	ENSBTAG00000032122	GO:0016064 (GO:0016064)	0.0005713	1.0000000	5	8	immunoglobulin mediated immune response	BP
2289	ENSBTAG00000047680	GO:0016064 (GO:0016064)	0.0005713	1.0000000	5	8	immunoglobulin mediated immune response	BP
37	ENSBTAG00000000396	GO:0043433 (GO:0043433)	0.0004285	0.9998572	11	34	negative regulation of DNA-binding transcription factor activity	BP

Supplementary Table 10

```
test.genes<-data.frame(a=unique(c(as.character(merged_correlation_mRNA_miRNA_gain_positive$mRNA_gene))), stringsA
sFactors=FALSE)
all_genes_numeric<-as.integer(all_genes$gene %in%test.genes$a)
names(all_genes_numeric)<-all_genes$gene

N_DEGs<-length(test.genes$a)

set.seed(9830)
pwf<-nullp(all_genes_numeric, bias.data=annotation.genelength.biomart_vector, plot.fit=FALSE )
```

```
## Warning in pcls(G): initial point very close to some inequality constraints
```

```
GO_BP_Cats_mRNA_AI_NB_Preg_NP<-goseq(pwf,gene2cat=annotation.GO.BP.biomart, method ="Sampling", repcnt = 7000, us
e_genes_without_cat=FALSE)
GO_BP_Cats_mRNA_AI_NB_Preg_NP<-GO_BP_Cats_mRNA_AI_NB_Preg_NP[GO_BP_Cats_mRNA_AI_NB_Preg_NP$numDEInCat>4,]
GO_BP_Cats_mRNA_AI_NB_Preg_NP$BY_FDR<-p.adjust(GO_BP_Cats_mRNA_AI_NB_Preg_NP$over_represented_pvalue, method ="fd
r")
GO_BP_Cats_mRNA_AI_NB_Preg_NP<-GO_BP_Cats_mRNA_AI_NB_Preg_NP[with(GO_BP_Cats_mRNA_AI_NB_Preg_NP, order(BY_FDR,ove
r_represented_pvalue, -numDEInCat)), ]
#head(GO_BP_Cats_mRNA_AI_NB_Preg_NP, n=20)

GO_BP_Cats_mRNA_AI_NB_Preg_NP$fold_enrichment<-(GO_BP_Cats_mRNA_AI_NB_Preg_NP$numDEInCat/N_DEGs)/(GO_BP_Cats_mRNA
_AI_NB_Preg_NP$numInCat/N_expressed_genes)
annotation.GO.BP.biomart_testgenes<-annotation.GO.BP.biomart[annotation.GO.BP.biomart$ensembl_gene_id %in% test.g
enes$a, ]
GO_BP_Cats_mRNA_AI_NB_Preg_NP<-merge(GO_BP_Cats_mRNA_AI_NB_Preg_NP,annotation.GO.BP.biomart_testgenes, by.x="cate
gory", by.y="go_id", all.x=TRUE, all.y=FALSE)
GO_BP_Cats_mRNA_AI_NB_Preg_NP<-merge(GO_BP_Cats_mRNA_AI_NB_Preg_NP, annotation.ensembl.symbol, by.x="ensembl_gene
_id", by.y="ensembl_gene_id", all=FALSE, all.x=TRUE, all.y=FALSE)
GO_BP_Cats_mRNA_AI_NB_Preg_NP<-GO_BP_Cats_mRNA_AI_NB_Preg_NP[with(GO_BP_Cats_mRNA_AI_NB_Preg_NP, order(BY_FDR,ter
m)), ]
GO_BP_Cats_mRNA_AI_NB_Preg_NP<-GO_BP_Cats_mRNA_AI_NB_Preg_NP[GO_BP_Cats_mRNA_AI_NB_Preg_NP$BY_FDR < 0.1,]
#write.table(GO_BP_Cats_mRNA_AI_NB_Preg_NP, paste(result_path, "2019_12_23_GO_BP_Cats_mRNA_miRNA_gain_positive_co
expression_AI_NB_Preg_NP.txt", sep="/"), quote=FALSE, sep="\t", row.names=FALSE)
```

```
knitr::kable(head(GO_BP_Cats_mRNA_AI_NB_Preg_NP), caption = "First five rows of Supplementary Table 10",format =
'pandoc')
```

First five rows of Supplementary Table 10

	ensembl_gene_id	category	over_represented_pvalue	under_represented_pvalue	numDEInCat	numInCat	term	ontology	BY
683	ENSBTAG00000006065	GO:0006275 (GO:0006275)	0.0001428	1	8	15	regulation of DNA replication	BP	0.04
755	ENSBTAG00000006551	GO:0006275 (GO:0006275)	0.0001428	1	8	15	regulation of DNA replication	BP	0.04
1550	ENSBTAG00000013905	GO:0006275 (GO:0006275)	0.0001428	1	8	15	regulation of DNA replication	BP	0.04
1729	ENSBTAG00000015338	GO:0006275 (GO:0006275)	0.0001428	1	8	15	regulation of DNA replication	BP	0.04
1878	ENSBTAG00000016572	GO:0006275 (GO:0006275)	0.0001428	1	8	15	regulation of DNA replication	BP	0.04
1984	ENSBTAG00000017329	GO:0006275 (GO:0006275)	0.0001428	1	8	15	regulation of DNA replication	BP	0.04

```
gc()
```

Differential gene expression associated with pregnancy outcome

AI Pregnant vs non pregnant

Results from edgeR

```
#colnames(count_pwbc)
group<-factor(c("Preg_AI","Preg_AI","Preg_AI","Preg_AI","Preg_AI","Preg_AI","Preg_NB","Preg_NB","Preg_NB","Preg_N
B","Preg_NB","Preg_NB","Not_Preg","Not_Preg","Not_Preg","Not_Preg","Not_Preg"), levels=c("Preg_AI","Preg_NB", "No
t_Preg"))
design<-model.matrix(~group)
dds<-DGEList(count=count_pwbc, group=group)
dds<-estimateDisp(dds, design, robust=TRUE)
dds<-glmFit(dds, design)
dds<-glmLRT(dds,coef="groupNot_Preg")
res_not_preg_preg_AI_edgeR<-topTags(dds,n=Inf)$table
#head(res_not_preg_preg_AI_edgeR,n=30)
#write.table(res_not_preg_preg_AI_edgeR, paste(result_path, "2019_04_30_edgeR_AI_NP_mRNA.txt"), quote=FALSE, sep
="\t", row.names=FALSE)
```

Results from edgeR both years

```
#colnames(merged_datasets)
group<-factor(c("Preg_AI","Preg_AI","Preg_AI","Preg_AI","Preg_AI","Preg_AI","Preg_NB","Preg_NB","Preg_NB","Preg_N
B","Preg_NB","Preg_NB","Not_Preg","Not_Preg","Not_Preg","Not_Preg","Not_Preg",
                "Preg_AI","Not_Preg", "Not_Preg", "Not_Preg", "Preg_AI", "Preg_AI","Preg_AI", "Preg_AI", "Not_Pre
g", "Preg_AI",
                "Not_Preg", "Not_Preg"), levels=c("Preg_AI","Preg_NB", "Not_Preg"))
year<-factor(rep(c("A","B"), c(17,12)))
design<-model.matrix(~0+group+year)
dds<-DGEList(count=merged_datasets, group=group)
dds<-estimateDisp(dds, design, robust=TRUE)
contrast_not_preg_preg_AI<-makeContrasts( groupNot_Preg-groupPreg_AI, levels=design)
dds<-glmFit(dds, design)
dds<-glmLRT(dds,contrast=contrast_not_preg_preg_AI)
res_not_preg_preg_AI_edgeR_merged<-topTags(dds,n=Inf)$table
#head(res_not_preg_preg_AI_edgeR_merged,n=30)
#write.table(res_not_preg_preg_AI_edgeR, paste(result_path, "2019_04_16_edgeR_AI_NP_mRNA.txt"), quote=FALSE, sep
="\t", row.names=FALSE)
```

Results from DESeq

```
group<-factor(c("Preg_AI","Preg_AI","Preg_AI","Preg_AI","Preg_AI","Preg_AI","Preg_NB","Preg_NB","Preg_NB","Preg_N
B","Preg_NB","Preg_NB","Not_Preg","Not_Preg","Not_Preg","Not_Preg","Not_Preg"), levels=c("Preg_AI","Preg_NB", "No
t_Preg"))
design<-model.matrix(~group)
colData<-data.frame("group"=group)
rownames(colData)<-colnames(count_pwbc)
dds<-DESeqDataSetFromMatrix(countData=count_pwbc, colData=colData, design= ~group)
dds<-DESeq(dds)
res_not_preg_preg_AI_DeSeq<-results(dds, contrast=c("group","Not_Preg" ,"Preg_AI"), pAdjustMethod="fdr", tidy=TRU
E)
res_not_preg_preg_AI_DeSeq<-res_not_preg_preg_AI_DeSeq[with(res_not_preg_preg_AI_DeSeq, order(pvalue)), ]
#head(res_not_preg_preg_AI_DeSeq,n=30)
#write.table(res_not_preg_preg_AI_DeSeq, paste(result_path, "2019_04_30_DESeq_AI_NP_mRNA.txt"), quote=FALSE, sep
="\t", row.names=FALSE)
```

Results from DESeq from both years

```
group<-factor(c("Preg_AI","Preg_AI","Preg_AI","Preg_AI","Preg_AI","Preg_AI","Preg_NB","Preg_NB","Preg_NB","Preg_N
B","Preg_NB","Preg_NB","Not_Preg","Not_Preg","Not_Preg","Not_Preg","Not_Preg",
                "Preg_AI","Not_Preg", "Not_Preg", "Not_Preg", "Preg_AI", "Preg_AI","Preg_AI", "Preg_AI", "Not_Pre
g", "Preg_AI",
                "Not_Preg", "Not_Preg"), levels=c("Preg_AI","Preg_NB", "Not_Preg"))
design<-model.matrix(~group+year)
colData<-data.frame("group"=group, "year"=year)
rownames(colData)<-colnames(merged_datasets)
dds<-DESeqDataSetFromMatrix(countData=merged_datasets, colData=colData, design= ~group+year)
dds<-DESeq(dds)
res_not_preg_preg_AI_DeSeq_merged<-results(dds, contrast=c("group", "Not_Preg", "Preg_AI") , pAdjustMethod="fdr",
tidy=TRUE)
res_not_preg_preg_AI_DeSeq_merged<-res_not_preg_preg_AI_DeSeq_merged[with(res_not_preg_preg_AI_DeSeq_merged, orde
r(pvalue)), ]
#head(res_not_preg_preg_AI_DeSeq_merged,n=30)
#write.table(res_not_preg_preg_AI_DeSeq, paste(result_path, "2019_04_22_DESeq_AI_NP_mRNA.txt"), quote=FALSE, sep
="\t", row.names=FALSE)
```

Supplementary Fig. 4

```
setwd("/mnt/storage/auburn/heifer_pregnancy/proj_2018/analysis/results/")
#Create the permutations matrix
perm_without_replacement <- function(n, r){
  return(factorial(n)/factorial(n - r))
}

permutation_matrix<-matrix(,nrow=200100,ncol=17)
head(permutation_matrix)
dim(permutation_matrix)

for (i in seq(1:200100)){
  sampling <- sample(1:17,17, replace = FALSE)
  if ( ! identical(sampling , c(1:17))){
    permutation_matrix[i,]<-sample(1:17,17, replace = FALSE)
  }}

permutation_matrix<-permutation_matrix[!duplicated(permutation_matrix),]
dim(permutation_matrix)
head(permutation_matrix)

permutation_matrix<-permutation_matrix[sample(1:dim(permutation_matrix)[1], 10000),]
rand<-dim(permutation_matrix)[1]

sequence.pvalue<-seq(0.005, 0.05, 0.005)

#edgeR
results <- filebacked.big.matrix(length(sequence.pvalue),rand, type="double", init=0, separated=FALSE,
                                 backingfile="incidence_matrix.bin",
                                 descriptor="incidence_matrix.desc")
mdesc_result<- describe(results)

#edgeR
cl <- makeCluster(30)
registerDoParallel(cl)

results[,]<-foreach(i = sequence.pvalue, .combine='rbind', .inorder=TRUE,  .packages=c("edgeR","bigmemory"), .ver
bose=FALSE) %:%

  foreach(j = 1:rand, .combine='cbind', .inorder=FALSE,.packages=c("edgeR","bigmemory"), .verbose=FALSE ) %dopar%
{

    group<-factor(c("Preg_AI","Preg_AI","Preg_AI","Preg_AI","Preg_AI","Preg_AI","Preg_NB","Preg_NB","Preg_NB","Pr
eg_NB","Preg_NB","Preg_NB","Not_Preg","Not_Preg","Not_Preg","Not_Preg","Not_Preg"), levels=c("Preg_AI","Preg_NB",
"Not_Preg"))
    group<-group[permutation_matrix[j,]]
    group<-factor(group, levels=c("Preg_AI","Not_Preg", "Preg_NB"))
    design <- model.matrix(~group)
    dds<-DGEList(count=count_pwbc, group=group)
    dds<-estimateDisp(dds, design, robust=TRUE)
    dds <- glmFit(dds, design)
    dds <- glmLRT(dds, coef="groupNot_Preg")
    length(which(topTags(dds,n=Inf)$table$PValue < i))

  }
stopCluster(cl)
results[1:5,1:5]
total.rand <- rand * 10496

qvalue<-data.frame(raw.pvalue = sequence.pvalue,
                   e.pvalue.edgeR= (rowSums(results[,]+1))/(total.rand+1),
                   e.pvalue.edgeR.round= round((rowSums(results[,]+1))/(total.rand+1) ,4))

#qvalue
rm(results)
system("rm incidence_matrix.bin")
system("rm incidence_matrix.desc")

#DESEQ2

results.rand <- filebacked.big.matrix(length(sequence.pvalue),rand, type="double", init=0, separated=FALSE,
                                      backingfile="incidence_matrix.bin",
                                      descriptor="incidence_matrix.desc")
mdesc_result<- describe(results.rand)

cl <- makeCluster(30)
registerDoParallel(cl)

results.rand[,]<-foreach(i = sequence.pvalue, .combine='rbind', .inorder=TRUE,  .packages=c("DESeq2","bigmemory"
), .verbose=TRUE) %:%

  foreach(j = 1:rand, .combine='cbind', .inorder=FALSE,.packages=c("DESeq2","bigmemory"), .verbose=TRUE ) %dopar%
```

```
{

    group<- c("Preg_AI","Preg_AI","Preg_AI","Preg_AI","Preg_AI","Preg_AI","Preg_NB","Preg_NB","Preg_NB","Preg_NB"
,"Preg_NB","Preg_NB","Not_Preg","Not_Preg","Not_Preg","Not_Preg","Not_Preg")
    group<-group[permutation_matrix[j,]]
    group<-factor(group, levels=c("Preg_AI","Not_Preg", "Preg_NB"))
    design <- model.matrix(~group)

    colData<-data.frame("group"=group)
    rownames(colData)<-colnames(count_pwbc)
    dds<-DESeqDataSetFromMatrix(countData=count_pwbc, colData=colData, design= ~group)
    dds <- DESeq(dds)
    DeSeq_AI_NP <- results(dds, contrast=c("group","Not_Preg" ,"Preg_AI"), pAdjustMethod="fdr", tidy=TRUE)

    length(which(DeSeq_AI_NP$pvalue < i))

  }

stopCluster(cl)

results.rand[1:5,1:5]

total.rand <- rand * 10496


qvalue2<-data.frame(raw.pvalue = sequence.pvalue,
                    e.pvalue.DESEQ2= (rowSums(results.rand[,]+1))/(total.rand+1),
                    e.pvalue.DESEQ2.round= round((rowSums(results.rand[,]+1))/(total.rand+1) ,4))

qvalue2


qvalue3<-cbind(qvalue,qvalue2)

qvalue3

rm(results.rand)
system("rm incidence_matrix.bin")
system("rm incidence_matrix.desc")

#write.table(qvalue3,paste(result_path,"2019_12_26_empirical_FDR_for_DEG.txt",sep="/"), quote=FALSE, sep="\t", ro
w.names=FALSE)
```

```
eFDR_differential_expression<-read.delim(paste(result_path,"2019_12_26_empirical_FDR_for_DEG.txt",sep="/"), row.n
ames=NULL,header =TRUE, stringsAsFactors =FALSE)
```

```
font_size<-9

plot1<-ggplot()+
  geom_point(data=eFDR_differential_expression, aes(x=e.pvalue.edgeR.round , y=raw.pvalue),color="black", size=3,
shape=16)+
  geom_line(data=eFDR_differential_expression, aes(x=e.pvalue.edgeR.round , y=raw.pvalue),color="black", size=0.1
,linetype=3)+
  scale_x_continuous(name="empirical FDR", limits = c(0, 0.08), breaks=seq(0, 0.08, 0.005))+
  scale_y_continuous(name="nominal P value", limits = c(0, 0.05), breaks=seq(0, 0.06, 0.005))+
  ggtitle("Differential gene expression with edgeR")+
  theme_bw()+
  theme(panel.grid= element_blank(),
        panel.background = element_blank(),
        panel.grid.minor = element_blank(),
        panel.grid.major = element_line(color="lightgray"),
        plot.background = element_blank(),
        axis.text.y=element_text(color="black", size=font_size),
        axis.text.x=element_text(color="black", size=font_size, angle=90),
        panel.spacing = unit(c(0.4,0.4,0.4,0.4),"cm"),
        plot.margin = unit(c(0.5,0.5,0.5,0.5),"cm"),
        legend.position="none",
        plot.title = element_text(lineheight=.8, hjust=0.5, size= font_size))

plot2<-ggplot()+
  geom_point(data=eFDR_differential_expression, aes(x=e.pvalue.DESEQ2.round , y=raw.pvalue),color="black", size=3
, shape=16)+
  geom_line(data=eFDR_differential_expression, aes(x=e.pvalue.DESEQ2.round , y=raw.pvalue),color="black", size=0.
1,linetype=3)+
  scale_x_continuous(name="empirical FDR", limits = c(0, 0.08), breaks=seq(0, 0.08, 0.005))+
  scale_y_continuous(name="nominal P value", limits = c(0, 0.05), breaks=seq(0, 0.05, 0.005))+
  ggtitle("Differential gene expression with DESEQ2")+
  theme_bw()+
  theme(panel.grid= element_blank(),
        panel.background = element_blank(),
        panel.grid.minor = element_blank(),
        panel.grid.major = element_line(color="lightgray"),
        plot.background = element_blank(),
        axis.title=element_text(color="black", size=font_size),
        axis.text.y=element_text(color="black", size=font_size),
        axis.text.x=element_text(color="black", size=font_size, angle=90),
        panel.spacing = unit(c(0.4,0.4,0.4,0.4),"cm"),
        plot.margin = unit(c(0.5,0.5,0.5,0.5),"cm"),
        legend.position="none",
        plot.title = element_text(lineheight=.8, hjust=0.5, size= font_size))

#font_size 12 for figure
#pdf(paste(result_path,"2019_12_27_eFDR_plots_differential_gene_expression.pdf", sep="/"), width=10, height=4)
#grid.arrange(plot1,plot2, ncol = 2)
#dev.off()
grid.arrange(plot1,plot2, ncol = 2)
```

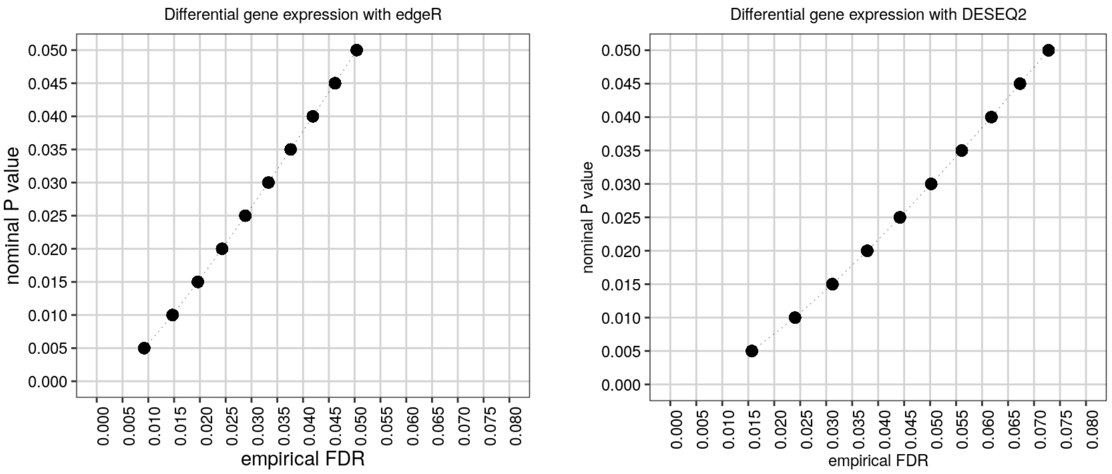

```
merged_res_not_preg_preg_AI_mRNA<-merge(res_not_preg_preg_AI_edgeR, res_not_preg_preg_AI_DeSeq, by.x="row.names", by.y="row")
merged_res_not_preg_preg_AI_mRNA<-merged_res_not_preg_preg_AI_mRNA[merged_res_not_preg_preg_AI_mRNA$PValue<=0.03
 & merged_res_not_preg_preg_AI_mRNA$pvalue<=0.03,]
merged_res_not_preg_preg_AI_mRNA<-merged_res_not_preg_preg_AI_mRNA[with(merged_res_not_preg_preg_AI_mRNA, order(logFC)), ]
#dim(merged_res_not_preg_preg_AI_mRNA) #353
#head(merged_res_not_preg_preg_AI_mRNA)
#write.table(merged_res_not_preg_preg_AI_mRNA, paste(result_path, "2019_04_30_merged_AI_NP_mRNA_before_LOOCV.txt"), quote=FALSE, sep="\t", row.names=FALSE)
DEG_AI_NP_mRNA<-merge(merged_res_not_preg_preg_AI_mRNA,annotation.ensembl.symbol, by.x="Row.names", by.y="ensembl_gene_id", all=FALSE)
DEG_AI_NP_mRNA<-DEG_AI_NP_mRNA[with(DEG_AI_NP_mRNA, order(logFC)), ]
is.num <- sapply(DEG_AI_NP_mRNA, is.numeric)
DEG_AI_NP_mRNA[is.num] <- lapply(DEG_AI_NP_mRNA[is.num], round, 4)
#write.table(DEG_AI_NP_mRNA, paste(result_path, "2019_12_26_merged_AI_NP_mRNA_gene_names_before_LOOCV.txt", sep="/"), quote=FALSE, sep="\t", row.names=FALSE)
```

```
merged_res_not_preg_preg_AI_mRNA_merged<-merge(res_not_preg_preg_AI_edgeR_merged, res_not_preg_preg_AI_DeSeq_merged, by.x="row.names", by.y="row")
merged_res_not_preg_preg_AI_mRNA_merged_a<-merged_res_not_preg_preg_AI_mRNA_merged[merged_res_not_preg_preg_AI_mRNA_merged$PValue<=0.03 & merged_res_not_preg_preg_AI_mRNA_merged$pvalue<=0.03,]
#write.table(merged_res_not_preg_preg_AI_mRNA, paste(result_path, "2019_04_22_merged_AI_NP_mRNA.txt"), quote=FALSE, sep="\t", row.names=FALSE)
DEG_AI_NP_mRNA_merged<-merge(merged_res_not_preg_preg_AI_mRNA_merged_a,annotation.ensembl.symbol, by.x="Row.names", by.y="ensembl_gene_id", all=FALSE)
DEG_AI_NP_mRNA_merged<-DEG_AI_NP_mRNA_merged[with(DEG_AI_NP_mRNA_merged, order(logFC)), ]
is.num <- sapply(DEG_AI_NP_mRNA_merged, is.numeric)
DEG_AI_NP_mRNA_merged[is.num] <- lapply(DEG_AI_NP_mRNA_merged[is.num], round, 4)
#write.table(DEG_AI_NP_mRNA_merged, paste(result_path, "2019_12_27_merged_AI_NP_mRNA_gene_names_both_years.txt",
 sep="/"), quote=FALSE, sep="\t", row.names=FALSE)
```

Plot the charts for all genes inferred as differentially expressed between AI and non pregnant. Not added as a supplementary material due to space.

```
cpm_AIpreg<-cpm_pwbc[,c(1,2,3,4,5,6)]
cpm_NOTpreg<-cpm_pwbc[,c(13,14,15,16,17)]
cpm_NBpreg<-cpm_pwbc[,c(7,8,9,10,11,12)]

data_chart_1<-data.frame( stringsAsFactors=FALSE)
data_chart_2<-data.frame( stringsAsFactors=FALSE)

for (i in  seq(dim(DEG_AI_NP_mRNA)[1])){

  gene_id<-DEG_AI_NP_mRNA[i,1]
  gene_symbol<-DEG_AI_NP_mRNA$external_gene_name[i]
  if(gene_symbol==""){gene_symbol<-gene_id}

  cpm_AI<-cpm_AIpreg[rownames(cpm_AIpreg)==gene_id,]
  cpm_NP<-cpm_NOTpreg[rownames(cpm_NOTpreg)==gene_id,]

  data_chart_1<-rbind(data.frame(cpm=cpm_AI), data.frame(cpm=cpm_NP))
  data_chart_1$group<-rep(c("Preg_AI","Not_Preg"),c(6,5))
  data_chart_1$shape<-as.factor(c(0:10))
  data_chart_1$gene<-gene_symbol
  data_chart_1$chart<-i
  data_chart_1$fold_change<-DEG_AI_NP_mRNA[DEG_AI_NP_mRNA$Row.names==gene_id,]$logFC
  data_chart_1$pvalue<-DEG_AI_NP_mRNA[DEG_AI_NP_mRNA$Row.names==gene_id,]$PValue

  data_chart_2<-rbind(data_chart_2,data_chart_1)
}

pdf(file=paste(result_path,"2019_12_27_plot_all_DEGs_preg_AI_not_preg_ordered_by_FC_before_LOOCV.pdf",sep="/"), w
idth= 11, paper="USr", bg="transparent", onefile=TRUE)

for (i in seq(1,dim(DEG_AI_NP_mRNA)[1], 24)) {
  plots <- list()
  k<-1
  for (j in c(i:(i+23))){
    data_chart_3<-data_chart_2[data_chart_2$chart %in% j , ]
    plot<-ggplot(data=data_chart_3, aes(x=group , y=cpm, colour=group))+
      geom_jitter(position = position_jitter(width = .1, height=0), aes( shape=shape), size=2)+
      scale_shape_manual(values=c(0:11))+
      scale_x_discrete(data_chart_3$gene[1],labels=c("Preg_AI"="Preg_AI", "Not_Preg"="Not_Preg"))+
      scale_colour_manual(name=NULL, values = c("#D55E00", "#0072B2"))+
      stat_summary(fun.y = median, fun.ymin = median, fun.ymax = median,colour = "gray", size = 0.2,  geom = "cro
ssbar", alpha=0.5)+
      scale_y_continuous("CPM")+
      ggtitle(paste("LogFC","=", round(data_chart_3$fold_change[1],1),"  ","P","=",round(data_chart_3$pvalue[1],4
), sep=c("")))+
      theme_bw()+
      theme(panel.grid= element_blank(),
            panel.background = element_blank(),
            panel.grid.minor = element_blank(),
            panel.grid.major = element_blank(),
            plot.background = element_blank(),
            strip.background = element_rect(fill = "white"),
            #strip.text.x = element_text(colour = 'black', face="italic",size = 9),
            #legend.text = element_text( colour = 'black', size = 10 ),
            axis.text.x = element_blank(),
            axis.text.y = element_text( colour = 'black', size = 8 ),
            axis.title = element_text( colour = 'black', size = 9 ),
            legend.key.size = unit(0.9,"cm"),
            legend.position="none",
            axis.ticks.x = element_blank(),
            plot.title = element_text(size = 7)
      )

    plots[[k]] <- plot
    k<-k+1
  }
  multiplot(plotlist = plots, cols = 6,layout = matrix(1:24, nrow=4, byrow = TRUE))
}
dev.off()
```

Plot the charts for all genes inferred as differentially expressed between AI and non pregnant for merged dataset. Not added as a supplementary material due to space.

```
cpm_AIpreg<-cpm_merged_datasets[,c(1,2,3,4,5,6,18,22,23,24,25,27)]
cpm_NOTpreg<-cpm_merged_datasets[,c(13,14,15,16,17,19,20,21,26,28,29)]


data_chart_1<-data.frame( stringsAsFactors=FALSE)
data_chart_2<-data.frame( stringsAsFactors=FALSE)

for (i in  seq(dim(DEG_AI_NP_mRNA_merged)[1])){

  gene_id<-DEG_AI_NP_mRNA_merged[i,1]
  gene_symbol<-DEG_AI_NP_mRNA_merged$external_gene_name[i]
  if(gene_symbol==""){gene_symbol<-gene_id}

  cpm_AI<-cpm_AIpreg[rownames(cpm_AIpreg)==gene_id,]
  cpm_NP<-cpm_NOTpreg[rownames(cpm_NOTpreg)==gene_id,]

  data_chart_1<-rbind(data.frame(cpm=cpm_AI), data.frame(cpm=cpm_NP))
  data_chart_1$group<-rep(c("Preg_AI","Not_Preg"),c(12,11))
  data_chart_1$shape<-as.factor(c(0:22))
  data_chart_1$gene<-gene_symbol
  data_chart_1$chart<-i
  data_chart_1$fold_change<-DEG_AI_NP_mRNA_merged[DEG_AI_NP_mRNA_merged$Row.names==gene_id,]$logFC
  data_chart_1$pvalue<-DEG_AI_NP_mRNA_merged[DEG_AI_NP_mRNA_merged$Row.names==gene_id,]$PValue


  data_chart_2<-rbind(data_chart_2,data_chart_1)
}

pdf(file=paste(result_path,"2019_12_27_plot_all_DEGs_preg_AI_not_preg_merged.pdf",sep="/"), width= 11, paper="US
r", bg="transparent", onefile=TRUE)

for (i in seq(1,dim(DEG_AI_NP_mRNA_merged)[1], 24)) {
  plots <- list()
  k<-1
  for (j in c(i:(i+23))){
    data_chart_3<-data_chart_2[data_chart_2$chart %in% j , ]
    plot<-ggplot(data=data_chart_3, aes(x=group , y=cpm, colour=group))+
      geom_jitter(position = position_jitter(width = .1, height=0), aes( shape=shape), size=2)+
      scale_shape_manual(values=c(0:22))+
      scale_x_discrete(data_chart_3$gene[1],labels=c("Preg_AI"="Preg_AI", "Not_Preg"="Not_Preg"))+
      scale_colour_manual(name=NULL, values = c("#D55E00", "#0072B2"))+
      stat_summary(fun.y = median, fun.ymin = median, fun.ymax = median,colour = "gray", size = 0.2,  geom = "cro
ssbar", alpha=0.5)+
      scale_y_continuous("CPM")+
      ggtitle(paste("LogFC","=", round(data_chart_3$fold_change[1],1),"  ","P","=",round(data_chart_3$pvalue[1],4
), sep=c("")))+
      theme_bw()+
      theme(panel.grid= element_blank(),
            panel.background = element_blank(),
            panel.grid.minor = element_blank(),
            panel.grid.major = element_blank(),
            plot.background = element_blank(),
            strip.background = element_rect(fill = "white"),
            #strip.text.x = element_text(colour = 'black', face="italic",size = 9),
            #legend.text = element_text( colour = 'black', size = 10 ),
            axis.text.x = element_blank(),
            axis.text.y = element_text( colour = 'black', size = 8 ),
            axis.title = element_text( colour = 'black', size = 9 ),
            legend.key.size = unit(0.9,"cm"),
            legend.position="none",
            axis.ticks.x = element_blank(),
            plot.title = element_text(size = 7)
      )

    plots[[k]] <- plot
    k<-k+1
  }
  multiplot(plotlist = plots, cols = 6,layout = matrix(1:24, nrow=4, byrow = TRUE))
}
dev.off()
```

Supplementary table 11

Leave-one-out cross validation.

```
cl <- makeCluster(12)
registerDoParallel(cl)

group<-factor(c("Preg_AI","Preg_AI","Preg_AI","Preg_AI","Preg_AI","Preg_AI","Preg_NB","Preg_NB","Preg_NB","Preg_N
B","Preg_NB","Preg_NB","Not_Preg","Not_Preg","Not_Preg","Not_Preg","Not_Preg"), levels=c("Preg_AI","Preg_NB", "No
t_Preg"))

out_list_IDs_LOOCV<-foreach(i = c(1:6,13:17), .inorder=FALSE,.packages=c("edgeR","DESeq2"), .verbose=FALSE ) %dop
ar% {

group_a<-group[-i]
count_pwbc_a<-count_pwbc[,-i]

design<-model.matrix(~group_a)
dds<-DGEList(count=count_pwbc_a, group=group_a)
dds<-estimateDisp(dds, design, robust=TRUE)
dds<-glmFit(dds, design)
dds<-glmLRT(dds,coef="group_aNot_Preg")
res_not_preg_preg_AI_edgeR<-topTags(dds,n=Inf)$table

#DESeq
colData<-data.frame("group"=group_a)
rownames(colData)<-colnames(count_pwbc_a)
dds<-DESeqDataSetFromMatrix(countData=count_pwbc_a, colData=colData, design= ~group)
dds<-DESeq(dds)
res_not_preg_preg_AI_DeSeq<-results(dds, contrast=c("group","Not_Preg" ,"Preg_AI"), pAdjustMethod="fdr", tidy=TRU
E)


merged_res_not_preg_preg_AI_mRNA_a<-merge(res_not_preg_preg_AI_edgeR, res_not_preg_preg_AI_DeSeq, by.x="row.name
s", by.y="row")
merged_res_not_preg_preg_AI_mRNA_a<-merged_res_not_preg_preg_AI_mRNA_a[merged_res_not_preg_preg_AI_mRNA_a$PValue<
=0.03 & merged_res_not_preg_preg_AI_mRNA_a$pvalue<=0.03,]

rm(group_a,count_pwbc_a)

merged_res_not_preg_preg_AI_mRNA_a$Row.names
}

stopCluster(cl)

overlapping_genes_LOOCV<-Reduce(intersect, out_list_IDs_LOOCV)

merged_res_not_preg_preg_AI_mRNA<-merge(res_not_preg_preg_AI_edgeR, res_not_preg_preg_AI_DeSeq, by.x="row.names",
by.y="row")
merged_res_not_preg_preg_AI_mRNA_a<-merged_res_not_preg_preg_AI_mRNA[merged_res_not_preg_preg_AI_mRNA$Row.names %
in% overlapping_genes_LOOCV,]
DEG_AI_NP_mRNA<-merge(merged_res_not_preg_preg_AI_mRNA_a,annotation.ensembl.symbol, by.x="Row.names", by.y="ensem
bl_gene_id", all=FALSE)
DEG_AI_NP_mRNA<-DEG_AI_NP_mRNA[with(DEG_AI_NP_mRNA, order(logFC)), ]
is.num <- sapply(DEG_AI_NP_mRNA, is.numeric)
DEG_AI_NP_mRNA[is.num] <- lapply(DEG_AI_NP_mRNA[is.num], round, 4)
#dim(DEG_AI_NP_mRNA)
#head(DEG_AI_NP_mRNA)
#write.table(DEG_AI_NP_mRNA, paste(result_path, "2019_12_27_merged_AI_NP_mRNA_gene_names_after_LOOCV.txt",sep
="/"), quote=FALSE, sep="\t", row.names=FALSE)
rm(out_list_IDs_LOOCV)
```

```
knitr::kable(head(DEG_AI_NP_mRNA), caption = "First five rows of Supplementary Table 11",format = 'pandoc')
```

First five rows of Supplementary Table 11

	Row.names	logFC	logCPM	LR	PValue	FDR	baseMean	log2FoldChange	lfcSE	stat	pvalue	padj	external_gene_n
64	ENSBTAG00000052118	-4.4422	0.1925	8.1231	0.0044	0.6588	17.1086	-4.5092	1.5625	-2.8860	0.0039	0.2636	
49	ENSBTAG00000034281	-3.0239	1.0672	7.2748	0.0070	0.6588	36.8808	-2.8357	1.0866	-2.6098	0.0091	0.3176	MGAT4D
66	ENSBTAG00000052658	-2.2694	4.0733	11.1769	0.0008	0.6467	291.2199	-1.7625	0.6162	-2.8603	0.0042	0.2636	NKG2A
62	ENSBTAG00000051183	-2.1635	4.1301	13.4758	0.0002	0.3624	308.3543	-1.9229	0.5047	-3.8097	0.0001	0.1073	
14	ENSBTAG00000007247	-2.1530	4.7636	8.3500	0.0039	0.6588	471.4847	-2.0381	0.6571	-3.1018	0.0019	0.2122	NUF2

	Row.names	logFC	logCPM	LR	PValue	FDR	baseMean	log2FoldChange	lfcSE	stat	pvalue	padj	external_gene_n
63	ENSBTAG00000051500	-2.1186	2.2460	6.7875	0.0092	0.6588	81.1065	-2.0127	0.7156	-2.8127	0.0049	0.2676	ZNF389

Plot the charts for all genes inferred as differentially expressed between AI and non pregnant. Not added to the supplementary material due do space.

```
cpm_AIpreg<-cpm_pwbc[,c(1,2,3,4,5,6)]
cpm_NOTpreg<-cpm_pwbc[,c(13,14,15,16,17)]
cpm_NBpreg<-cpm_pwbc[,c(7,8,9,10,11,12)]

data_chart_1<-data.frame( stringsAsFactors=FALSE)
data_chart_2<-data.frame( stringsAsFactors=FALSE)

for (i in  seq(dim(DEG_AI_NP_mRNA)[1])){

  gene_id<-DEG_AI_NP_mRNA[i,1]
  gene_symbol<-DEG_AI_NP_mRNA$external_gene_name[i]
  if(gene_symbol==""){gene_symbol<-gene_id}

  cpm_AI<-cpm_AIpreg[rownames(cpm_AIpreg)==gene_id,]
  cpm_NP<-cpm_NOTpreg[rownames(cpm_NOTpreg)==gene_id,]

  data_chart_1<-rbind(data.frame(cpm=cpm_AI), data.frame(cpm=cpm_NP))
  data_chart_1$group<-rep(c("Preg_AI","Not_Preg"),c(6,5))
  data_chart_1$shape<-as.factor(c(0:10))
  data_chart_1$gene<-gene_symbol
  data_chart_1$chart<-i
  data_chart_1$fold_change<-DEG_AI_NP_mRNA[DEG_AI_NP_mRNA$Row.names==gene_id,]$logFC
  data_chart_1$pvalue<-DEG_AI_NP_mRNA[DEG_AI_NP_mRNA$Row.names==gene_id,]$PValue


  data_chart_2<-rbind(data_chart_2,data_chart_1)
}

pdf(file=paste(result_path,"2019_12_27_plot_all_DEGs_preg_AI_not_preg_ordered_by_FC_after_LOOCV.pdf",sep="/"), wi
dth= 11, paper="USr", bg="transparent", onefile=TRUE)


for (i in seq(1,dim(DEG_AI_NP_mRNA)[1], 24)) {
  plots <- list()
  k<-1
  for (j in c(i:(i+23))){
    data_chart_3<-data_chart_2[data_chart_2$chart %in% j , ]
    plot<-ggplot(data=data_chart_3, aes(x=group , y=cpm, colour=group))+
      geom_jitter(position = position_jitter(width = .1, height=0), aes( shape=shape), size=2)+
      scale_shape_manual(values=c(0:11))+
      scale_x_discrete(data_chart_3$gene[1],labels=c("Preg_AI"="Preg_AI", "Not_Preg"="Not_Preg"))+
      scale_colour_manual(name=NULL, values = c("#D55E00", "#0072B2"))+
      stat_summary(fun.y = median, fun.ymin = median, fun.ymax = median,colour = "gray", size = 0.2,  geom = "cro
ssbar", alpha=0.5)+
      scale_y_continuous("CPM")+
      ggtitle(paste("LogFC","=", round(data_chart_3$fold_change[1],1),"  ","P","=",round(data_chart_3$pvalue[1],4
), sep=c("")))+
      theme_bw()+
      theme(panel.grid= element_blank(),
            panel.background = element_blank(),
            panel.grid.minor = element_blank(),
            panel.grid.major = element_blank(),
            plot.background = element_blank(),
            strip.background = element_rect(fill = "white"),
            #strip.text.x = element_text(colour = 'black', face="italic",size = 9),
            #legend.text = element_text( colour = 'black', size = 10 ),
            axis.text.x = element_blank(),
            axis.text.y = element_text( colour = 'black', size = 8 ),
            axis.title = element_text( colour = 'black', size = 9 ),
            legend.key.size = unit(0.9,"cm"),
            legend.position="none",
            axis.ticks.x = element_blank(),
            plot.title = element_text(size = 7)
      )

    plots[[k]] <- plot
    k<-k+1
  }
  multiplot(plotlist = plots, cols = 6,layout = matrix(1:24, nrow=4, byrow = TRUE))
}
dev.off()
```

Create the background gene lists for gene ontology.

```
all_genes<-data.frame( gene=rownames(count_pwbc), stringsAsFactors=FALSE )
rownames(all_genes)<-all_genes$gene

N_expressed_genes<-length(all_genes$gene)

annotation.genelength.biomart_vector<-gene_length[,2]
names(annotation.genelength.biomart_vector)<-gene_length[,1]

annotation.GO.BP.biomart<-annotation.GO.biomart[annotation.GO.biomart$namespace_1003=="biological_process", c(1,3
)]
annotation.GO.BP.biomart<-annotation.GO.BP.biomart[annotation.GO.BP.biomart$ensembl_gene_id %in% rownames(all_gen
es),]
annotation.GO.MF.biomart<-annotation.GO.biomart[annotation.GO.biomart$namespace_1003=="molecular_function", c(1,3
)]
annotation.GO.MF.biomart<-annotation.GO.MF.biomart[annotation.GO.MF.biomart$ensembl_gene_id %in% rownames(all_gen
es),]
```

Test biological processes for enrichment with DEGs and calculate fold change enrichment. (AI Pregnant vs non pregnant)

```
test.genes<-data.frame(a=unique(c(as.character(merged_res_not_preg_preg_AI_mRNA_a$Row.names))), stringsAsFactors=
FALSE)
all_genes_numeric<-as.integer(all_genes$gene %in%test.genes$a)
names(all_genes_numeric)<-all_genes$gene

N_DEGs<-length(test.genes$a)

set.seed(9830)
pwf<-nullp(all_genes_numeric, bias.data=annotation.genelength.biomart_vector, plot.fit=FALSE )
GO_BP_Cats_mRNA_AI_NP<-goseq(pwf,gene2cat=annotation.GO.BP.biomart, method ="Sampling", repcnt = 10000, use_genes
_without_cat=FALSE)
GO_BP_Cats_mRNA_AI_NP<-GO_BP_Cats_mRNA_AI_NP[GO_BP_Cats_mRNA_AI_NP$numDEInCat>4,]
GO_BP_Cats_mRNA_AI_NP$BY_FDR<-p.adjust(GO_BP_Cats_mRNA_AI_NP$over_represented_pvalue, method ="fdr")
GO_BP_Cats_mRNA_AI_NP<-GO_BP_Cats_mRNA_AI_NP[with(GO_BP_Cats_mRNA_AI_NP, order(BY_FDR,over_represented_pvalue, -n
umDEInCat)), ]
#head(GO_BP_Cats_mRNA_AI_NP, n=20)

GO_BP_Cats_mRNA_AI_NP$fold_enrichment<-(GO_BP_Cats_mRNA_AI_NP$numDEInCat/N_DEGs)/(GO_BP_Cats_mRNA_AI_NP$numInCat/
N_expressed_genes)
annotation.GO.BP.biomart_testgenes<-annotation.GO.BP.biomart[annotation.GO.BP.biomart$ensembl_gene_id %in% test.g
enes$a, ]
GO_BP_Cats_mRNA_AI_NP<-merge(GO_BP_Cats_mRNA_AI_NP,annotation.GO.BP.biomart_testgenes, by.x="category", by.y="go_
id", all.x=TRUE, all.y=FALSE)
GO_BP_Cats_mRNA_AI_NP<-merge(GO_BP_Cats_mRNA_AI_NP, annotation.ensembl.symbol, by.x="ensembl_gene_id", by.y="ense
mbl_gene_id", all=FALSE, all.x=TRUE, all.y=FALSE)
GO_BP_Cats_mRNA_AI_NP<-GO_BP_Cats_mRNA_AI_NP[GO_BP_Cats_mRNA_AI_NP$BY_FDR<0.1,]
GO_BP_Cats_mRNA_AI_NP<-GO_BP_Cats_mRNA_AI_NP[with(GO_BP_Cats_mRNA_AI_NP, order(BY_FDR,term)), ]
#write.table(GO_BP_Cats_mRNA_AI_NP, paste(result_path, "2019_12_27_GO_BP_Cats_DEG_mRNA_AI_NP.txt",sep="/"), quote
=FALSE, sep="\t", row.names=FALSE)
```

```
knitr::kable(head(GO_BP_Cats_mRNA_AI_NP), caption = "First five rows GO-BP analysis DEGs",format = 'pandoc')
```

First five rows GO-BP analysis DEGs

ensembl_gene_id	category	over_represented_pvalue	under_represented_pvalue	numDEInCat	numInCat	term	ontology	BY_FD
ENSBTAG00000005018	GO:0007165 (GO:0007165)	0.0457954	0.9853015	5	348	signal transduction	BP	0.045795
ENSBTAG00000010447	GO:0007165 (GO:0007165)	0.0457954	0.9853015	5	348	signal transduction	BP	0.045795
ENSBTAG00000012500	GO:0007165 (GO:0007165)	0.0457954	0.9853015	5	348	signal transduction	BP	0.045795
ENSBTAG00000015938	GO:0007165 (GO:0007165)	0.0457954	0.9853015	5	348	signal transduction	BP	0.045795

ensembl_gene_id	category	over_represented_pvalue	under_represented_pvalue	numDEInCat	numInCat	term	ontology	BY_FD
ENSBTAG00000040188	GO:0007165 (GO:0007165)	0.0457954	0.9853015	5	348	signal transduction	BP	0.045795

Test molecular functions for enrichment with DEGs and calculate fold change enrichment. (AI Pregnant vs non pregnant)

```
set.seed(9830)
pwf<-nullp(all_genes_numeric, bias.data=annotation.genelength.biomart_vector, plot.fit=FALSE )
GO_MF_Cats_mRNA_AI_NP<-goseq(pwf,gene2cat=annotation.GO.MF.biomart, method ="Sampling", repcnt = 10000, use_genes
_without_cat=FALSE)
GO_MF_Cats_mRNA_AI_NP<-GO_MF_Cats_mRNA_AI_NP[GO_MF_Cats_mRNA_AI_NP$numDEInCat>4,]
GO_MF_Cats_mRNA_AI_NP$BY_FDR<-p.adjust(GO_MF_Cats_mRNA_AI_NP$over_represented_pvalue, method ="fdr")
GO_MF_Cats_mRNA_AI_NP<-GO_MF_Cats_mRNA_AI_NP[with(GO_MF_Cats_mRNA_AI_NP, order(BY_FDR,over_represented_pvalue, -n
umDEInCat)), ]
#head(GO_MF_Cats_mRNA_AI_NP, n=20)

GO_MF_Cats_mRNA_AI_NP$fold_enrichment<-(GO_MF_Cats_mRNA_AI_NP$numDEInCat/N_DEGs)/(GO_MF_Cats_mRNA_AI_NP$numInCat/
N_expressed_genes)
annotation.GO.MF.biomart_testgenes<-annotation.GO.MF.biomart[annotation.GO.MF.biomart$ensembl_gene_id %in% test.g
enes$a, ]
GO_MF_Cats_mRNA_AI_NP<-merge(GO_MF_Cats_mRNA_AI_NP,annotation.GO.MF.biomart_testgenes, by.x="category", by.y="go_
id", all.x=TRUE, all.y=FALSE)
GO_MF_Cats_mRNA_AI_NP<-merge(GO_MF_Cats_mRNA_AI_NP, annotation.ensembl.symbol, by.x="ensembl_gene_id", by.y="ense
mbl_gene_id", all=FALSE, all.x=TRUE, all.y=FALSE)
GO_MF_Cats_mRNA_AI_NP<-GO_MF_Cats_mRNA_AI_NP[GO_MF_Cats_mRNA_AI_NP$BY_FDR<0.1,]
GO_MF_Cats_mRNA_AI_NP<-GO_MF_Cats_mRNA_AI_NP[with(GO_MF_Cats_mRNA_AI_NP, order(BY_FDR,term)), ]
#write.table(GO_MF_Cats_mRNA_AI_NP, paste(result_path, "2019_10_10_GO_MF_Cats_mRNA_AI_NP.txt",sep="/"), quote=FAL
SE, sep="\t", row.names=FALSE)
```

Pathway analysis through KEGG Pathways. (AI Pregnant vs non pregnant)

```
all_genes_entrez<- stack(mget(all_genes$gene, org.Bt.egENSEMBL2EG, ifnotfound = NA))
all_genes_entrez<-all_genes_entrez[complete.cases(all_genes_entrez),]
all_genes_entrez<-all_genes_entrez[!duplicated(all_genes_entrez$values),]

test.genes_entrez <- stack(mget(test.genes$a, org.Bt.egENSEMBL2EG, ifnotfound = NA))
test.genes_entrez<-test.genes_entrez[complete.cases(test.genes_entrez),]

all_genes_entrez_numeric<-as.integer(all_genes_entrez$values %in% test.genes_entrez$values)
names(all_genes_entrez_numeric)<-all_genes_entrez$values

entrez_kegg<- stack(mget(all_genes_entrez$values, org.Bt.egPATH, ifnotfound = NA))
entrez_kegg<-entrez_kegg[complete.cases(entrez_kegg),]
entrez_kegg<-entrez_kegg[,c(2,1)]
entrez_kegg_test_genes<-entrez_kegg[entrez_kegg$ind %in% test.genes_entrez$values,]


pwf<-nullp(all_genes_entrez_numeric, 'bosTau4','refGene', plot.fit=FALSE )

set.seed(8503)
kegg_AI_NP_subset<-goseq(pwf, gene2cat=entrez_kegg,method ="Sampling", repcnt = 10000, use_genes_without_cat=FALS
E)
kegg_AI_NP_subset<-kegg_AI_NP_subset[kegg_AI_NP_subset$numDEInCat>4,]
kegg_AI_NP_subset$BY_FDR<-p.adjust(kegg_AI_NP_subset$over_represented_pvalue, method ="fdr")
kegg_AI_NP_subset<-merge(kegg_AI_NP_subset,entrez_kegg_test_genes, by.x="category", by.y="values", all.x=TRUE, al
l.y=FALSE)
kegg_AI_NP_subset<-merge(kegg_AI_NP_subset,all_genes_entrez, by.x="ind", by.y="values", all.x=TRUE, all.y=FALSE,
 suffixes =c('.entrez','.ensembl'))
kegg_AI_NP_subset<-kegg_AI_NP_subset[with(kegg_AI_NP_subset, order(BY_FDR, -numDEInCat)), ]
kegg_AI_NP_subset<-kegg_AI_NP_subset[,c(2:8)]
kegg_AI_NP_subset<-merge(kegg_AI_NP_subset, annotation.ensembl.symbol, by.x="ind.ensembl", by.y="ensembl_gene_id"
, all.x=TRUE, all.y=FALSE)
kegg_AI_NP_subset<-kegg_AI_NP_subset[with(kegg_AI_NP_subset, order(BY_FDR, -numDEInCat)), ]
kegg_AI_NP_subset<-kegg_AI_NP_subset[kegg_AI_NP_subset$BY_FDR<0.1,]
```

DEG Analysis for mRNA

AI Pregnant vs Pregnant to Natural Service

Results from edgeR

```
#colnames(count_pwbc)
group<-factor(c("Preg_AI","Preg_AI","Preg_AI","Preg_AI","Preg_AI","Preg_AI","Preg_NB","Preg_NB","Preg_NB","Preg_N
B","Preg_NB","Preg_NB","Not_Preg","Not_Preg","Not_Preg","Not_Preg","Not_Preg"), levels=c("Preg_AI","Preg_NB", "No
t_Preg"))
design<-model.matrix(~group)
dds<-DGEList(count=count_pwbc, group=group)
dds<-estimateDisp(dds, design, robust=TRUE)
dds<-glmFit(dds, design)
dds<-glmLRT(dds,coef="groupPreg_NB")
res_preg_AI_preg_NB_mRNA_edgeR<-topTags(dds,n=Inf)$table
#head(res_preg_AI_preg_NB_mRNA_edgeR,n=30)
#write.table(res_preg_AI_preg_NB_mRNA_edgeR, paste(result_path, "2019_04_30_res_preg_AI_preg_NB_mRNA_edgeR.txt"),
quote=FALSE, sep="\t", row.names=FALSE)
```

Results from DESeq

```
group<-factor(c("Preg_AI","Preg_AI","Preg_AI","Preg_AI","Preg_AI","Preg_AI","Preg_NB","Preg_NB","Preg_NB","Preg_N
B","Preg_NB","Preg_NB","Not_Preg","Not_Preg","Not_Preg","Not_Preg","Not_Preg"), levels=c("Preg_AI","Preg_NB", "No
t_Preg"))
design<-model.matrix(~group)
colData<-data.frame("group"=group)
rownames(colData)<-colnames(count_pwbc)
dds<-DESeqDataSetFromMatrix(countData=count_pwbc, colData=colData, design= ~group)
dds<-DESeq(dds)
res_preg_AI_preg_NB_mRNA_DeSeq<-results(dds, contrast=c("group","Preg_NB" ,"Preg_AI"), pAdjustMethod="fdr", tidy=
TRUE)
res_preg_AI_preg_NB_mRNA_DeSeq<-res_preg_AI_preg_NB_mRNA_DeSeq[with(res_preg_AI_preg_NB_mRNA_DeSeq, order(pvalu
e)), ]
#head(res_preg_AI_preg_NB_mRNA_DeSeq,n=30)
#write.table(res_preg_AI_preg_NB_mRNA_DeSeq, paste(result_path, "2019_04_30_res_preg_AI_preg_NB_mRNA_DeSeq.txt",
 sep="/"), quote=FALSE, sep="\t", row.names=FALSE)
```

Calculation of the eFDR for mRNA comparisons

```
#Create the permutations matrix
perm_without_replacement <- function(n, r){
  return(factorial(n)/factorial(n - r))
}

permutation_matrix<-matrix(,nrow=200100,ncol=17)
head(permutation_matrix)
dim(permutation_matrix)

for (i in seq(1:200100)){
  sampling <- sample(1:17,17, replace = FALSE)
  if ( ! identical(sampling , c(1:17))){
    permutation_matrix[i,]<-sample(1:17,17, replace = FALSE)
  }}

permutation_matrix<-permutation_matrix[!duplicated(permutation_matrix),]
dim(permutation_matrix) #number changes each time
head(permutation_matrix)

permutation_matrix<-permutation_matrix[sample(1:dim(permutation_matrix)[1], 10000),]   #should this number for th
e dim of the earlier
rand<-dim(permutation_matrix)[1]

sequence.pvalue<-seq(0.005, 0.05, 0.005)

#edgeR
results <- filebacked.big.matrix(length(sequence.pvalue),rand, type="double", init=0, separated=FALSE,
                                 backingfile="incidence_matrix.bin",
                                 descriptor="incidence_matrix.desc")  #this does not overwrite, create once and d
elete if want to create again
mdesc_result<- describe(results)

#edgeR
cl <- makeCluster(10)
registerDoParallel(cl)

results[,]<-foreach(i = sequence.pvalue, .combine='rbind', .inorder=TRUE,  .packages=c("edgeR","bigmemory"), .ver
bose=FALSE) %:%

  foreach(j = 1:rand, .combine='cbind', .inorder=FALSE,.packages=c("edgeR","bigmemory"), .verbose=FALSE ) %dopar%
{

    group<-factor(c("Preg_AI","Preg_AI","Preg_AI","Preg_AI","Preg_AI","Preg_AI","Preg_NB","Preg_NB","Preg_NB","Pr
eg_NB","Preg_NB","Preg_NB","Not_Preg","Not_Preg","Not_Preg","Not_Preg","Not_Preg"), levels=c("Preg_AI","Preg_NB",
"Not_Preg"))
    group<-group[permutation_matrix[j,]]
    group<-factor(group, levels=c("Preg_AI","Not_Preg", "Preg_NB"))
    design <- model.matrix(~group)
    dds<-DGEList(count=count_pwbc, group=group)
    dds<-estimateDisp(dds, design, robust=TRUE)
    dds <- glmFit(dds, design)
    dds <- glmLRT(dds, coef="groupPreg_NB")
    length(which(topTags(dds,n=Inf)$table$PValue < i))

  }

stopCluster(cl)

results[1:5,1:5]

total.rand <- rand * 10496

qvalue<-data.frame(raw.pvalue = sequence.pvalue,
                   e.pvalue.edgeR= (rowSums(results[,]+1))/(total.rand+1),
                   e.pvalue.edgeR.round= round((rowSums(results[,]+1))/(total.rand+1) ,4))

qvalue

rm(results)
system("rm incidence_matrix.bin")
system("rm incidence_matrix.desc")

#DESEQ2

results.rand <- filebacked.big.matrix(length(sequence.pvalue),rand, type="double", init=0, separated=FALSE,
                                      backingfile="incidence_matrix.bin",
                                      descriptor="incidence_matrix.desc")
mdesc_result<- describe(results.rand)

cl <- makeCluster(10)
registerDoParallel(cl)
```

```
results.rand[,]<-foreach(i = sequence.pvalue, .combine='rbind', .inorder=TRUE,  .packages=c("DESeq2","bigmemory"
), .verbose=TRUE) %:%

  foreach(j = 1:rand, .combine='cbind', .inorder=FALSE,.packages=c("DESeq2","bigmemory"), .verbose=TRUE ) %dopar%
{

    group<- c("Preg_AI","Preg_AI","Preg_AI","Preg_AI","Preg_AI","Preg_AI","Preg_NB","Preg_NB","Preg_NB","Preg_NB"
,"Preg_NB","Preg_NB","Not_Preg","Not_Preg","Not_Preg","Not_Preg","Not_Preg")
    group<-group[permutation_matrix[j,]]
    group<-factor(group, levels=c("Preg_AI","Not_Preg", "Preg_NB"))
    design <- model.matrix(~group)

    colData<-data.frame("group"=group)
    rownames(colData)<-colnames(count_pwbc)
    dds<-DESeqDataSetFromMatrix(countData=count_pwbc, colData=colData, design= ~group)
    dds <- DESeq(dds)
    DeSeq_AI_NP <- results(dds, contrast=c("group","Preg_NB" ,"Preg_AI"), pAdjustMethod="fdr", tidy=TRUE)

    length(which(DeSeq_AI_NP$pvalue < i))

  }

stopCluster(cl)

results.rand[1:5,1:5]

total.rand <- rand * 10496


qvalue2<-data.frame(raw.pvalue = sequence.pvalue,
                    e.pvalue.DESEQ2= (rowSums(results.rand[,]+1))/(total.rand+1),
                    e.pvalue.DESEQ2.round= round((rowSums(results.rand[,]+1))/(total.rand+1) ,4))

qvalue2

qvalue3<-cbind(qvalue,qvalue2)

qvalue3

rm(results.rand)
system("rm incidence_matrix.bin")
system("rm incidence_matrix.desc")

write.table(qvalue3,paste(result_path,"2019_12_27_empirical_FDR_AI_NB.txt",sep="/"), quote=FALSE, sep="\t", row.n
ames=FALSE)
```

Merge the results for edgeR and DESeq. (AI Pregnant vs Pregnant to Natural Service)

```
merged_res_preg_AI_preg_NB_mRNA<-merge(res_preg_AI_preg_NB_mRNA_edgeR, res_preg_AI_preg_NB_mRNA_DeSeq, by.x="row.
names", by.y="row")
merged_res_preg_AI_preg_NB_mRNA<-merged_res_preg_AI_preg_NB_mRNA[merged_res_preg_AI_preg_NB_mRNA$PValue<=0.03 & m
erged_res_preg_AI_preg_NB_mRNA$pvalue<=0.03,]
#dim(merged_res_preg_AI_preg_NB_mRNA) #12
merged_res_preg_AI_preg_NB_mRNA<-merged_res_preg_AI_preg_NB_mRNA[with(merged_res_preg_AI_preg_NB_mRNA, order(logF
C)), ]
#head(merged_res_preg_AI_preg_NB_mRNA)
DEG_AI_NB_mRNA<-merge(merged_res_preg_AI_preg_NB_mRNA,annotation.ensembl.symbol, by.x="Row.names", by.y="ensembl_
gene_id", all=FALSE)
DEG_AI_NB_mRNA<-DEG_AI_NB_mRNA[with(DEG_AI_NB_mRNA, order(logFC)), ]
#write.table(merged_res_preg_AI_preg_NB_mRNA, paste(result_path, "2019_04_30_merged_res_preg_AI_preg_NB_mRNA_befo
re_LOOCV.txt"), quote=FALSE, sep="\t", row.names=FALSE)
#write.table(DEG_AI_NB_mRNA, paste(result_path, "2019_12_27_merged_AI_NB_mRNA_gene_names_before_LOOCV.txt", sep
="/"), quote=FALSE, sep="\t", row.names=FALSE)
```

Plot the charts for all genes inferred as differentially expressed between AI and NB pregnant

```
cpm_AIpreg<-cpm_pwbc[,c(1,2,3,4,5,6)]
cpm_NOTpreg<-cpm_pwbc[,c(13,14,15,16,17)]
cpm_NBpreg<-cpm_pwbc[,c(7,8,9,10,11,12)]

data_chart_1<-data.frame( stringsAsFactors=FALSE)
data_chart_2<-data.frame( stringsAsFactors=FALSE)

for (i in  seq(dim(DEG_AI_NB_mRNA)[1])){

  gene_id<-DEG_AI_NB_mRNA[i,1]
  gene_symbol<-DEG_AI_NB_mRNA$external_gene_name[i]
  if(gene_symbol==""){gene_symbol<-gene_id}

  cpm_AI<-cpm_AIpreg[rownames(cpm_AIpreg)==gene_id,]
  cpm_NB<-cpm_NBpreg[rownames(cpm_NBpreg)==gene_id,]

  data_chart_1<-rbind(data.frame(cpm=cpm_AI), data.frame(cpm=cpm_NB))
  data_chart_1$group<-rep(c("Preg_AI","Preg_NB"),c(6,6))
  data_chart_1$shape<-as.factor(c(0:11))
  data_chart_1$gene<-gene_symbol
  data_chart_1$chart<-i
  data_chart_1$fold_change<-DEG_AI_NB_mRNA[DEG_AI_NB_mRNA$Row.names==gene_id,]$logFC
  data_chart_1$pvalue<-DEG_AI_NB_mRNA[DEG_AI_NB_mRNA$Row.names==gene_id,]$PValue


  data_chart_2<-rbind(data_chart_2,data_chart_1)
}

pdf(file=paste(result_path,"2019_12_27_plot_all_DEGs_preg_AI_preg_NB_ordered_by_FC_before_LOOCV.pdf",sep="/"), wi
dth= 11, height=4, bg="transparent", onefile=TRUE)

for (i in seq(1,dim(DEG_AI_NB_mRNA)[1], 24)) {
  plots <- list()
  k<-1
  for (j in c(i:(i+11))){
    data_chart_3<-data_chart_2[data_chart_2$chart %in% j , ]
    plot<-ggplot(data=data_chart_3, aes(x=group , y=cpm, colour=group))+
      geom_jitter(position = position_jitter(width = .1, height=0), aes( shape=shape), size=2)+
      scale_shape_manual(values=c(0:11))+
      scale_x_discrete(data_chart_3$gene[1],labels=c("Preg_AI"="Preg_AI", "Preg_NB"="Preg_NB"))+
      scale_colour_manual(name=NULL, values = c("#0072B2", "#000000"))+
      stat_summary(fun.y = median, fun.ymin = median, fun.ymax = median,colour = "gray", size = 0.2,  geom = "cro
ssbar", alpha=0.5)+
      scale_y_continuous("CPM")+
      ggtitle(paste("LogFC","=", round(data_chart_3$fold_change[1],1),"  ","P","=",round(data_chart_3$pvalue[1],4
), sep=c("")))+
      theme_bw()+
      theme(panel.grid= element_blank(),
            panel.background = element_blank(),
            panel.grid.minor = element_blank(),
            panel.grid.major = element_blank(),
            plot.background = element_blank(),
            strip.background = element_rect(fill = "white"),
            #strip.text.x = element_text(colour = 'black', face="italic",size = 9),
            #legend.text = element_text( colour = 'black', size = 10 ),
            axis.text.x = element_blank(),
            axis.text.y = element_text( colour = 'black', size = 8 ),
            axis.title = element_text( colour = 'black', size = 9 ),
            legend.key.size = unit(0.9,"cm"),
            legend.position="none",
            axis.ticks.x = element_blank(),
            plot.title = element_text(size = 7)
      )

    plots[[k]] <- plot
    k<-k+1
  }
  multiplot(plotlist = plots, cols = 6,layout = matrix(1:12, nrow=2, byrow = TRUE))
}
dev.off()
```

Leave-one-out cross validation

```
group<-factor(c("Preg_AI","Preg_AI","Preg_AI","Preg_AI","Preg_AI","Preg_AI","Preg_NB","Preg_NB","Preg_NB","Preg_N
B","Preg_NB","Preg_NB","Not_Preg","Not_Preg","Not_Preg","Not_Preg","Not_Preg"), levels=c("Preg_AI","Preg_NB", "No
t_Preg"))
rm(out_list_IDs_LOOCV,count_pwbc_a,group_a)

cl <- makeCluster(12)
registerDoParallel(cl)

out_list_IDs_LOOCV<-foreach(i = c(1:12), .inorder=FALSE,.packages=c("edgeR","DESeq2"), .verbose=FALSE ) %dopar% {

group_a<-group[-i]
count_pwbc_a<-count_pwbc[,-i]

design<-model.matrix(~group_a)
dds<-DGEList(count=count_pwbc_a, group=group_a)
dds<-estimateDisp(dds, design, robust=TRUE)
dds<-glmFit(dds, design)
dds<-glmLRT(dds,coef="group_aPreg_NB")
res_preg_AI_preg_NB_edgeR<-topTags(dds,n=Inf)$table

#DESeq
colData<-data.frame("group"=group_a)
rownames(colData)<-colnames(count_pwbc_a)
dds<-DESeqDataSetFromMatrix(countData=count_pwbc_a, colData=colData, design= ~group)
dds<-DESeq(dds)
res_preg_AI_preg_NB_DeSeq<-results(dds, contrast=c("group","Preg_NB" ,"Preg_AI"), pAdjustMethod="fdr", tidy=TRUE)


merged_res_preg_AI_preg_NB_mRNA_a<-merge(res_preg_AI_preg_NB_edgeR, res_preg_AI_preg_NB_DeSeq, by.x="row.names",
 by.y="row")
merged_res_preg_AI_preg_NB_mRNA_a<-merged_res_preg_AI_preg_NB_mRNA_a[merged_res_preg_AI_preg_NB_mRNA_a$PValue<=0.
03 & merged_res_preg_AI_preg_NB_mRNA_a$pvalue<=0.03,]

rm(group_a,count_pwbc_a)

merged_res_preg_AI_preg_NB_mRNA_a$Row.names
}
stopCluster(cl)

overlapping_genes_LOOCV<-Reduce(intersect, out_list_IDs_LOOCV)

#merged_res_preg_AI_preg_NB_mRNA<-merge(res_preg_AI_preg_NB_edgeR, res_preg_AI_preg_NB_DeSeq, by.x="row.names", b
y.y="row")
merged_res_preg_AI_preg_NB_mRNA_a<-merged_res_preg_AI_preg_NB_mRNA[merged_res_preg_AI_preg_NB_mRNA$Row.names %in%
overlapping_genes_LOOCV,]
dim(merged_res_preg_AI_preg_NB_mRNA_a) #2
```

```
## [1]  2 12
```

```
merged_res_preg_AI_preg_NB_mRNA_a<-merged_res_preg_AI_preg_NB_mRNA_a[with(merged_res_preg_AI_preg_NB_mRNA_a, orde
r(logFC)), ]
DEG_AI_NB_mRNA<-merge(merged_res_preg_AI_preg_NB_mRNA_a,annotation.ensembl.symbol, by.x="Row.names", by.y="ensemb
l_gene_id", all=FALSE)
DEG_AI_NB_mRNA<-DEG_AI_NB_mRNA[with(DEG_AI_NB_mRNA, order(logFC)), ]
dim(DEG_AI_NB_mRNA)
```

```
## [1]  2 17
```

```
#head(DEG_AI_NB_mRNA)
#write.table(DEG_AI_NB_mRNA, paste(result_path, "2019_12_27_merged_AI_NB_mRNA_gene_names_after_LOOCV.txt", sep
="/"), quote=FALSE, sep="\t", row.names=FALSE)
rm(out_list_IDs_LOOCV)
```

```
knitr::kable(head(DEG_AI_NB_mRNA), caption = "First five rows AI pregnant vs NB pregnant",format = 'pandoc')
```

First five rows AI pregnant vs NB pregnant

	Row.names	logFC	logCPM	LR	PValue	FDR	baseMean	log2FoldChange	lfcSE	stat	pvalue	
2	ENSBTAG00000049416	2.888986	0.5520443	7.151642	0.0074895	0.9994483	31.42871	3.232006	1.130232	2.859596	0.0042418	0.999

	Row.names	logFC	logCPM	LR	PValue	FDR	baseMean	log2FoldChange	lfcSE	stat	pvalue	
1	ENSBTAG00000040188	6.887927	0.8187581	12.657093	0.0003741	0.9994483	37.68545	7.300996	1.748430	4.175745	0.0000297	0.310

Plot the charts for all genes inferred as differentially expressed between AI and NB pregnant

```
cpm_AIpreg<-cpm_pwbc[,c(1,2,3,4,5,6)]
cpm_NOTpreg<-cpm_pwbc[,c(13,14,15,16,17)]
cpm_NBpreg<-cpm_pwbc[,c(7,8,9,10,11,12)]

data_chart_1<-data.frame( stringsAsFactors=FALSE)
data_chart_2<-data.frame( stringsAsFactors=FALSE)

for (i in  seq(dim(DEG_AI_NB_mRNA)[1])){

  gene_id<-DEG_AI_NB_mRNA[i,1]
  gene_symbol<-DEG_AI_NB_mRNA$external_gene_name[i]
  if(gene_symbol==""){gene_symbol<-gene_id}

  cpm_AI<-cpm_AIpreg[rownames(cpm_AIpreg)==gene_id,]
  cpm_NB<-cpm_NBpreg[rownames(cpm_NBpreg)==gene_id,]

  data_chart_1<-rbind(data.frame(cpm=cpm_AI), data.frame(cpm=cpm_NB))
  data_chart_1$group<-rep(c("Preg_AI","Preg_NB"),c(6,6))
  data_chart_1$shape<-as.factor(c(0:11))
  data_chart_1$gene<-gene_symbol
  data_chart_1$chart<-i
  data_chart_1$fold_change<-DEG_AI_NB_mRNA[DEG_AI_NB_mRNA$Row.names==gene_id,]$logFC
  data_chart_1$pvalue<-DEG_AI_NB_mRNA[DEG_AI_NB_mRNA$Row.names==gene_id,]$PValue


  data_chart_2<-rbind(data_chart_2,data_chart_1)
}

pdf(file=paste(result_path,"2019_12_27_plot_all_DEGs_preg_AI_preg_NB_ordered_by_FC_after_LOOCV.pdf",sep="/"), wid
th= 3,height=2, bg="transparent", onefile=TRUE)

# for (i in seq(1,dim(DEG_AI_NB_mRNA)[1], 24)) {
  plots <- list()
  k<-1
  for (j in c(i:2)){
    data_chart_3<-data_chart_2[data_chart_2$chart %in% j , ]
    plot<-ggplot(data=data_chart_3, aes(x=group , y=cpm, colour=group))+
      geom_jitter(position = position_jitter(width = .1, height=0), aes( shape=shape), size=2)+
      scale_shape_manual(values=c(0:11))+
      scale_x_discrete(data_chart_3$gene[1],labels=c("Preg_AI"="Preg_AI", "Preg_NB"="Preg_NB"))+
      scale_colour_manual(name=NULL, values = c("blue", "black"))+
      stat_summary(fun.y = median, fun.ymin = median, fun.ymax = median,colour = "gray", size = 0.2,  geom = "cro
ssbar", alpha=0.5)+
      scale_y_continuous("CPM")+
      ggtitle(paste("LogFC","=", round(data_chart_3$fold_change[1],1),"  ","P","=",round(data_chart_3$pvalue[1],4
), sep=c("")))+
      theme_bw()+
      theme(panel.grid= element_blank(),
            panel.background = element_blank(),
            panel.grid.minor = element_blank(),
            panel.grid.major = element_blank(),
            plot.background = element_blank(),
            strip.background = element_rect(fill = "white"),
            #strip.text.x = element_text(colour = 'black', face="italic",size = 9),
            #legend.text = element_text( colour = 'black', size = 10 ),
            axis.text.x = element_blank(),
            axis.text.y = element_text( colour = 'black', size = 8 ),
            axis.title = element_text( colour = 'black', size = 9 ),
            legend.key.size = unit(0.9,"cm"),
            legend.position="none",
            axis.ticks.x = element_blank(),
            plot.title = element_text(size = 7)
      )

    plots[[k]] <- plot
    k<-k+1
  }
  multiplot(plotlist = plots, cols = 2,layout = matrix(1:2, nrow=1, byrow = TRUE))
#}
dev.off()
```

DEG Analysis for mRNA

Pregnant to Natural Service vs non pregnant

Results from edgeR

```
#colnames(count_pwbc)
group<-factor(c("Preg_AI","Preg_AI","Preg_AI","Preg_AI","Preg_AI","Preg_AI","Preg_NB","Preg_NB","Preg_NB","Preg_N
B","Preg_NB","Preg_NB","Not_Preg","Not_Preg","Not_Preg","Not_Preg","Not_Preg"), levels=c("Preg_NB","Preg_AI", "No
t_Preg"))
design<-model.matrix(~group)
dds<-DGEList(count=count_pwbc, group=group)
dds<-estimateDisp(dds, design, robust=TRUE)
dds<-glmFit(dds, design)
dds<-glmLRT(dds,coef="groupNot_Preg")
res_not_preg_preg_NB_edgeR<-topTags(dds,n=Inf)$table
#head(res_not_preg_preg_NB_edgeR,n=30)
#write.table(res_not_preg_preg_NB_edgeR, paste(result_path, "2019_04_30_res_not_preg_preg_NB_edgeR.txt"), quote=F
ALSE, sep="\t", row.names=FALSE)
```

Results from DESeq

```
design<-model.matrix(~group)
colData<-data.frame("group"=group)
rownames(colData)<-colnames(count_pwbc)
dds<-DESeqDataSetFromMatrix(countData=count_pwbc, colData=colData, design= ~group)
dds<-DESeq(dds)
res_not_preg_preg_NB_DeSeq<-results(dds, contrast=c("group","Not_Preg" ,"Preg_NB"), pAdjustMethod="fdr", tidy=TRU
E)
res_not_preg_preg_NB_DeSeq<-res_not_preg_preg_NB_DeSeq[with(res_not_preg_preg_NB_DeSeq, order(pvalue)), ]
#head(res_not_preg_preg_NB_DeSeq,n=30)
#write.table(res_not_preg_preg_NB_DeSeq, paste(result_path, "2019_04_30_res_not_preg_preg_NB_DeSeq.txt"), quote=F
ALSE, sep="\t", row.names=FALSE)
```

Merge the results for edgeR and DESeq. (Pregnant to Natural Service vs non pregnant)

```
merged_res_not_preg_preg_NB_mRNA<-merge(res_not_preg_preg_NB_edgeR, res_not_preg_preg_NB_DeSeq, by.x="row.names",
by.y="row")
merged_res_not_preg_preg_NB_mRNA<-merged_res_not_preg_preg_NB_mRNA[merged_res_not_preg_preg_NB_mRNA$PValue<=0.03
 & merged_res_not_preg_preg_NB_mRNA$pvalue<=0.03,]
dim(merged_res_not_preg_preg_NB_mRNA) #414
```

```
## [1] 414  12
```

```
#head(merged_res_not_preg_preg_NB_mRNA)
DEG_NB_NP_mRNA<-merge(merged_res_not_preg_preg_NB_mRNA,annotation.ensembl.symbol, by.x="Row.names", by.y="ensembl
_gene_id", all=FALSE)
DEG_NB_NP_mRNA<-DEG_NB_NP_mRNA[with(DEG_NB_NP_mRNA, order(logFC)), ]
#write.table(merged_res_not_preg_preg_NB_mRNA, paste(result_path, "2019_04_30_merged_res_not_preg_preg_NB_mRNA_be
fore_LOOCV.txt"), quote=FALSE, sep="\t", row.names=FALSE)
#write.table(DEG_NB_NP_mRNA, paste(result_path, "2019_12_27_merged_NB_NP_mRNA_gene_names_before_LOOCV.txt", sep
="/"), quote=FALSE, sep="\t", row.names=FALSE)
```

Plot the charts for all genes inferred as differentially expressed between NB pregnant and non pregnant

```
cpm_AIpreg<-cpm_pwbc[,c(1,2,3,4,5,6)]
cpm_NOTpreg<-cpm_pwbc[,c(13,14,15,16,17)]
cpm_NBpreg<-cpm_pwbc[,c(7,8,9,10,11,12)]

data_chart_1<-data.frame( stringsAsFactors=FALSE)
data_chart_2<-data.frame( stringsAsFactors=FALSE)

for (i in  seq(dim(DEG_NB_NP_mRNA)[1])){

  gene_id<-DEG_NB_NP_mRNA[i,1]
  gene_symbol<-DEG_NB_NP_mRNA$external_gene_name[i]
  if(gene_symbol==""){gene_symbol<-gene_id}

  cpm_NB<-cpm_NBpreg[rownames(cpm_NBpreg)==gene_id,]
  cpm_NP<-cpm_NOTpreg[rownames(cpm_NOTpreg)==gene_id,]

  data_chart_1<-rbind(data.frame(cpm=cpm_NB), data.frame(cpm=cpm_NP))
  data_chart_1$group<-rep(c("Preg_NB","Not_Preg"),c(6,5))
  data_chart_1$shape<-as.factor(c(0:10))
  data_chart_1$gene<-gene_symbol
  data_chart_1$chart<-i
  data_chart_1$fold_change<-DEG_NB_NP_mRNA[DEG_NB_NP_mRNA$Row.names==gene_id,]$logFC
  data_chart_1$pvalue<-DEG_NB_NP_mRNA[DEG_NB_NP_mRNA$Row.names==gene_id,]$PValue


  data_chart_2<-rbind(data_chart_2,data_chart_1)
}

pdf(file=paste(result_path,"2019_12_27_plot_all_DEGs_preg_NB_not_preg_ordered_by_FC_before_LOOCV.pdf",sep="/"), w
idth= 11, paper="USr", bg="transparent", onefile=TRUE)

for (i in seq(1,dim(DEG_NB_NP_mRNA)[1], 24)) {
  plots <- list()
  k<-1
  for (j in c(i:(i+23))){
    data_chart_3<-data_chart_2[data_chart_2$chart %in% j , ]
    plot<-ggplot(data=data_chart_3, aes(x=group , y=cpm, colour=group))+
      geom_jitter(position = position_jitter(width = .1, height=0), aes( shape=shape), size=2)+
      scale_shape_manual(values=c(0:10))+
      scale_x_discrete(data_chart_3$gene[1],labels=c("Preg_NB"="Preg_NB", "Not_Preg"="Not_Preg"))+
      scale_colour_manual(name=NULL, values = c("#D55E00", "#000000"))+
      stat_summary(fun.y = median, fun.ymin = median, fun.ymax = median,colour = "gray", size = 0.2,  geom = "cro
ssbar", alpha=0.5)+
      scale_y_continuous("CPM")+
      ggtitle(paste("LogFC","=", round(data_chart_3$fold_change[1],1),"  ","P","=",round(data_chart_3$pvalue[1],4
), sep=c("")))+
      theme_bw()+
      theme(panel.grid= element_blank(),
            panel.background = element_blank(),
            panel.grid.minor = element_blank(),
            panel.grid.major = element_blank(),
            plot.background = element_blank(),
            strip.background = element_rect(fill = "white"),
            #strip.text.x = element_text(colour = 'black', face="italic",size = 9),
            #legend.text = element_text( colour = 'black', size = 10 ),
            axis.text.x = element_blank(),
            axis.text.y = element_text( colour = 'black', size = 8 ),
            axis.title = element_text( colour = 'black', size = 9 ),
            legend.key.size = unit(0.9,"cm"),
            legend.position="none",
            axis.ticks.x = element_blank(),
            plot.title = element_text(size = 7)
      )

    plots[[k]] <- plot
    k<-k+1
  }
  multiplot(plotlist = plots, cols = 6,layout = matrix(1:24, nrow=4, byrow = TRUE))
}
dev.off()
```

Supplementary table 12

Leave-one-out cross validation

```
group<-factor(c("Preg_AI","Preg_AI","Preg_AI","Preg_AI","Preg_AI","Preg_AI","Preg_NB","Preg_NB","Preg_NB","Preg_N
B","Preg_NB","Preg_NB","Not_Preg","Not_Preg","Not_Preg","Not_Preg","Not_Preg"), levels=c("Preg_NB", "Preg_AI","No
t_Preg"))
rm(out_list_IDs_LOOCV,count_pwbc_a,group_a)

cl <- makeCluster(12)
registerDoParallel(cl)

out_list_IDs_LOOCV<-foreach(i = c(7:17), .inorder=FALSE,.packages=c("edgeR","DESeq2"), .verbose=FALSE ) %dopar% {

group_a<-group[-i]
count_pwbc_a<-count_pwbc[,-i]

design<-model.matrix(~group_a)
dds<-DGEList(count=count_pwbc_a, group=group_a)
dds<-estimateDisp(dds, design, robust=TRUE)
dds<-glmFit(dds, design)
dds<-glmLRT(dds,coef="group_aNot_Preg")
res_not_preg_preg_NB_edgeR<-topTags(dds,n=Inf)$table

#DESeq
colData<-data.frame("group"=group_a)
rownames(colData)<-colnames(count_pwbc_a)
dds<-DESeqDataSetFromMatrix(countData=count_pwbc_a, colData=colData, design= ~group)
dds<-DESeq(dds)
res_not_preg_preg_NB_DeSeq<-results(dds, contrast=c("group","Preg_NB" ,"Not_Preg"), pAdjustMethod="fdr", tidy=TRU
E)


merged_res_not_preg_preg_NB_mRNA_a<-merge(res_not_preg_preg_NB_edgeR, res_not_preg_preg_NB_DeSeq, by.x="row.name
s", by.y="row")
merged_res_not_preg_preg_NB_mRNA_a<-merged_res_not_preg_preg_NB_mRNA_a[merged_res_not_preg_preg_NB_mRNA_a$PValue<
=0.03 & merged_res_not_preg_preg_NB_mRNA_a$pvalue<=0.03,]

rm(group_a,count_pwbc_a)

merged_res_not_preg_preg_NB_mRNA_a$Row.names
}
stopCluster(cl)
overlapping_genes_LOOCV<-Reduce(intersect, out_list_IDs_LOOCV)

merged_res_not_preg_preg_NB_mRNA<-merge(res_not_preg_preg_NB_edgeR, res_not_preg_preg_NB_DeSeq, by.x="row.names",
by.y="row")
merged_res_not_preg_preg_NB_mRNA_a<-merged_res_not_preg_preg_NB_mRNA[merged_res_not_preg_preg_NB_mRNA$Row.names %
in% overlapping_genes_LOOCV,]
dim(merged_res_not_preg_preg_NB_mRNA_a) #81
```

```
## [1] 81 12
```

```
DEG_NB_NP_mRNA<-merge(merged_res_not_preg_preg_NB_mRNA_a,annotation.ensembl.symbol, by.x="Row.names", by.y="ensem
bl_gene_id", all=FALSE)
DEG_NB_NP_mRNA<-DEG_NB_NP_mRNA[with(DEG_NB_NP_mRNA, order(logFC)), ]
#write.table(DEG_NB_NP_mRNA, paste(result_path, "2019_12_27_merged_NB_NP_mRNA_gene_names_after_LOOCV.txt", sep
="/"), quote=FALSE, sep="\t", row.names=FALSE)
rm(out_list_IDs_LOOCV)
```

```
knitr::kable(head(DEG_NB_NP_mRNA), caption = "First five rows Supplementary table 12",format = 'pandoc')
```

First five rows Supplementary table 12

	Row.names	logFC	logCPM	LR	PValue	FDR	baseMean	log2FoldChange	lfcSE	stat	pvalue	
79	ENSBTAG00000052118	-4.492594	0.1925364	8.266168	0.0040391	0.5315921	17.10857	-4.674455	1.5654939	-2.985930	0.0028272	0.
3	ENSBTAG00000000517	-4.008047	1.6725720	13.410263	0.0002503	0.3283297	56.86040	-3.734326	0.9896552	-3.773360	0.0001611	0.
59	ENSBTAG00000034281	-3.406753	1.0671814	8.971424	0.0027423	0.5315921	36.88080	-3.330845	1.0879922	-3.061460	0.0022026	0.
41	ENSBTAG00000014791	-2.644484	1.6574465	7.079575	0.0077967	0.5464846	59.61641	-2.916556	0.9095127	-3.206724	0.0013426	0.

	Row.names	logFC	logCPM	LR	PValue	FDR	baseMean	log2FoldChange	lfcSE	stat	pvalue	
23	ENSBTAG00000008227	-2.366188	2.2527690	11.986830	0.0005358	0.3763416	88.74001	-2.406923	0.6201177	-3.881398	0.0001039	0.
75	ENSBTAG00000049285	-2.111184	3.1991884	9.014732	0.0026781	0.5315921	177.45930	-2.320851	0.6485780	-3.578368	0.0003457	0.

Plot the charts for all genes inferred as differentially expressed between NB pregnant and non pregnant

```
cpm_AIpreg<-cpm_pwbc[,c(1,2,3,4,5,6)]
cpm_NOTpreg<-cpm_pwbc[,c(13,14,15,16,17)]
cpm_NBpreg<-cpm_pwbc[,c(7,8,9,10,11,12)]

data_chart_1<-data.frame( stringsAsFactors=FALSE)
data_chart_2<-data.frame( stringsAsFactors=FALSE)

for (i in  seq(dim(DEG_NB_NP_mRNA)[1])){

  gene_id<-DEG_NB_NP_mRNA[i,1]
  gene_symbol<-DEG_NB_NP_mRNA$external_gene_name[i]
  if(gene_symbol==""){gene_symbol<-gene_id}

  cpm_NB<-cpm_NBpreg[rownames(cpm_NBpreg)==gene_id,]
  cpm_NP<-cpm_NOTpreg[rownames(cpm_NOTpreg)==gene_id,]

  data_chart_1<-rbind(data.frame(cpm=cpm_NB), data.frame(cpm=cpm_NP))
  data_chart_1$group<-rep(c("Preg_NB","Not_Preg"),c(6,5))
  data_chart_1$shape<-as.factor(c(0:10))
  data_chart_1$gene<-gene_symbol
  data_chart_1$chart<-i
  data_chart_1$fold_change<-DEG_NB_NP_mRNA[DEG_NB_NP_mRNA$Row.names==gene_id,]$logFC
  data_chart_1$pvalue<-DEG_NB_NP_mRNA[DEG_NB_NP_mRNA$Row.names==gene_id,]$PValue


  data_chart_2<-rbind(data_chart_2,data_chart_1)
}

pdf(file=paste(result_path,"2019_12_27_plot_all_DEGs_preg_NB_not_preg_ordered_by_FC_after_LOOCV.pdf",sep="/"), wi
dth= 11, paper="USr", bg="transparent", onefile=TRUE)

for (i in seq(1,dim(DEG_NB_NP_mRNA)[1], 24)) {
  plots <- list()
  k<-1
  for (j in c(i:(i+23))){
    data_chart_3<-data_chart_2[data_chart_2$chart %in% j , ]
    plot<-ggplot(data=data_chart_3, aes(x=group , y=cpm, colour=group))+
      geom_jitter(position = position_jitter(width = .1, height=0), aes( shape=shape), size=2)+
      scale_shape_manual(values=c(0:10))+
      scale_x_discrete(data_chart_3$gene[1],labels=c("Preg_NB"="Preg_NB", "Not_Preg"="Not_Preg"))+
      scale_colour_manual(name=NULL, values = c("#D55E00", "#000000"))+
      stat_summary(fun.y = median, fun.ymin = median, fun.ymax = median,colour = "gray", size = 0.2,  geom = "cro
ssbar", alpha=0.5)+
      scale_y_continuous("CPM")+
      ggtitle(paste("LogFC","=", round(data_chart_3$fold_change[1],1),"  ","P","=",round(data_chart_3$pvalue[1],4
), sep=c("")))+
      theme_bw()+
      theme(panel.grid= element_blank(),
            panel.background = element_blank(),
            panel.grid.minor = element_blank(),
            panel.grid.major = element_blank(),
            plot.background = element_blank(),
            strip.background = element_rect(fill = "white"),
            #strip.text.x = element_text(colour = 'black', face="italic",size = 9),
            #legend.text = element_text( colour = 'black', size = 10 ),
            axis.text.x = element_blank(),
            axis.text.y = element_text( colour = 'black', size = 8 ),
            axis.title = element_text( colour = 'black', size = 9 ),
            legend.key.size = unit(0.9,"cm"),
            legend.position="none",
            axis.ticks.x = element_blank(),
            plot.title = element_text(size = 7)
      )

    plots[[k]] <- plot
    k<-k+1
  }
  multiplot(plotlist = plots, cols = 6,layout = matrix(1:24, nrow=4, byrow = TRUE))
}
dev.off()
```

Test biological processes for enrichment with DEGs and calculate fold change enrichment. (Pregnant to Natural Service vs non pregnant)

```
test.genes<-data.frame(a=unique(c(as.character(merged_res_not_preg_preg_NB_mRNA_a$Row.names))), stringsAsFactors=
FALSE)
#(test.genes$a %in% all_genes$gene)
all_genes_numeric<-as.integer(all_genes$gene %in%test.genes$a)
names(all_genes_numeric)<-all_genes$gene
N_DEGs<-length(test.genes$a)

set.seed(9830)
pwf<-nullp(all_genes_numeric, bias.data=annotation.genelength.biomart_vector, plot.fit=FALSE )
GO_BP_Cats_mRNA_NB_NP<-goseq(pwf,gene2cat=annotation.GO.BP.biomart, method ="Sampling", repcnt = 7000, use_genes_
without_cat=FALSE)
GO_BP_Cats_mRNA_NB_NP<-GO_BP_Cats_mRNA_NB_NP[GO_BP_Cats_mRNA_NB_NP$numDEInCat>4,]
GO_BP_Cats_mRNA_NB_NP$BY_FDR<-p.adjust(GO_BP_Cats_mRNA_NB_NP$over_represented_pvalue, method ="fdr")
GO_BP_Cats_mRNA_NB_NP<-GO_BP_Cats_mRNA_NB_NP[with(GO_BP_Cats_mRNA_NB_NP, order(BY_FDR,over_represented_pvalue, -n
umDEInCat)), ]
#head(GO_BP_Cats_mRNA_NB_NP, n=20)

GO_BP_Cats_mRNA_NB_NP$fold_enrichment<-(GO_BP_Cats_mRNA_NB_NP$numDEInCat/N_DEGs)/(GO_BP_Cats_mRNA_NB_NP$numInCat/
N_expressed_genes)
annotation.GO.BP.biomart_testgenes<-annotation.GO.BP.biomart[annotation.GO.BP.biomart$ensembl_gene_id %in% test.g
enes$a, ]
GO_BP_Cats_mRNA_NB_NP<-merge(GO_BP_Cats_mRNA_NB_NP,annotation.GO.BP.biomart_testgenes, by.x="category", by.y="go_
id", all.x=TRUE, all.y=FALSE)
GO_BP_Cats_mRNA_NB_NP<-merge(GO_BP_Cats_mRNA_NB_NP, annotation.ensembl.symbol, by.x="ensembl_gene_id", by.y="ense
mbl_gene_id", all=FALSE, all.x=TRUE, all.y=FALSE)
GO_BP_Cats_mRNA_NB_NP<-GO_BP_Cats_mRNA_NB_NP[with(GO_BP_Cats_mRNA_NB_NP, order(BY_FDR,term)), ]
GO_BP_Cats_mRNA_NB_NP<-GO_BP_Cats_mRNA_NB_NP[GO_BP_Cats_mRNA_NB_NP$BY_FDR <0.1, ]
#write.table(GO_BP_Cats_mRNA_NB_NP, paste(result_path, "2019_05_07_GO_BP_Cats_mRNA_NB_NP.txt", sep="/"), quote=FA
LSE, sep="\t", row.names=FALSE)
```

```
knitr::kable(head(GO_BP_Cats_mRNA_NB_NP), caption = "First five rows",format = 'pandoc')
```

First five rows

	ensembl_gene_id	category	over_represented_pvalue	under_represented_pvalue	numDEInCat	numInCat	term	ontology	BY
2	ENSBTAG00000004211	GO:0007165 (GO:0007165)	0.0309956	0.9901443	6	348	signal transduction	BP	0.06
3	ENSBTAG00000010047	GO:0007165 (GO:0007165)	0.0309956	0.9901443	6	348	signal transduction	BP	0.06
4	ENSBTAG00000010447	GO:0007165 (GO:0007165)	0.0309956	0.9901443	6	348	signal transduction	BP	0.06
5	ENSBTAG00000015938	GO:0007165 (GO:0007165)	0.0309956	0.9901443	6	348	signal transduction	BP	0.06
10	ENSBTAG00000044071	GO:0007165 (GO:0007165)	0.0309956	0.9901443	6	348	signal transduction	BP	0.06
11	ENSBTAG00000049285	GO:0007165 (GO:0007165)	0.0309956	0.9901443	6	348	signal transduction	BP	0.06

Test molecular functions for enrichment with DEGs and calculate fold change enrichment. (Pregnant to Natural Service vs non pregnant)

```
set.seed(9830)
pwf<-nullp(all_genes_numeric, bias.data=annotation.genelength.biomart_vector, plot.fit=FALSE )
GO_MF_Cats_mRNA_NB_NP<-goseq(pwf,gene2cat=annotation.GO.MF.biomart, method ="Sampling", repcnt = 7000, use_genes_
without_cat=FALSE)
GO_MF_Cats_mRNA_NB_NP<-GO_MF_Cats_mRNA_NB_NP[GO_MF_Cats_mRNA_NB_NP$numDEInCat>4,]
GO_MF_Cats_mRNA_NB_NP$BY_FDR<-p.adjust(GO_MF_Cats_mRNA_NB_NP$over_represented_pvalue, method ="fdr")
GO_MF_Cats_mRNA_NB_NP<-GO_MF_Cats_mRNA_NB_NP[with(GO_MF_Cats_mRNA_NB_NP, order(BY_FDR,over_represented_pvalue, -n
umDEInCat)), ]
#head(GO_MF_Cats_mRNA_NB_NP, n=20)

GO_MF_Cats_mRNA_NB_NP$fold_enrichment<-(GO_MF_Cats_mRNA_NB_NP$numDEInCat/N_DEGs)/(GO_MF_Cats_mRNA_NB_NP$numInCat/
N_expressed_genes)
annotation.GO.MF.biomart_testgenes<-annotation.GO.MF.biomart[annotation.GO.MF.biomart$ensembl_gene_id %in% test.g
enes$a, ]
GO_MF_Cats_mRNA_NB_NP<-merge(GO_MF_Cats_mRNA_NB_NP,annotation.GO.MF.biomart_testgenes, by.x="category", by.y="go_
id", all.x=TRUE, all.y=FALSE)
GO_MF_Cats_mRNA_NB_NP<-merge(GO_MF_Cats_mRNA_NB_NP, annotation.ensembl.symbol, by.x="ensembl_gene_id", by.y="ense
mbl_gene_id", all=FALSE, all.x=TRUE, all.y=FALSE)
GO_MF_Cats_mRNA_NB_NP<-GO_MF_Cats_mRNA_NB_NP[with(GO_MF_Cats_mRNA_NB_NP, order(BY_FDR,term)), ]
GO_MF_Cats_mRNA_NB_NP<-GO_MF_Cats_mRNA_NB_NP[GO_MF_Cats_mRNA_NB_NP$BY_FDR<0.1, ]
#write.table(GO_MF_Cats_mRNA_NB_NP, paste(result_path, "2019_05_07_GO_MF_Cats_mRNA_NB_NP.txt"), quote=FALSE, sep
="\t", row.names=FALSE)
```

Pathway analysis through KEGG Pathways. (Pregnant to Natural Service vs non pregnant)

```
all_genes_entrez<- stack(mget(all_genes$gene, org.Bt.egENSEMBL2EG, ifnotfound = NA))
all_genes_entrez<-all_genes_entrez[complete.cases(all_genes_entrez),]
all_genes_entrez<-all_genes_entrez[!duplicated(all_genes_entrez$values),]

test.genes_entrez <- stack(mget(test.genes$a, org.Bt.egENSEMBL2EG, ifnotfound = NA))
test.genes_entrez<-test.genes_entrez[complete.cases(test.genes_entrez),]

all_genes_entrez_numeric<-as.integer(all_genes_entrez$values %in% test.genes_entrez$values)
names(all_genes_entrez_numeric)<-all_genes_entrez$values

entrez_kegg<- stack(mget(all_genes_entrez$values, org.Bt.egPATH, ifnotfound = NA))
entrez_kegg<-entrez_kegg[complete.cases(entrez_kegg),]
entrez_kegg<-entrez_kegg[,c(2,1)]
entrez_kegg_test_genes<-entrez_kegg[entrez_kegg$ind %in% test.genes_entrez$values,]

pwf<-nullp(all_genes_entrez_numeric, 'bosTau4','refGene', plot.fit=FALSE )

set.seed(8503)
kegg_NB_NP_subset<-goseq(pwf, gene2cat=entrez_kegg,method ="Sampling", repcnt = 7000, use_genes_without_cat=FALSE
)
kegg_NB_NP_subset<-kegg_NB_NP_subset[kegg_NB_NP_subset$numDEInCat>4,]
kegg_NB_NP_subset$BY_FDR<-p.adjust(kegg_NB_NP_subset$over_represented_pvalue, method ="fdr")
kegg_NB_NP_subset<-merge(kegg_NB_NP_subset,entrez_kegg_test_genes, by.x="category", by.y="values", all.x=TRUE, al
l.y=FALSE)
kegg_NB_NP_subset<-merge(kegg_NB_NP_subset,all_genes_entrez, by.x="ind", by.y="values", all.x=TRUE, all.y=FALSE,
 suffixes =c('.entrez','.ensembl'))
kegg_NB_NP_subset<-kegg_NB_NP_subset[with(kegg_NB_NP_subset, order(BY_FDR, -numDEInCat)), ]
kegg_NB_NP_subset<-kegg_NB_NP_subset[,c(2:8)]
kegg_NB_NP_subset<-merge(kegg_NB_NP_subset, annotation.ensembl.symbol, by.x="ind.ensembl", by.y="ensembl_gene_id"
, all.x=TRUE, all.y=FALSE)
kegg_NB_NP_subset<-kegg_NB_NP_subset[with(kegg_NB_NP_subset, order(BY_FDR, -numDEInCat)), ]
kegg_NB_NP_subset<-kegg_NB_NP_subset[kegg_NB_NP_subset$BY_FDR<0.1,]
#write.table(kegg_NB_NP_subset, paste(result_path, "2019_12_27_kegg_NB_NP_subset.txt", sep="/"), quote=FALSE, sep
="\t", row.names=FALSE)
```

```
knitr::kable(head(kegg_NB_NP_subset), caption = "First five rows KEEG pathways NB vs NP",format = 'pandoc')
```

First five rows KEEG pathways NB vs NP

	ind.ensembl	category	over_represented_pvalue	under_represented_pvalue	numDEInCat	numInCat	BY_FDR	external_gene_name
1	ENSBTAG00000000273	04060	0.0022854	0.9997143	5	100	0.0045708	IL5RA
4	ENSBTAG00000004211	04060	0.0022854	0.9997143	5	100	0.0045708	TNFRSF1A

	ind.ensembl	category	over_represented_pvalue	under_represented_pvalue	numDEInCat	numInCat	BY_FDR	external_gene_name
8	ENSBTAG00000012899	04060	0.0022854	0.9997143	5	100	0.0045708	IFNGR2
11	ENSBTAG00000020674	04060	0.0022854	0.9997143	5	100	0.0045708	LTB
14	ENSBTAG00000049285	04060	0.0022854	0.9997143	5	100	0.0045708	TNFRSF17

Figure 4a

```
DEG_AI_NB_only<-unique(DEG_AI_NB_mRNA$Row.names[! DEG_AI_NB_mRNA$Row.names %in% c(DEG_AI_NP_mRNA$Row.names, DEG_N
B_NP_mRNA$Row.names)]  )
DEG_AI_NB_AI_NP<-unique(DEG_AI_NB_mRNA$Row.names[DEG_AI_NB_mRNA$Row.names %in% c(DEG_AI_NP_mRNA$Row.names, DEG_NB
_NP_mRNA$Row.names)]  )
DEG_AI_NP_only<-unique(DEG_AI_NP_mRNA$Row.names[! (DEG_AI_NP_mRNA$Row.names %in% c(DEG_AI_NB_mRNA$Row.names, DEG_
NB_NP_mRNA$Row.names))]  )
DEG_AI_NP_NB_NP<-unique(DEG_NB_NP_mRNA$Row.names[ (DEG_NB_NP_mRNA$Row.names %in% DEG_AI_NP_mRNA$Row.names)]  )
DEG_NB_NP_only<-unique(DEG_NB_NP_mRNA$Row.names[! (DEG_NB_NP_mRNA$Row.names %in% c(DEG_AI_NB_mRNA$Row.names, DEG_
AI_NP_mRNA$Row.names))]  )

DEG_AI_NP_only<-DEG_AI_NP_only[flashClust(dist(log2(rpkm_pwbc[c(DEG_AI_NP_only),]+1)), method = "complete")$orde
r]
DEG_AI_NP_NB_NP<-DEG_AI_NP_NB_NP[flashClust(dist(log2(rpkm_pwbc[c(DEG_AI_NP_NB_NP),]+1)), method = "complete")$or
der]
DEG_NB_NP_only<-DEG_NB_NP_only[flashClust(dist(log2(rpkm_pwbc[c(DEG_NB_NP_only),]+1)), method = "complete")$orde
r]

rpkm_AIpreg_heatmap<-log2(rpkm_AIpreg[rownames(rpkm_AIpreg)%in%c(DEG_AI_NB_only,DEG_AI_NB_AI_NP, DEG_AI_NP_only,
 DEG_AI_NP_NB_NP, DEG_NB_NP_only),]+1)
rpkm_AIpreg_heatmap <- rpkm_AIpreg_heatmap[c(DEG_AI_NB_only,DEG_AI_NB_AI_NP, DEG_AI_NP_only, DEG_AI_NP_NB_NP, DEG
_NB_NP_only),]

rpkm_NBpreg_heatmap<-log2(rpkm_NBpreg[rownames(rpkm_NBpreg)%in%c(DEG_AI_NB_only,DEG_AI_NB_AI_NP, DEG_AI_NP_only,
 DEG_AI_NP_NB_NP, DEG_NB_NP_only),]+1)
rpkm_NBpreg_heatmap <- rpkm_NBpreg_heatmap[c(DEG_AI_NB_only,DEG_AI_NB_AI_NP, DEG_AI_NP_only, DEG_AI_NP_NB_NP, DEG
_NB_NP_only),]

rpkm_NOTpreg_heatmap<-log2(rpkm_NOTpreg[rownames(rpkm_NOTpreg)%in%c(DEG_AI_NB_only,DEG_AI_NB_AI_NP, DEG_AI_NP_onl
y, DEG_AI_NP_NB_NP, DEG_NB_NP_only),]+1)
rpkm_NOTpreg_heatmap <- rpkm_NOTpreg_heatmap[c(DEG_AI_NB_only,DEG_AI_NB_AI_NP, DEG_AI_NP_only, DEG_AI_NP_NB_NP, D
EG_NB_NP_only),]

sample_Tree_AI <- flashClust(as.dist((1-cor(rpkm_AIpreg_heatmap))/2), method = "complete")
rpkm_AIpreg_heatmap<-rpkm_AIpreg_heatmap[,sample_Tree_AI$order ]
sample_Tree_NB <- flashClust(as.dist((1-cor(rpkm_NBpreg_heatmap))/2), method = "complete")
rpkm_NBpreg_heatmap<-rpkm_NBpreg_heatmap[,sample_Tree_NB$order ]
sample_Tree_NP<- flashClust(as.dist((1-cor(rpkm_NOTpreg_heatmap))/2), method = "complete")
rpkm_NOTpreg_heatmap<-data.frame(rpkm_NOTpreg_heatmap[,sample_Tree_NP$order ])

rpkm_NOTpreg_heatmap$gene_id<-rownames(rpkm_NOTpreg_heatmap)
rpkm_NOTpreg_heatmap$gene_symbol<-annotation.ensembl.symbol$external_gene_name[match( rpkm_NOTpreg_heatmap$gene_i
d, annotation.ensembl.symbol$ensembl_gene_id )]
rpkm_NOTpreg_heatmap$gene_symbol<-ifelse(rpkm_NOTpreg_heatmap$gene_symbol=="", rpkm_NOTpreg_heatmap$gene_id, rpkm
_NOTpreg_heatmap$gene_symbol)

rownames(rpkm_NOTpreg_heatmap)<-rpkm_NOTpreg_heatmap$gene_symbol
rpkm_NOTpreg_heatmap<-rpkm_NOTpreg_heatmap[,c(1:5)]

annot_matrix<-cbind(rep(c(1,0,0,0,0),c(1,1,40,26,55)) , rep(c(0,1,0,0,0),c(1,1,40,26,55)), rep(c(0,0,1,0,0),c(1,1
,40,26,55)),rep(c(0,0,0,1,0),c(1,1,40,26,55)), rep(c(0,0,0,0,1),c(1,1,40,26,55)))

annotation<-Heatmap(annot_matrix,
        cluster_rows= FALSE,
        cluster_columns = FALSE,
        show_row_names = FALSE,
        show_column_names = FALSE,
        show_heatmap_legend = FALSE,
        col = colorRamp2(c(0,1), c("white","black"), space = "LAB"),
        split= rep(c(1,2,3,4,5), c(length(DEG_AI_NB_only),length(DEG_AI_NB_AI_NP),length(DEG_AI_NP_only),length(D
EG_AI_NP_NB_NP),length(DEG_NB_NP_only))),
        width = unit(5, "mm"))

ht_AIpreg<-Heatmap(rpkm_AIpreg_heatmap,
        column_title = "AI pregnant",
        column_title_gp = gpar(fill = "#0072B2", col = "white", border = "#FFFFFF", fontsize=9),
        cluster_rows= FALSE,
        cluster_columns = FALSE,
        show_row_names = FALSE,
        show_column_names = FALSE,
        show_heatmap_legend = FALSE,
        col = colorRamp2(c(0,15), c("#eef5fb","#3182bd"), space = "LAB"),
        split= rep(c(1,2,3,4,5), c(length(DEG_AI_NB_only),length(DEG_AI_NB_AI_NP),length(DEG_AI_NP_only),length(D
EG_AI_NP_NB_NP),length(DEG_NB_NP_only))),
        width = unit(2, "cm"))

ht_NBpreg<-Heatmap(rpkm_NBpreg_heatmap,
        column_title = "NB pregnant",
        column_title_gp = gpar(fill = "#000000", col = "white", border = "#FFFFFF", fontsize=9),
        cluster_rows= FALSE,
        cluster_columns = FALSE,
        show_row_names = FALSE,
```

```
        show_column_names = FALSE,
        show_heatmap_legend = FALSE,
        col = colorRamp2(c(0,15), c("#eef5fb","#3182bd"), space = "LAB"),
        split= rep(c(1,2,3,4,5), c(length(DEG_AI_NB_only),length(DEG_AI_NB_AI_NP),length(DEG_AI_NP_only),length(D
EG_AI_NP_NB_NP),length(DEG_NB_NP_only))),
        width = unit(2, "cm"))

ht_NOTpreg<-Heatmap(rpkm_NOTpreg_heatmap,
        column_title = "non pregnant",
        column_title_gp = gpar(fill = "#D55E00", col = "white", border = "#FFFFFF", fontsize=9),
        cluster_rows= FALSE,
        cluster_columns = FALSE,
        show_row_names = TRUE,
        row_names_gp = gpar(fontsize=4),
        show_column_names = FALSE,
        show_heatmap_legend = TRUE,
        heatmap_legend_param = list(title = "Log2(FPKM+1)", direction = "horizontal"),
        col = colorRamp2(c(0,15), c("#eef5fb","#3182bd"), space = "LAB"),
        split= rep(c(1,2,3,4,5), c(length(DEG_AI_NB_only),length(DEG_AI_NB_AI_NP),length(DEG_AI_NP_only),length(D
EG_AI_NP_NB_NP),length(DEG_NB_NP_only))),
        width = unit(2, "cm"))

#png(paste(result_path,"2020_01_03_DEG_heatmap.png", sep="/"),width = 1600, height = 2000,res = 300)
#draw(annotation + ht_AIpreg + ht_NBpreg + ht_NOTpreg)
#dev.off()

draw(annotation + ht_AIpreg + ht_NBpreg + ht_NOTpreg)
```

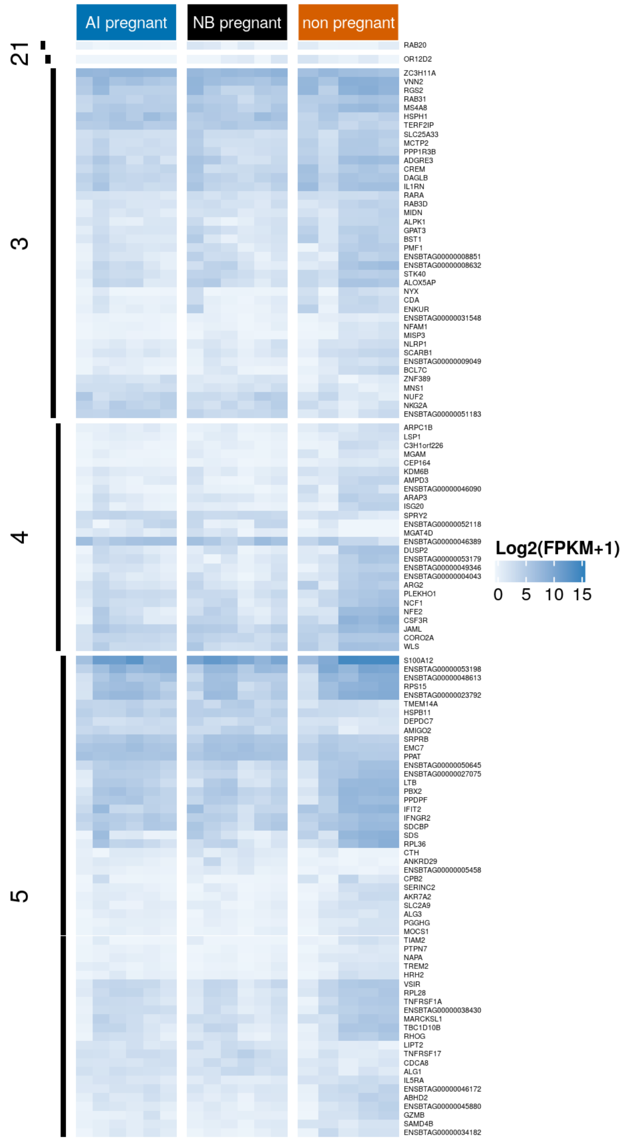

```
merged_correlation_mRNA_miRNA_DEG_AI_NB_only<- merged_correlation_mRNA_miRNA[merged_correlation_mRNA_miRNA$mRNA_g
ene %in% c(DEG_AI_NB_only),]
merged_correlation_mRNA_miRNA_DEG_AI_NB_AI_NP<- merged_correlation_mRNA_miRNA[merged_correlation_mRNA_miRNA$mRNA_
gene %in% c(DEG_AI_NB_AI_NP),]
merged_correlation_mRNA_miRNA_DEG_AI_NP_only<- merged_correlation_mRNA_miRNA[merged_correlation_mRNA_miRNA$mRNA_g
ene %in% c(DEG_AI_NP_only),]
merged_correlation_mRNA_miRNA_DEG_AI_NP_NB_NP<- merged_correlation_mRNA_miRNA[merged_correlation_mRNA_miRNA$mRNA_
gene %in% c( DEG_AI_NP_NB_NP),]
merged_correlation_mRNA_miRNA_DEG_NB_NP_only<- merged_correlation_mRNA_miRNA[merged_correlation_mRNA_miRNA$mRNA_g
ene %in% c( DEG_NB_NP_only),]


#head(DEG_AI_NP_mRNA)
#head(DEG_NB_NP_mRNA)

merged_DEG_AI_NP_mRNA_DEG_NB_NP_mRNA<-merge(DEG_AI_NP_mRNA, DEG_NB_NP_mRNA, by="Row.names", all=TRUE)

#head(merged_DEG_AI_NP_mRNA_DEG_NB_NP_mRNA)

merged_DEG_AI_NP_mRNA_DEG_NB_NP_mRNA_subset<-merged_DEG_AI_NP_mRNA_DEG_NB_NP_mRNA[ (merged_DEG_AI_NP_mRNA_DEG_NB_
NP_mRNA$logFC.x < 0 & merged_DEG_AI_NP_mRNA_DEG_NB_NP_mRNA$logFC.y < 0) |
(merged_DEG_AI_NP_mRNA_DEG_NB_NP_mRNA$logFC.x > 0 & merged_DEG_AI_NP_mRNA_DEG_NB_NP_mRNA$logFC.y > 0),]

merged_DEG_AI_NP_mRNA_DEG_NB_NP_mRNA_subset<-merged_DEG_AI_NP_mRNA_DEG_NB_NP_mRNA_subset[complete.cases(merged_DE
G_AI_NP_mRNA_DEG_NB_NP_mRNA_subset),]


merged_DEG_AI_NP_mRNA_DEG_NB_NP_mRNA_subset_miRNA<-merge(merged_DEG_AI_NP_mRNA_DEG_NB_NP_mRNA_subset,merged_corre
lation_mRNA_miRNA, by.x="Row.names", by.y="mRNA_gene", all=FALSE)

#merged_DEG_AI_NP_mRNA_DEG_NB_NP_mRNA_subset_miRNA
#write.table(merged_DEG_AI_NP_mRNA_DEG_NB_NP_mRNA_subset_miRNA, paste(result_path, "2020_01_04_merged_DEG_AI_NP_m
RNA_DEG_NB_NP_mRNA_subset_miRNA.txt", sep="/"), quote=FALSE, sep="\t", row.names=FALSE)
```

Figure 4b

```
cpm_AIpreg<-cpm_pwbc[,c(1,2,3,4,5,6)]
cpm_NOTpreg<-cpm_pwbc[,c(13,14,15,16,17)]
cpm_NBpreg<-cpm_pwbc[,c(7,8,9,10,11,12)]

data_chart_1<-data.frame( stringsAsFactors=FALSE)
data_chart_2<-data.frame( stringsAsFactors=FALSE)

for (i in  seq(dim(merged_DEG_AI_NP_mRNA_DEG_NB_NP_mRNA_subset)[1])){
  gene_id<-merged_DEG_AI_NP_mRNA_DEG_NB_NP_mRNA_subset[i,1]
  gene_symbol<-merged_DEG_AI_NP_mRNA_DEG_NB_NP_mRNA_subset$external_gene_name.x[i]
  if(gene_symbol==""){gene_symbol<-gene_id}

  cpm_AI<-cpm_AIpreg[rownames(cpm_AIpreg)==gene_id,]
  cpm_NB<-cpm_NBpreg[rownames(cpm_NBpreg)==gene_id,]
  cpm_NP<-cpm_NOTpreg[rownames(cpm_NOTpreg)==gene_id,]

  data_chart_1<-rbind(data.frame(cpm=cpm_AI),data.frame(cpm=cpm_NB), data.frame(cpm=cpm_NP))
  data_chart_1$group<-rep(c("Preg_AI","Preg_NB","Not_Preg"),c(6,6,5))
  data_chart_1$gene<-gene_symbol
  data_chart_2<-rbind(data_chart_2,data_chart_1)
}

data_chart_2$group<-factor(data_chart_2$group,levels =c( "Preg_AI" , "Preg_NB" , "Not_Preg"))
plot<-ggplot(data=data_chart_2, aes(x=group , y=cpm, colour=group))+
      geom_jitter(position = position_jitter(width = .1, height=0),  size=1)+
      scale_shape_manual(values=c(0:10))+
      scale_x_discrete(labels=c("Preg_AI"="Preg_AI" , "Preg_NB"="Preg_NB", "Not_Preg"="Not_Preg"))+
      scale_colour_manual(name=NULL, values = c("#0072B2",  "#000000", "#D55E00" ))+
      stat_summary(fun.y = median, fun.ymin = median, fun.ymax = median,colour = "gray", size = 0.2,  geom = "cro
ssbar", alpha=0.5)+
      scale_y_continuous("CPM")+
      facet_wrap(~gene, scales = "free", ncol=4)+
      theme_bw()+
      theme(panel.grid= element_blank(),
            panel.background = element_blank(),
            panel.grid.minor = element_blank(),
            panel.grid.major = element_blank(),
            plot.background = element_blank(),
            strip.background = element_rect(fill = "white"),
            strip.text.x = element_text(colour = 'black', size = 4),
            #legend.text = element_text( colour = 'black', size = 10 ),
            axis.text.x = element_blank(),
            axis.text.y = element_text( colour = 'black', size = 4 ),
            axis.title.y = element_text( colour = 'black', size = 4 ),
            axis.title.x = element_blank(),
            legend.text = element_text( colour = 'black', size = 4 ),
            legend.key.size = unit(0.4,"cm"),
            legend.position=c(0.85, 0.05),
            axis.ticks.x = element_blank(),
            panel.spacing.x=unit(0.5, "mm"),
            panel.spacing.y=unit(0.5, "mm")

      )
#png(file=paste(result_path,"2020_01_05_plot_DEGs_preg_AI_not_preg_NB_not_preg_after_LOOCV.png",sep="/"), width=
 1000, height=2000, bg="transparent",res=300 )
    #plot
#dev.off()
plot
```

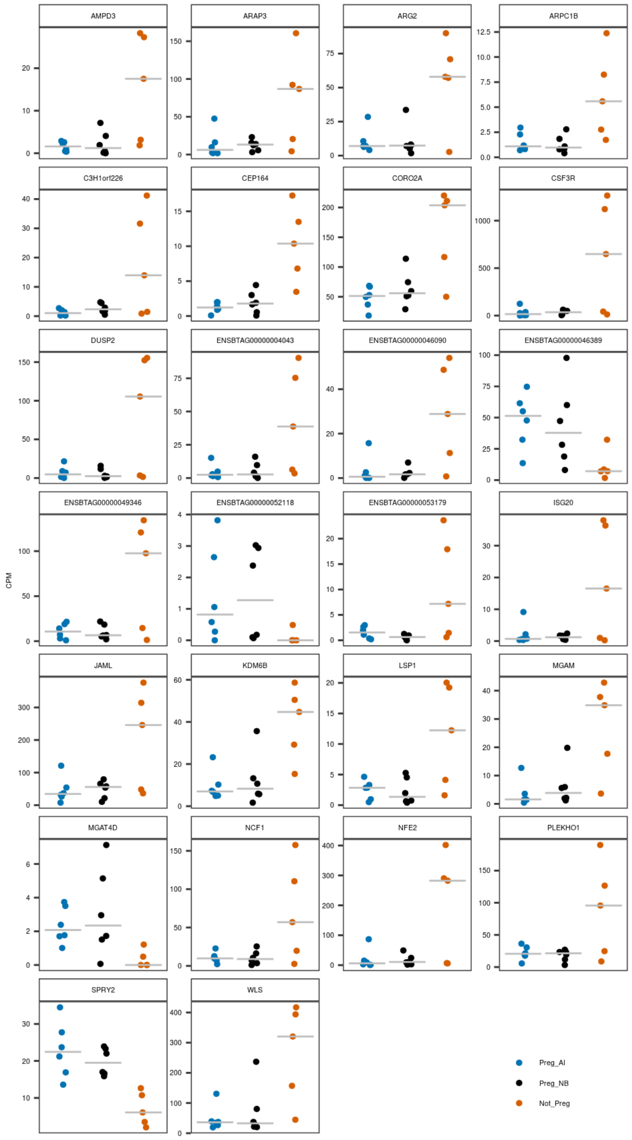
AMPD3
ARAP3
ARG2
ARPC1B
C3H1orf226
CEP164
CORO2A
CSF3R
DUSP2
ENSBTAG00000004043
ENSBTAG00000046090
ENSBTAG00000046389
ENSBTAG00000049346
ENSBTAG00000052118
ENSBTAG00000053179
ISG20
JAML
KDM6B
LSP1
MGAM
MGAT4D
NCF1
NFE2
PLEKHO1
SPRY2
WLS
CPM
Preg_AI
Preg_NB
Not_Preg

Figure 4c

```
merged_DEG_AI_NP_mRNA_DEG_NB_NP_mRNA_subset_miRNA_a<-merged_DEG_AI_NP_mRNA_DEG_NB_NP_mRNA_subset_miRNA[!(merged_D
EG_AI_NP_mRNA_DEG_NB_NP_mRNA_subset_miRNA$description.x==""),]
merged_DEG_AI_NP_mRNA_DEG_NB_NP_mRNA_subset_miRNA_a[10,41]<-"FCGR2"
merged_DEG_AI_NP_mRNA_DEG_NB_NP_mRNA_subset_miRNA_a[20,41]<-"CEACAM4"

data_for_chart<-data.frame()
data_for_chart_a<-data.frame()
for(i in 1:dim(merged_DEG_AI_NP_mRNA_DEG_NB_NP_mRNA_subset_miRNA_a)[1] ){
  expression_gene_1_AI<-cpm_AIpreg[row.names(cpm_AIpreg)==as.character(merged_DEG_AI_NP_mRNA_DEG_NB_NP_mRNA_subse
t_miRNA_a$Row.names)[i],]
  expression_gene_2_AI<-cpm_AIpreg_miRNA[row.names(cpm_AIpreg_miRNA)==as.character(merged_DEG_AI_NP_mRNA_DEG_NB_N
P_mRNA_subset_miRNA_a$miRNA_gene)[i],]
  expression_gene_1_NB<-cpm_NBpreg[row.names(cpm_NBpreg)==as.character(merged_DEG_AI_NP_mRNA_DEG_NB_NP_mRNA_subse
t_miRNA_a$Row.names)[i],]
  expression_gene_2_NB<-cpm_NBpreg_miRNA[row.names(cpm_NBpreg_miRNA)==as.character(merged_DEG_AI_NP_mRNA_DEG_NB_N
P_mRNA_subset_miRNA_a$miRNA_gene)[i],]
  expression_gene_1_NP<-cpm_NOTpreg[row.names(cpm_NOTpreg)==as.character(merged_DEG_AI_NP_mRNA_DEG_NB_NP_mRNA_sub
set_miRNA_a$Row.names)[i],]
  expression_gene_2_NP<-cpm_NOTpreg_miRNA[row.names(cpm_NOTpreg_miRNA)==as.character(merged_DEG_AI_NP_mRNA_DEG_NB
_NP_mRNA_subset_miRNA_a$miRNA_gene)[i],]

  symbol_gene_1<-as.character(merged_DEG_AI_NP_mRNA_DEG_NB_NP_mRNA_subset_miRNA_a$mRNA_symbol)[i]
  symbol_gene_2<-as.character(merged_DEG_AI_NP_mRNA_DEG_NB_NP_mRNA_subset_miRNA_a$miRNA_symbol)[i]

  chart<-i

  data_for_chart<-data.frame(gene_1=c(expression_gene_1_AI,expression_gene_1_NB,expression_gene_1_NP) ,
                             gene_2=c(expression_gene_2_AI,expression_gene_2_NB,expression_gene_2_NP),
                             symbol_gene_1,symbol_gene_2 ,chart,
                             group=rep(c("AI", "NB", "NP"), c(length(expression_gene_1_AI),length(expression_gene
_1_NB),length(expression_gene_1_NP))))

  data_for_chart_a<-rbind(data_for_chart_a,data_for_chart)
}

font_axis<-5
font_title<-5
ptsize<-0.05

plots<-list()
k<-1
for (j in c(1:20)){
  data_for_chart_b<-data_for_chart_a[data_for_chart_a$chart %in% j,]

plot<-ggplot(data_for_chart_b, aes(y=gene_1,x=gene_2))+
  geom_point(size=0.5)+
  geom_smooth(method=lm, size=ptsize, fill="lightgray")+
  scale_y_continuous(name=data_for_chart_b$symbol_gene_1[1])+
  scale_x_continuous(name=data_for_chart_b$symbol_gene_2[1])+
  facet_wrap(~group, ncol=3, scales="free")+
  theme(aspect.ratio = 1,
            panel.grid.major = element_blank(),
            panel.grid.minor = element_blank(),
            panel.background = element_blank(),
            plot.background  = element_blank(),
            axis.text.x = element_text( colour = 'black',size = font_axis, angle=90),
            axis.text.y = element_text( colour = 'black',size = font_axis),
            axis.title = element_text( colour = 'black' ,size = font_title, face="italic"),
            axis.ticks = element_line(size=0.1),
            panel.spacing.y = unit(0, "mm"),
            panel.spacing.x = unit(1.5, "mm"),
            legend.position="none",
            axis.line=element_line(size = 0.1, colour = "black"),
            strip.background=element_blank(),
            strip.text.x = element_blank())

 plots[[k]]<-plot
  k<-k+1
}

#png(filename =paste(result_path,"2020_02_19_plot_DE_diff_coexpression.png", sep="/"), width = 9, height =3, unit
s = 'in', res = 300,bg = "transparent")
#ggarrange(plotlist=plots, ncol=5, nrow=4)
#dev.off()
ggarrange(plotlist=plots, ncol=5, nrow=4)
```

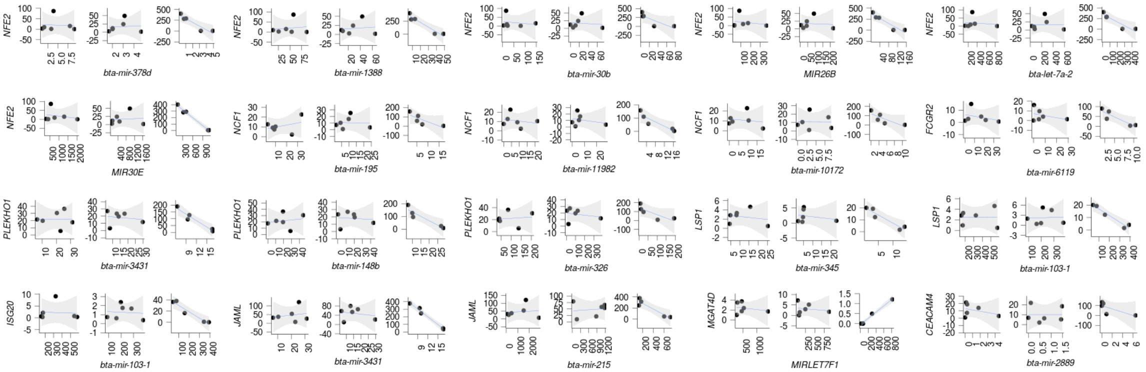

DEG Analysis for miRNA

Pregnant to Artificial Insemination vs non pregnant

Results from edgeR

```
colnames(count_miRNA_plasma)
```

```
##  [1] "AI_92"  "AI_99"  "AI_114" "AI_117" "AI_119" "AI_120" "NB_93"  "NB_103"
##  [9] "NB_108" "NB_111" "NB_116" "NB_121" "NP_94"  "NP_101" "NP_107" "NP_113"
## [17] "NP_115"
```

```
group<-factor(c("Preg_AI","Preg_AI","Preg_AI","Preg_AI","Preg_AI","Preg_AI","Preg_NB","Preg_NB","Preg_NB","Preg_N
B","Preg_NB","Preg_NB","Not_Preg","Not_Preg","Not_Preg","Not_Preg","Not_Preg"), levels=c("Preg_AI","Preg_NB", "No
t_Preg"))
design<-model.matrix(~group)
dds<-DGEList(count=count_miRNA_plasma, group=group)
dds<-estimateDisp(dds, design, robust=TRUE)
dds<-glmFit(dds, design)
dds<-glmLRT(dds,coef="groupNot_Preg")
res_not_preg_preg_AI_miRNA_edgeR<-topTags(dds,n=Inf)$table
#head(res_not_preg_preg_AI_miRNA_edgeR,n=30)
#write.table(res_not_preg_preg_AI_miRNA_edgeR, paste(result_path, "2019_04_30_res_not_preg_preg_AI_miRNA_edgeR.tx
t"), quote=FALSE, sep="\t", row.names=FALSE)
```

Results from DESeq

```
design<-model.matrix(~group)
colData<-data.frame("group"=group)
rownames(colData)<-colnames(count_miRNA_plasma)
dds<-DESeqDataSetFromMatrix(countData=count_miRNA_plasma, colData=colData, design= ~group)
dds<-DESeq(dds)
res_not_preg_preg_AI_miRNA_DeSeq<-results(dds, contrast=c("group","Not_Preg" ,"Preg_AI"), pAdjustMethod="fdr", ti
dy=TRUE)
res_not_preg_preg_AI_miRNA_DeSeq<-res_not_preg_preg_AI_miRNA_DeSeq[with(res_not_preg_preg_AI_miRNA_DeSeq, order(p
value)), ]
#head(res_not_preg_preg_AI_miRNA_DeSeq,n=30)
#write.table(res_not_preg_preg_AI_miRNA_DeSeq, paste(result_path, "2019_04_30_res_not_preg_preg_AI_miRNA_DeSeq.tx
t"), quote=FALSE, sep="\t", row.names=FALSE)
```

Merge and annotate the results from edgeR and DESeq. (AI Pregnant vs non pregnant)

```
merged_res_not_preg_preg_AI_miRNA<-merge(res_not_preg_preg_AI_miRNA_edgeR, res_not_preg_preg_AI_miRNA_DeSeq, by.x
="row.names", by.y="row")
merged_res_not_preg_preg_AI_miRNA<-merged_res_not_preg_preg_AI_miRNA[merged_res_not_preg_preg_AI_miRNA$PValue<=0.
03 & merged_res_not_preg_preg_AI_miRNA$pvalue<=0.03,]
dim(merged_res_not_preg_preg_AI_miRNA) #8
```

```
## [1]  8 12
```

```
merged_res_not_preg_preg_AI_miRNA<-merged_res_not_preg_preg_AI_miRNA[with(merged_res_not_preg_preg_AI_miRNA, orde
r(logFC)), ]
#head(merged_res_not_preg_preg_AI_miRNA)
#write.table(res_not_preg_preg_AI_miRNA_DeSeq, paste(result_path, "2019_04_30_res_not_preg_preg_AI_miRNA_DeSeq.tx
t"), quote=FALSE, sep="\t", row.names=FALSE)

annotated_merged_res_not_preg_preg_AI_miRNA<-merge(merged_res_not_preg_preg_AI_miRNA,annotation.ensembl.symbol, b
y.x="Row.names", by.y="ensembl_gene_id", all=FALSE)
#head(annotated_merged_res_not_preg_preg_AI_miRNA)
annotated_merged_res_not_preg_preg_AI_miRNA<-annotated_merged_res_not_preg_preg_AI_miRNA[with(annotated_merged_re
s_not_preg_preg_AI_miRNA, order(logFC)), ]
dim(annotated_merged_res_not_preg_preg_AI_miRNA) #7
```

```
## [1]  7 17
```

```
#write.table(annotated_merged_res_not_preg_preg_AI_miRNA, paste(result_path, "2019_04_30_annotated_merged_res_not
_preg_preg_AI_miRNA.txt"), quote=FALSE, sep="\t", row.names=FALSE)
```

Plot the charts for all genes inferred as differentially expressed between AI and non pregnant

```
cpm_AIpreg<-cpm_miRNA[,c(1,2,3,4,5,6)]
cpm_NOTpreg<-cpm_miRNA[,c(13,14,15,16,17)]
cpm_NBpreg<-cpm_miRNA[,c(7,8,9,10,11,12)]

data_chart_1<-data.frame( stringsAsFactors=FALSE)
data_chart_2<-data.frame( stringsAsFactors=FALSE)

for (i in  seq(dim(annotated_merged_res_not_preg_preg_AI_miRNA)[1])){

  gene_id<-annotated_merged_res_not_preg_preg_AI_miRNA[i,1]
  gene_symbol<-annotated_merged_res_not_preg_preg_AI_miRNA$external_gene_name[i]
  if(gene_symbol==""){gene_symbol<-gene_id}

  cpm_AI<-cpm_AIpreg[rownames(cpm_AIpreg)==gene_id,]
  cpm_NP<-cpm_NOTpreg[rownames(cpm_NOTpreg)==gene_id,]

  data_chart_1<-rbind(data.frame(cpm=cpm_AI), data.frame(cpm=cpm_NP))
  data_chart_1$group<-rep(c("Preg_AI","Not_Preg"),c(6,5))
  data_chart_1$shape<-as.factor(c(0:10))
  data_chart_1$gene<-gene_symbol
  data_chart_1$chart<-i
  data_chart_1$fold_change<-annotated_merged_res_not_preg_preg_AI_miRNA[annotated_merged_res_not_preg_preg_AI_miR
NA$Row.names==gene_id,]$logFC
  data_chart_1$pvalue<-annotated_merged_res_not_preg_preg_AI_miRNA[annotated_merged_res_not_preg_preg_AI_miRNA$Ro
w.names==gene_id,]$PValue


  data_chart_2<-rbind(data_chart_2,data_chart_1)
}

pdf(file=paste(result_path,"04_30_plot_all_DEGs_preg_AI_not_preg_miRNA_before_LOOCV.pdf",sep="/"), width= 11, pap
er="USr", bg="transparent", onefile=TRUE)


for (i in seq(1,dim(annotated_merged_res_not_preg_preg_AI_miRNA)[1], 24)) {
  plots <- list()
  k<-1
  for (j in c(i:(i+23))){
    data_chart_3<-data_chart_2[data_chart_2$chart %in% j , ]
    plot<-ggplot(data=data_chart_3, aes(x=group , y=cpm, colour=group))+
      geom_jitter(position = position_jitter(width = .1, height=0), aes( shape=shape), size=2)+
      scale_shape_manual(values=c(0:11))+
      scale_x_discrete(data_chart_3$gene[1],labels=c("Preg_AI"="Preg_AI", "Not_Preg"="Not_Preg"))+
      scale_colour_manual(name=NULL, values = c("red", "blue"))+
      stat_summary(fun.y = median, fun.ymin = median, fun.ymax = median,colour = "gray", size = 0.2,  geom = "cro
ssbar", alpha=0.5)+
      scale_y_continuous("CPM")+
      ggtitle(paste("LogFC","=", round(data_chart_3$fold_change[1],1),"  ","P","=",round(data_chart_3$pvalue[1],2
), sep=c("")))+
      theme_bw()+
      theme(panel.grid= element_blank(),
            panel.background = element_blank(),
            panel.grid.minor = element_blank(),
            panel.grid.major = element_blank(),
            plot.background = element_blank(),
            strip.background = element_rect(fill = "white"),
            #strip.text.x = element_text(colour = 'black', face="italic",size = 9),
            #legend.text = element_text( colour = 'black', size = 10 ),
            axis.text.x = element_blank(),
            axis.text.y = element_text( colour = 'black', size = 8 ),
            axis.title = element_text( colour = 'black', size = 9 ),
            legend.key.size = unit(0.9,"cm"),
            legend.position="none",
            axis.ticks.x = element_blank(),
            plot.title = element_text(size = 7)
      )

    plots[[k]] <- plot
    k<-k+1
  }
  multiplot(plotlist = plots, cols = 6,layout = matrix(1:24, nrow=4, byrow = TRUE))
}
dev.off()
```

Leave-one-out cross validation

```
group<-factor(c("Preg_AI","Preg_AI","Preg_AI","Preg_AI","Preg_AI","Preg_AI","Preg_NB","Preg_NB","Preg_NB","Preg_N
B","Preg_NB","Preg_NB","Not_Preg","Not_Preg","Not_Preg","Not_Preg","Not_Preg"), levels=c("Preg_AI","Preg_NB", "No
t_Preg"))

out_list_IDs_LOOCV<-foreach(i = c(1:6,13:17), .inorder=FALSE,.packages=c("edgeR","DESeq2"), .verbose=FALSE ) %do%
{

group_a<-group[-i]
count_miRNA_plasma_a<-count_miRNA_plasma[,-i]

design<-model.matrix(~group_a)
dds<-DGEList(count=count_miRNA_plasma_a, group=group_a)
dds<-estimateDisp(dds, design, robust=TRUE)
dds<-glmFit(dds, design)
dds<-glmLRT(dds,coef="group_aNot_Preg")
res_not_preg_preg_AI_edgeR<-topTags(dds,n=Inf)$table

#DESeq
colData<-data.frame("group"=group_a)
rownames(colData)<-colnames(count_miRNA_plasma_a)
dds<-DESeqDataSetFromMatrix(countData=count_miRNA_plasma_a, colData=colData, design= ~group)
dds<-DESeq(dds)
res_not_preg_preg_AI_DeSeq<-results(dds, contrast=c("group","Not_Preg" ,"Preg_AI"), pAdjustMethod="fdr", tidy=TRU
E)


merged_res_not_preg_preg_AI_miRNA_a<-merge(res_not_preg_preg_AI_edgeR, res_not_preg_preg_AI_DeSeq, by.x="row.name
s", by.y="row")
merged_res_not_preg_preg_AI_miRNA_a<-merged_res_not_preg_preg_AI_miRNA_a[merged_res_not_preg_preg_AI_miRNA_a$PVal
ue<=0.03 & merged_res_not_preg_preg_AI_miRNA_a$pvalue<=0.03,]

rm(group_a,count_miRNA_plasma_a)

merged_res_not_preg_preg_AI_miRNA_a$Row.names
}

overlapping_genes_LOOCV<-Reduce(intersect, out_list_IDs_LOOCV)

merged_res_not_preg_preg_AI_miRNA<-merge(res_not_preg_preg_AI_miRNA_edgeR, res_not_preg_preg_AI_miRNA_DeSeq, by.x
="row.names", by.y="row")
merged_res_not_preg_preg_AI_miRNA_a<-merged_res_not_preg_preg_AI_miRNA[merged_res_not_preg_preg_AI_miRNA$Row.name
s %in% overlapping_genes_LOOCV,]
annotated_merged_res_not_preg_preg_AI_miRNA<-merge(merged_res_not_preg_preg_AI_miRNA_a,annotation.ensembl.symbol,
by.x="Row.names", by.y="ensembl_gene_id", all=FALSE)
annotated_merged_res_not_preg_preg_AI_miRNA<-annotated_merged_res_not_preg_preg_AI_miRNA[with(annotated_merged_re
s_not_preg_preg_AI_miRNA, order(logFC)), ]
dim(annotated_merged_res_not_preg_preg_AI_miRNA)#1
```

```
## [1]  1 17
```

```
#head(annotated_merged_res_not_preg_preg_AI_miRNA)
#write.table(annotated_merged_res_not_preg_preg_AI_miRNA, paste(result_path, "2020_01_04_annotated_merged_res_not
_preg_preg_AI_miRNA_after_LOOCV.txt"), quote=FALSE, sep="\t", row.names=FALSE)
rm(out_list_IDs_LOOCV)
```

```
knitr::kable(annotated_merged_res_not_preg_preg_AI_miRNA,format = 'pandoc')
```

Row.names	logFC	logCPM	LR	PValue	FDR	baseMean	log2FoldChange	lfcSE	stat	pvalue	padj
ENSBTAG00000052419	-5.096042	0.5841013	12.22747	0.0004709	0.068282	7.394997	-5.113215	1.951268	-2.620458	0.0087812	0.362537

Plot the charts for all genes inferred as differentially expressed between AI and non pregnant

```
cpm_AIpreg<-cpm_miRNA[,c(1,2,3,4,5,6)]
cpm_NOTpreg<-cpm_miRNA[,c(13,14,15,16,17)]
cpm_NBpreg<-cpm_miRNA[,c(7,8,9,10,11,12)]

data_chart_1<-data.frame( stringsAsFactors=FALSE)
data_chart_2<-data.frame( stringsAsFactors=FALSE)

for (i in  seq(dim(annotated_merged_res_not_preg_preg_AI_miRNA)[1])){

  gene_id<-annotated_merged_res_not_preg_preg_AI_miRNA[i,1]
  gene_symbol<-annotated_merged_res_not_preg_preg_AI_miRNA$external_gene_name[i]
  if(gene_symbol==""){gene_symbol<-gene_id}

  cpm_AI<-cpm_AIpreg[rownames(cpm_AIpreg)==gene_id,]
  cpm_NP<-cpm_NOTpreg[rownames(cpm_NOTpreg)==gene_id,]

  data_chart_1<-rbind(data.frame(cpm=cpm_AI), data.frame(cpm=cpm_NP))
  data_chart_1$group<-rep(c("Preg_AI","Not_Preg"),c(6,5))
  data_chart_1$shape<-as.factor(c(0:10))
  data_chart_1$gene<-gene_symbol
  data_chart_1$chart<-i
  data_chart_1$fold_change<-annotated_merged_res_not_preg_preg_AI_miRNA[annotated_merged_res_not_preg_preg_AI_miR
NA$Row.names==gene_id,]$logFC
  data_chart_1$pvalue<-annotated_merged_res_not_preg_preg_AI_miRNA[annotated_merged_res_not_preg_preg_AI_miRNA$Ro
w.names==gene_id,]$PValue


  data_chart_2<-rbind(data_chart_2,data_chart_1)
}

pdf(file=paste(result_path,"04_30_plot_all_DEGs_preg_AI_not_preg_miRNA_after_LOOCV.pdf",sep="/"), width= 11, pape
r="USr", bg="transparent", onefile=TRUE)


for (i in seq(1,dim(annotated_merged_res_not_preg_preg_AI_miRNA)[1], 24)) {
  plots <- list()
  k<-1
  for (j in c(i:(i+23))){
    data_chart_3<-data_chart_2[data_chart_2$chart %in% j , ]
    plot<-ggplot(data=data_chart_3, aes(x=group , y=cpm, colour=group))+
      geom_jitter(position = position_jitter(width = .1, height=0), aes( shape=shape), size=2)+
      scale_shape_manual(values=c(0:11))+
      scale_x_discrete(data_chart_3$gene[1],labels=c("Preg_AI"="Preg_AI", "Not_Preg"="Not_Preg"))+
      scale_colour_manual(name=NULL, values = c("red", "blue"))+
      stat_summary(fun.y = median, fun.ymin = median, fun.ymax = median,colour = "gray", size = 0.2,  geom = "cro
ssbar", alpha=0.5)+
      scale_y_continuous("CPM")+
      ggtitle(paste("LogFC","=", round(data_chart_3$fold_change[1],1),"  ","P","=",round(data_chart_3$pvalue[1],2
), sep=c("")))+
      theme_bw()+
      theme(panel.grid= element_blank(),
            panel.background = element_blank(),
            panel.grid.minor = element_blank(),
            panel.grid.major = element_blank(),
            plot.background = element_blank(),
            strip.background = element_rect(fill = "white"),
            #strip.text.x = element_text(colour = 'black', face="italic",size = 9),
            #legend.text = element_text( colour = 'black', size = 10 ),
            axis.text.x = element_blank(),
            axis.text.y = element_text( colour = 'black', size = 8 ),
            axis.title = element_text( colour = 'black', size = 9 ),
            legend.key.size = unit(0.9,"cm"),
            legend.position="none",
            axis.ticks.x = element_blank(),
            plot.title = element_text(size = 7)
      )

    plots[[k]] <- plot
    k<-k+1
  }
  multiplot(plotlist = plots, cols = 6,layout = matrix(1:24, nrow=4, byrow = TRUE))
}
dev.off()
```

AI Pregnant vs Pregnant to Natural Service

Results from edgeR

```
#colnames(count_miRNA_plasma)
group<-factor(c("Preg_AI","Preg_AI","Preg_AI","Preg_AI","Preg_AI","Preg_AI","Preg_NB","Preg_NB","Preg_NB","Preg_N
B","Preg_NB","Preg_NB","Not_Preg","Not_Preg","Not_Preg","Not_Preg","Not_Preg"), levels=c("Preg_AI","Preg_NB", "No
t_Preg"))
design<-model.matrix(~group)
dds<-DGEList(count=count_miRNA_plasma, group=group)
dds<-estimateDisp(dds, design, robust=TRUE)
dds<-glmFit(dds, design)
dds<-glmLRT(dds,coef="groupPreg_NB")
res_preg_AI_preg_NB_miRNA_edgeR<-topTags(dds,n=Inf)$table
#head(res_preg_AI_preg_NB_miRNA_edgeR,n=30)
#write.table(res_preg_AI_preg_NB_miRNA_edgeR, paste(result_path, "2019_04_30_res_preg_AI_preg_NB_miRNA_edgeR.tx
t"), quote=FALSE, sep="\t", row.names=FALSE)
```

Results from DESeq

```
design<-model.matrix(~group)
colData<-data.frame("group"=group)
rownames(colData)<-colnames(count_miRNA_plasma)
dds<-DESeqDataSetFromMatrix(countData=count_miRNA_plasma, colData=colData, design= ~group)
dds<-DESeq(dds)
res_preg_AI_preg_NB_miRNA_DeSeq<-results(dds, contrast=c("group","Preg_NB" ,"Preg_AI"), pAdjustMethod="fdr", tidy
=TRUE)
res_preg_AI_preg_NB_miRNA_DeSeq<-res_preg_AI_preg_NB_miRNA_DeSeq[with(res_preg_AI_preg_NB_miRNA_DeSeq, order(pval
ue)), ]
#head(res_preg_AI_preg_NB_miRNA_DeSeq,n=30)
#write.table(res_preg_AI_preg_NB_miRNA_DeSeq, paste(result_path, "2019_04_30_res_preg_AI_preg_NB_miRNA_DeSeq.tx
t"), quote=FALSE, sep="\t", row.names=FALSE)
```

Merge the results from edgeR and DESeq. (AI Pregnant vs Pregnant to Natural Service)

```
merged_res_preg_AI_preg_NB_miRNA<-merge(res_preg_AI_preg_NB_miRNA_edgeR, res_preg_AI_preg_NB_miRNA_DeSeq, by.x="r
ow.names", by.y="row")
merged_res_preg_AI_preg_NB_miRNA<-merged_res_preg_AI_preg_NB_miRNA[merged_res_preg_AI_preg_NB_miRNA$PValue<=0.03
 & merged_res_preg_AI_preg_NB_miRNA$pvalue<=0.03,]
dim(merged_res_preg_AI_preg_NB_miRNA) #0
```

```
## [1]  1 12
```

```
#head(merged_res_preg_AI_preg_NB_miRNA)
```

Pregnant to Natural Service vs non pregnant

Results from edgeR

```
#colnames(count_miRNA_plasma)
group<-factor(c("Preg_AI","Preg_AI","Preg_AI","Preg_AI","Preg_AI","Preg_AI","Preg_NB","Preg_NB","Preg_NB","Preg_N
B","Preg_NB","Preg_NB","Not_Preg","Not_Preg","Not_Preg","Not_Preg","Not_Preg"), levels=c("Preg_NB","Preg_AI","Not
_Preg"))
design<-model.matrix(~group)
dds<-DGEList(count=count_miRNA_plasma, group=group)
dds<-estimateDisp(dds, design, robust=TRUE)
dds<-glmFit(dds, design)
dds<-glmLRT(dds,coef="groupNot_Preg")
res_not_preg_preg_NB_miRNA_edgeR<-topTags(dds,n=Inf)$table
#head(res_not_preg_preg_NB_miRNA_edgeR,n=30)
#write.table(res_not_preg_preg_NB_miRNA_edgeR, paste(result_path, "2019_04_30_res_not_preg_preg_NB_miRNA_edgeR.tx
t"), quote=FALSE, sep="\t", row.names=FALSE)
```

Results from DESeq

```
design<-model.matrix(~group)
colData<-data.frame("group"=group)
rownames(colData)<-colnames(count_miRNA_plasma)
dds<-DESeqDataSetFromMatrix(countData=count_miRNA_plasma, colData=colData, design= ~group)
dds<-DESeq(dds)
res_not_preg_preg_NB_miRNA_DeSeq<-results(dds, contrast=c("group","Not_Preg" ,"Preg_NB"), pAdjustMethod="fdr", ti
dy=TRUE)
res_not_preg_preg_NB_miRNA_DeSeq<-res_not_preg_preg_NB_miRNA_DeSeq[with(res_not_preg_preg_NB_miRNA_DeSeq, order(p
value)), ]
#head(res_not_preg_preg_NB_miRNA_DeSeq,n=30)
#write.table(res_not_preg_preg_NB_miRNA_DeSeq, paste(result_path, "2019_04_30_res_not_preg_preg_NB_miRNA_DeSeq.tx
t"), quote=FALSE, sep="\t", row.names=FALSE)
```

Merge the results from edgeR and DESeq. (Pregnant to Natural Service vs non pregnant)

```
merged_res_not_preg_preg_NB_miRNA<-merge(res_not_preg_preg_NB_miRNA_edgeR, res_not_preg_preg_NB_miRNA_DeSeq, by.x
="row.names", by.y="row")
merged_res_not_preg_preg_NB_miRNA<-merged_res_not_preg_preg_NB_miRNA[merged_res_not_preg_preg_NB_miRNA$PValue<=0.
03 & merged_res_not_preg_preg_NB_miRNA$pvalue<=0.03,]
dim(merged_res_not_preg_preg_NB_miRNA) #1
```

```
## [1]  2 12
```

```
#head(merged_res_not_preg_preg_NB_miRNA)

annotated_merged_res_not_preg_preg_NB_miRNA<-merge(merged_res_not_preg_preg_NB_miRNA,annotation.ensembl.symbol, b
y.x="Row.names", by.y="ensembl_gene_id", all=FALSE)
#head(annotated_merged_res_not_preg_preg_NB_miRNA)
#write.table(annotated_merged_res_not_preg_preg_NB_miRNA, paste(result_path, "2019_04_30_annotated_merged_res_not
_preg_preg_NB_miRNA.txt"), quote=FALSE, sep="\t", row.names=FALSE)
```

Plot the charts for all genes inferred as differentially expressed between Pregnant to Natural Service and non pregnant

```
cpm_AIpreg<-cpm_miRNA[,c(1,2,3,4,5,6)]
cpm_NOTpreg<-cpm_miRNA[,c(13,14,15,16,17)]
cpm_NBpreg<-cpm_miRNA[,c(7,8,9,10,11,12)]

data_chart_1<-data.frame( stringsAsFactors=FALSE)
data_chart_2<-data.frame( stringsAsFactors=FALSE)

for (i in  seq(dim(annotated_merged_res_not_preg_preg_NB_miRNA)[1])){

  gene_id<-annotated_merged_res_not_preg_preg_NB_miRNA[i,1]
  gene_symbol<-annotated_merged_res_not_preg_preg_NB_miRNA$external_gene_name[i]
  if(gene_symbol==""){gene_symbol<-gene_id}

  cpm_NB<-cpm_NBpreg[rownames(cpm_NBpreg)==gene_id,]
  cpm_NP<-cpm_NOTpreg[rownames(cpm_NOTpreg)==gene_id,]

  data_chart_1<-rbind(data.frame(cpm=cpm_NB), data.frame(cpm=cpm_NP))
  data_chart_1$group<-rep(c("Preg_NB","Not_Preg"),c(6,5))
  data_chart_1$shape<-as.factor(c(0:10))
  data_chart_1$gene<-gene_symbol
  data_chart_1$chart<-i
  data_chart_1$fold_change<-annotated_merged_res_not_preg_preg_NB_miRNA[annotated_merged_res_not_preg_preg_NB_miR
NA$Row.names==gene_id,]$logFC
  data_chart_1$pvalue<-annotated_merged_res_not_preg_preg_NB_miRNA[annotated_merged_res_not_preg_preg_NB_miRNA$Ro
w.names==gene_id,]$PValue


  data_chart_2<-rbind(data_chart_2,data_chart_1)
}

pdf(file=paste(result_path,"04_30_plot_all_DEGs_preg_NB_not_preg_miRNA_before_LOOCV.pdf",sep="/"), width= 11, pap
er="USr", bg="transparent", onefile=TRUE)


for (i in seq(1,dim(annotated_merged_res_not_preg_preg_NB_miRNA)[1], 24)) {
  plots <- list()
  k<-1
  for (j in c(i:(i+23))){
    data_chart_3<-data_chart_2[data_chart_2$chart %in% j , ]
    plot<-ggplot(data=data_chart_3, aes(x=group , y=cpm, colour=group))+
      geom_jitter(position = position_jitter(width = .1, height=0), aes( shape=shape), size=2)+
      scale_shape_manual(values=c(0:11))+
      scale_x_discrete(data_chart_3$gene[1],labels=c("Preg_NB"="Preg_NB", "Not_Preg"="Not_Preg"))+
      scale_colour_manual(name=NULL, values = c("red", "black"))+
      stat_summary(fun.y = median, fun.ymin = median, fun.ymax = median,colour = "gray", size = 0.2,  geom = "cro
ssbar", alpha=0.5)+
      scale_y_continuous("CPM")+
      ggtitle(paste("LogFC","=", round(data_chart_3$fold_change[1],1),"  ","P","=",round(data_chart_3$pvalue[1],2
), sep=c("")))+
      theme_bw()+
      theme(panel.grid= element_blank(),
            panel.background = element_blank(),
            panel.grid.minor = element_blank(),
            panel.grid.major = element_blank(),
            plot.background = element_blank(),
            strip.background = element_rect(fill = "white"),
            #strip.text.x = element_text(colour = 'black', face="italic",size = 9),
            #legend.text = element_text( colour = 'black', size = 10 ),
            axis.text.x = element_blank(),
            axis.text.y = element_text( colour = 'black', size = 8 ),
            axis.title = element_text( colour = 'black', size = 9 ),
            legend.key.size = unit(0.9,"cm"),
            legend.position="none",
            axis.ticks.x = element_blank(),
            plot.title = element_text(size = 7)
      )

    plots[[k]] <- plot
    k<-k+1
  }
  multiplot(plotlist = plots, cols = 6,layout = matrix(1:24, nrow=4, byrow = TRUE))
}
dev.off()
```

Leave-one-out cross validation

```
group<-factor(c("Preg_AI","Preg_AI","Preg_AI","Preg_AI","Preg_AI","Preg_AI","Preg_NB","Preg_NB","Preg_NB","Preg_N
B","Preg_NB","Preg_NB","Not_Preg","Not_Preg","Not_Preg","Not_Preg","Not_Preg"), levels=c("Preg_NB", "Preg_AI","No
t_Preg"))

out_list_IDs_LOOCV<-foreach(i = c(7:17), .inorder=FALSE,.packages=c("edgeR","DESeq2"), .verbose=FALSE ) %do% {

group_a<-group[-i]
count_miRNA_plasma_a<-count_miRNA_plasma[,-i]

design<-model.matrix(~group_a)
dds<-DGEList(count=count_miRNA_plasma_a, group=group_a)
dds<-estimateDisp(dds, design, robust=TRUE)
dds<-glmFit(dds, design)
dds<-glmLRT(dds,coef="group_aNot_Preg")
res_not_preg_preg_NB_edgeR<-topTags(dds,n=Inf)$table

#DESeq
colData<-data.frame("group"=group_a)
rownames(colData)<-colnames(count_miRNA_plasma_a)
dds<-DESeqDataSetFromMatrix(countData=count_miRNA_plasma_a, colData=colData, design= ~group)
dds<-DESeq(dds)
res_not_preg_preg_NB_DeSeq<-results(dds, contrast=c("group","Not_Preg" ,"Preg_NB"), pAdjustMethod="fdr", tidy=TRU
E)


merged_res_not_preg_preg_NB_miRNA_a<-merge(res_not_preg_preg_NB_edgeR, res_not_preg_preg_NB_DeSeq, by.x="row.name
s", by.y="row")
merged_res_not_preg_preg_NB_miRNA_a<-merged_res_not_preg_preg_NB_miRNA_a[merged_res_not_preg_preg_NB_miRNA_a$PVal
ue<=0.03 & merged_res_not_preg_preg_NB_miRNA_a$pvalue<=0.03,]

rm(group_a,count_miRNA_plasma_a)

merged_res_not_preg_preg_NB_miRNA_a$Row.names
}

overlapping_genes_LOOCV<-Reduce(intersect, out_list_IDs_LOOCV)

merged_res_not_preg_preg_NB_miRNA<-merge(res_not_preg_preg_NB_miRNA_edgeR, res_not_preg_preg_NB_miRNA_DeSeq, by.x
="row.names", by.y="row")
merged_res_not_preg_preg_NB_miRNA_a<-merged_res_not_preg_preg_NB_miRNA[merged_res_not_preg_preg_NB_miRNA$Row.name
s %in% overlapping_genes_LOOCV,]
annotated_merged_res_not_preg_preg_NB_miRNA<-merge(merged_res_not_preg_preg_NB_miRNA_a,annotation.ensembl.symbol,
by.x="Row.names", by.y="ensembl_gene_id", all=FALSE)
annotated_merged_res_not_preg_preg_NB_miRNA<-annotated_merged_res_not_preg_preg_NB_miRNA[with(annotated_merged_re
s_not_preg_preg_NB_miRNA, order(logFC)), ]
dim(annotated_merged_res_not_preg_preg_NB_miRNA)#0
```

```
## [1]  0 17
```

```
#head(annotated_merged_res_not_preg_preg_NB_miRNA)
```

Assessment of transcript levels as predictors of pregnancy outcome

Supplementary Fig. 5

```
group<-factor(c("Preg_AI","Preg_AI","Preg_AI","Preg_AI","Preg_AI","Preg_AI",
                "Not_Preg","Not_Preg","Not_Preg","Not_Preg","Not_Preg",
                "Preg_AI","Not_Preg", "Not_Preg", "Not_Preg", "Preg_AI",
                "Preg_AI","Preg_AI", "Preg_AI", "Not_Preg", "Preg_AI",
                "Not_Preg", "Not_Preg"), levels=c("Preg_AI", "Not_Preg"))

class<-data.frame(group=group)

merged_datasets_a<-merged_datasets[,c(1:6,13:17,18:29)]

#variance stablize the data

design<-model.matrix(~group)
colData<-data.frame("group"=group)
rownames(colData)<-colnames(data)
dds<-DESeqDataSetFromMatrix(countData=merged_datasets_a, colData=colData, design= ~group)
dds<-DESeq(dds)
vsd <- varianceStabilizingTransformation(dds, fitType='local')
data_vsd <- assay(vsd)

data_vsd<-data_vsd[rownames(data_vsd) %in% merged_res_not_preg_preg_AI_mRNA_merged_a$Row.names,]

data.train <- data.frame(t(data_vsd[ ,c(12:23)]))
data.test  <- data.frame(t(data_vsd[ ,c(1:11)]))
classtr <-  as.factor(group[c(12:23)])
classts <-  as.factor(group[c(1:11)])

set.seed(1)
number <-4
repeats <- 5

control <- trainControl(method = "repeatedcv",
                        number = number ,
                        repeats = repeats,
                        classProbs = TRUE,
                        savePredictions = "final",
                        index = createResample(classtr, repeats*number),
                        #summaryFunction = twoClassSummary,
                        #returnResamp = "all",
                        allowParallel = TRUE)


my_models<-c( "ada","AdaBag","adaboost","avNNet","bagEarth","bagEarthGCV","bagFDAGCV","bayesglm","blackboost","Bs
tLm","bstSm",
 "bstTree","C5.0","C5.0Cost","C5.0Rules","C5.0Tree","cforest","CSimca","ctree","ctree2","deepboost","dnn",
 "dwdLinear","dwdRadial","earth","FRBCS.CHI","FRBCS.W","gamboost","gamLoess","gamSpline","gaussprLinear","gausspr
Poly","gaussprRadial",
 "gcvEarth","glm","glmboost","glmnet","glmStepAIC","hdda","hdrda","kknn","knn","lda","lda2",
 "LogitBoost","Mlda","mlp","mlpML","mlpWeightDecay","mlpWeightDecayML", "monmlp","msaenet","multinom","naive_baye
s","nb",
 "nnet","null","ordinalNet","ORFlog","ORFpls","ORFridge","ORFsvm","ownn","pam","parRF","partDSA",
 "pcaNNet","pda","pda2","plr","pls","PRIM","protoclass","ranger","rbfDDA","Rborist","regLogistic",
 "rf","RFlda","rfRules","rlda","rocc" )


#initial testing of the models not run here due to time constrain

cl <- makeCluster(32)
registerDoParallel(cl)
rand_integer<-sample.int(10000, 50, replace = FALSE)
data_runs<-data.frame()
data_runs<-foreach(model = my_models,.combine='rbind', .packages=c('caret'), .inorder=FALSE, .verbose=TRUE , .err
orhandling='remove') %:%
foreach(i = rand_integer, .combine='rbind', .packages=c('caret'), .inorder=FALSE, .verbose=TRUE , .errorhandling=
'remove') %dopar% {
  rm(model_train)
  set.seed(i)
  model_train<-train(data.train, classtr,  method = model, trControl=control)
  data.frame(model,t(caret::confusionMatrix(predict(model_train, data.test),classts)$overall), seed=i)
}
stopCluster(cl)

#write.table(data_runs, file = paste(result_path,"2020_01_25_prediction_metrics_all_models.txt",sep="/"), append
 = FALSE, quote = FALSE, sep = "\t",  eol = "\n", na = "NA", dec = ".", row.names = FALSE, col.names = TRUE)

#system("bzip2 -9 /mnt/storage/auburn/heifer_pregnancy/proj_2018/analysis/results/2020_01_25_prediction_metrics_a
ll_models.txt")
```

```
data_runs<-read.delim(paste(result_path,"2020_01_25_prediction_metrics_all_models.txt.bz2",sep="/"), stringsAsFac
tors=FALSE)

sumary_models<-aggregate( Accuracy ~ model, data_runs,  FUN=function(x) c(mn = mean(x), median = median(x), std=s
d(x), n=length(x)))
sumary_models<-sumary_models[order(-sumary_models$Accuracy[,1]),]
#head(sumary_models, 10)

sumary_models<-sumary_models[1:15,]
sumary_models_1<-sumary_models$Accuracy
sumary_models_1<-data.frame(sumary_models_1)
sumary_models_1$model<-factor(sumary_models$model, levels=rev(sumary_models$model))


ggplot(sumary_models_1,aes(y=mn, x=model)) +
geom_pointrange(aes(ymin = mn-std, ymax = mn+std),colour="black",size=0.1)+
geom_point(size=2 )+
scale_y_continuous(name="Accuracy")+
coord_flip()+
theme_minimal()+
theme(
text = element_text(size=12),
axis.title.x = element_text(color="black"),
axis.text.x  = element_text(color="black"),
axis.text.y  = element_text(color="black"),
axis.title.y  = element_blank()
)
```

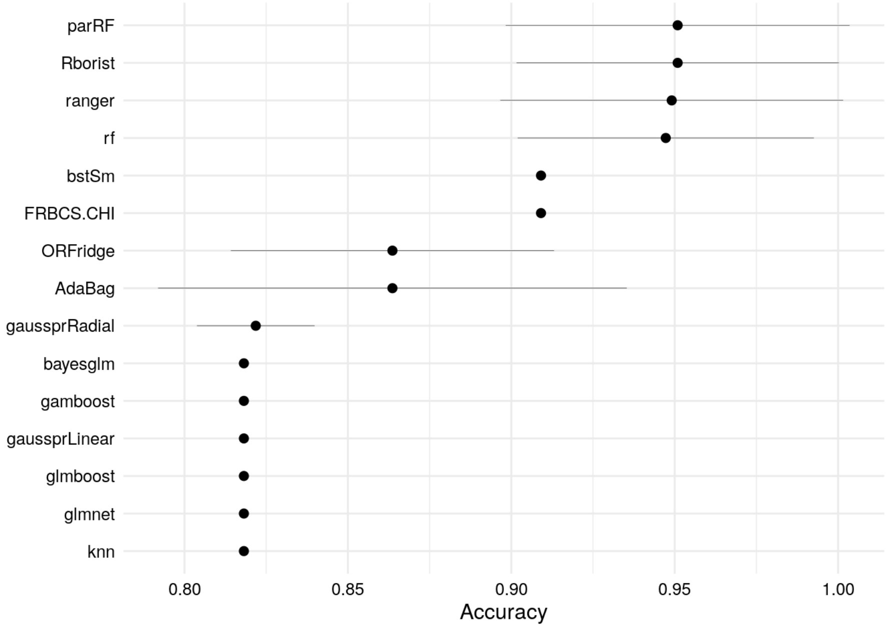

```
cl <- makeCluster(32)
registerDoParallel(cl)

rand_integer<-sample.int(10000, 2000, replace = FALSE)
data_runs<-data.frame()
data_runs<-foreach(i = rand_integer, .combine='rbind', .packages=c('caret'), .inorder=FALSE, .verbose=TRUE , .err
orhandling='remove') %dopar% {
  rm(model_train)
  set.seed(i)
  model_train<-train(data.train, classtr,  method = "parRF", trControl=control)
  data.frame(t(caret::confusionMatrix(predict(model_train, data.test),classts)$overall), seed=i)
}
stopCluster(cl)

attach(data_runs)
data_runs_b <- data_runs[order(-Accuracy),]
detach(data_runs)

write.table(data_runs_b, file= paste(result_path,"/2020_01_25_data_runs_b_2000_randomizations_parRF.txt", sep=""
), append = FALSE, quote = FALSE, sep = "\t" ,row.names = FALSE)
system("bzip2 -9 /mnt/storage/auburn/heifer_pregnancy/proj_2018/analysis/results/2020_01_25_data_runs_b_2000_rand
omizations_parRF.txt")


rand_integer<-sample.int(10000, 2000, replace = FALSE)
data_runs<-data.frame()
data_runs<-foreach(i = rand_integer, .combine='rbind', .packages=c('caret'), .inorder=FALSE, .verbose=TRUE , .err
orhandling='remove') %do% {
  rm(model_train)
  set.seed(i)
  model_train<-train(data.train, classtr,  method = "Rborist", trControl=control)
  data.frame(t(caret::confusionMatrix(predict(model_train, data.test),classts)$overall), seed=i)
}

attach(data_runs)
data_runs_b <- data_runs[order(-Accuracy),]
detach(data_runs)

write.table(data_runs_b, file= paste(result_path,"/2020_02_05_data_runs_b_2000_randomizations_Rborist.txt", sep=
""), append = FALSE, quote = FALSE, sep = "\t" ,row.names = FALSE)
system("bzip2 -9 /mnt/storage/auburn/heifer_pregnancy/proj_2018/analysis/results/2020_02_05_data_runs_b_2000_rand
omizations_Rborist.txt")


cl <- makeCluster(32)
registerDoParallel(cl)

rand_integer<-sample.int(10000, 2000, replace = FALSE)
data_runs<-data.frame()
data_runs<-foreach(i = rand_integer, .combine='rbind', .packages=c('caret'), .inorder=FALSE, .verbose=TRUE , .err
orhandling='remove') %dopar% {
  rm(model_train)
  set.seed(i)
  model_train<-train(data.train, classtr,  method = "ranger", trControl=control)
  data.frame(t(caret::confusionMatrix(predict(model_train, data.test),classts)$overall), seed=i)
}
stopCluster(cl)

attach(data_runs)
data_runs_b <- data_runs[order(-Accuracy),]
detach(data_runs)

write.table(data_runs_b, file= paste(result_path,"/2020_02_05_data_runs_b_2000_randomizations_ranger.txt", sep=""
), append = FALSE, quote = FALSE, sep = "\t" ,row.names = FALSE)
system("bzip2 -9 /mnt/storage/auburn/heifer_pregnancy/proj_2018/analysis/results/2020_02_05_data_runs_b_2000_rand
omizations_ranger.txt")


cl <- makeCluster(32)
registerDoParallel(cl)

rand_integer<-sample.int(10000, 2000, replace = FALSE)
data_runs<-data.frame()
data_runs<-foreach(i = rand_integer, .combine='rbind', .packages=c('caret'), .inorder=FALSE, .verbose=TRUE , .err
orhandling='remove') %dopar% {
  rm(model_train)
  set.seed(i)
  model_train<-train(data.train, classtr,  method = "rf", trControl=control)
  data.frame(t(caret::confusionMatrix(predict(model_train, data.test),classts)$overall), seed=i)
}
stopCluster(cl)
```

```
attach(data_runs)
data_runs_b <- data_runs[order(-Accuracy),]
detach(data_runs)

write.table(data_runs_b, file= paste(result_path,"/2020_02_05_data_runs_b_2000_randomizations_rf.txt", sep=""), a
ppend = FALSE, quote = FALSE, sep = "\t" ,row.names = FALSE)
system("bzip2 -9 /mnt/storage/auburn/heifer_pregnancy/proj_2018/analysis/results/2020_02_05_data_runs_b_2000_rand
omizations_rf.txt")



cl <- makeCluster(32)
registerDoParallel(cl)

rand_integer<-sample.int(10000, 2000, replace = FALSE)
data_runs<-data.frame()
data_runs<-foreach(i = rand_integer, .combine='rbind', .packages=c('caret'), .inorder=FALSE, .verbose=TRUE , .err
orhandling='remove') %dopar% {
  rm(model_train)
  set.seed(i)
  model_train<-train(data.train, classtr,  method = "bstSm", trControl=control)
  data.frame(t(caret::confusionMatrix(predict(model_train, data.test),classts)$overall), seed=i)
}
stopCluster(cl)

attach(data_runs)
data_runs_b <- data_runs[order(-Accuracy),]
detach(data_runs)

write.table(data_runs_b, file= paste(result_path,"/2020_01_25_data_runs_b_2000_randomizations_bstSm.txt", sep=""
), append = FALSE, quote = FALSE, sep = "\t" ,row.names = FALSE)
system("bzip2 -9 /mnt/storage/auburn/heifer_pregnancy/proj_2018/analysis/results/2020_01_25_data_runs_b_2000_rand
omizations_bstSm.txt")



cl <- makeCluster(32)
registerDoParallel(cl)

rand_integer<-sample.int(10000, 2000, replace = FALSE)
data_runs<-data.frame()
data_runs<-foreach(i = rand_integer, .combine='rbind', .packages=c('caret'), .inorder=FALSE, .verbose=TRUE , .err
orhandling='remove') %dopar% {
  rm(model_train)
  set.seed(i)
  model_train<-train(data.train, classtr,  method = "FRBCS.CHI", trControl=control)
  data.frame(t(caret::confusionMatrix(predict(model_train, data.test),classts)$overall), seed=i)
}
stopCluster(cl)

attach(data_runs)
data_runs_b <- data_runs[order(-Accuracy),]
detach(data_runs)

write.table(data_runs_b, file= paste(result_path,"/2020_01_25_data_runs_b_2000_randomizations_FRBCS.CHI.txt", sep
=""), append = FALSE, quote = FALSE, sep = "\t" ,row.names = FALSE)
system("bzip2 -9 /mnt/storage/auburn/heifer_pregnancy/proj_2018/analysis/results/2020_01_25_data_runs_b_2000_rand
omizations_FRBCS.CHI.txt")
```

Figure 5d

```
acuracy_parRF<-read.delim("/mnt/storage/auburn/heifer_pregnancy/proj_2018/analysis/results/2020_01_25_data_runs_b
_2000_randomizations_parRF.txt.bz2")
table(acuracy_parRF$Accuracy)
```

```
## 
## 0.818181818181818 0.909090909090909                 1 
##                11              1063               926
```

```
acuracy_parRF$Accuracy<-round(acuracy_parRF$Accuracy,2)

acuracy_parRF_point<-round(unique(acuracy_parRF[,-c(2,8)]),2)

plot_point<-ggplot(acuracy_parRF_point, aes(y=Accuracy, x=as.factor(Accuracy)))+
geom_errorbar(aes(ymin = AccuracyLower, ymax = AccuracyUpper), width = 0.1)+
geom_point(size=2, color="red")+
scale_y_continuous(name=paste("Accuracy ", " CI", sep = "\u00B1"))+
theme_minimal()+
theme(
text = element_text(size=11),
axis.title.x = element_blank(),
axis.text.x  = element_blank(),
axis.text.y  = element_text(color="black"),
axis.title.y  = element_text(color="black"),
plot.margin = unit(c(0,0,0,0.3), "cm")
)

plot_histogram<-ggplot(acuracy_parRF, aes(as.factor(Accuracy)))+
geom_bar(aes(y = (..count..)/sum(..count..)),width=0.4)+
scale_x_discrete(name="Accuracy")+
scale_y_continuous(labels =percent_format(accuracy = 2) )+
coord_flip()+
theme_minimal()+
theme(
text = element_text(size=11),
axis.text.y  = element_text(color="black"),
axis.text.x  = element_text(color="black", angle=90),
axis.title.x  = element_blank(),
plot.margin = unit(c(0,0,0,0), "cm")
)

#png(filename=paste(result_path,"2020_02_10_accuracy_prediction_parRF.png",sep="/"), width=750, height=450, res=3
00, bg="transparent")
#grid.arrange( plot_histogram,plot_point, nrow = 1)
#dev.off()

grid.arrange( plot_histogram,plot_point, nrow = 1)
```

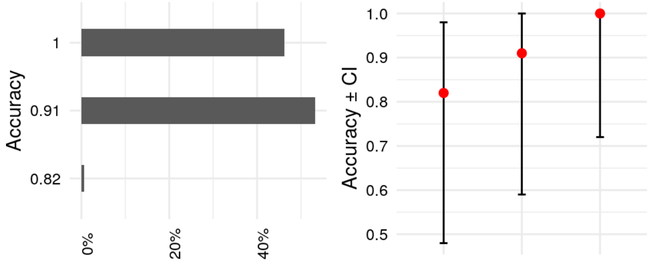

Figure 5e

```
acuracy_parRF<-read.delim("/mnt/storage/auburn/heifer_pregnancy/proj_2018/analysis/results/2020_01_25_data_runs_b
_2000_randomizations_parRF.txt.bz2")
table(acuracy_parRF$Accuracy)
acuracy_parRF$Accuracy<-round(acuracy_parRF$Accuracy,2)

acuracy_parRF_point<-round(unique(acuracy_parRF[,-c(2,8)]),2)

acuracy_parRF_09<-acuracy_parRF[acuracy_parRF$Accuracy>=0.9 ,]

cl <- makeCluster(32)
registerDoParallel(cl)
variable_importance_09<-data.frame()
variable_importance_09<-foreach(i = acuracy_parRF_09$seed, .combine='cbind', .packages=c('caret'), .inorder=FALSE
, .verbose=TRUE , .errorhandling='remove') %dopar% {
  rm(model_train)
  set.seed(i)
  model_train<-train(data.train, classtr,  method = "parRF", trControl=control)
  importance<-varImp(model_train, scale=F)$importance
  importance$gene<-(rownames(importance))
  importance<-importance[with(importance, order(gene)),]$Overall
}
stopCluster(cl)

rownames(variable_importance_09)<-colnames(data.train)

write.table(variable_importance_09, file= paste(result_path,"/2020_03_11_variable_importance_parRF.txt", sep=""),
append = FALSE, quote = FALSE, sep = "\t" ,row.names = TRUE)
system("bzip2 -9 /mnt/storage/auburn/heifer_pregnancy/proj_2018/analysis/results/2020_03_11_variable_importance_p
arRF.txt")
```

```
variable_importance_09<-as.matrix(read.delim(paste(result_path,"/2020_03_11_variable_importance_parRF.txt.bz2", s
ep=""),row.names=1))
variable_importance_09<-cbind(variable_importance_09,average=rowMeans2(variable_importance_09), std=rowVars(varia
ble_importance_09,std = TRUE))

variable_importance_09<-variable_importance_09[order(-variable_importance_09[,1990]),]

variable_importance_09_plot<-as.data.frame(variable_importance_09[1:5,1990:1991 ])
variable_importance_09_plot$genes<-factor(rownames(variable_importance_09_plot),levels=rev(c("ENSBTAG00000049161"
,"ENSBTAG00000051249","ENSBTAG00000002615","ENSBTAG00000017243","ENSBTAG00000001412")))

plot_point<-ggplot(variable_importance_09_plot, aes(y=average, x=genes))+
geom_pointrange(aes(ymin = average-std, ymax = average+std),colour="blue",size=0.1)+
geom_point(size=0.4, colour="blue" )+
scale_y_continuous(name="Importance")+
scale_x_discrete(labels=c("N6AMT1", "GATA3", "LONRF3", "SMIM26", "RPL39"))+
coord_flip()+
theme_minimal()+
theme(
text = element_text(size=12),
axis.title.x = element_text(color="black"),
axis.text.x  = element_text(color="black"),
axis.text.y  = element_text(color="black", face="italic"),
axis.title.y  = element_blank()
)

#png(filename=paste(result_path,"2020_02_10_gene_importance.png",sep="/"), width=600, height=450, res=310, bg="tr
ansparent")
#plot_point
#dev.off()
plot_point
```

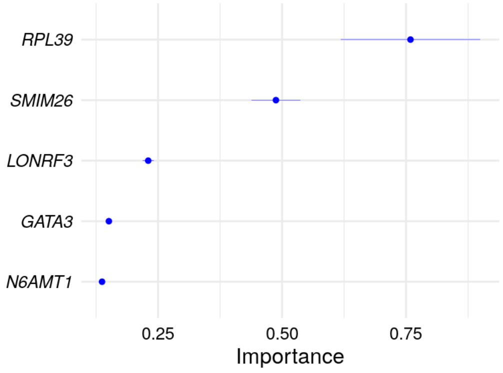

Figure 5f

```
variable_importance_09_plot$gene_symbols<-c("RPL39","SMIM26" ,"LONRF3", "GATA3" , "N6AMT1")

cpm_AIpreg<-cpm_merged_datasets[,c(1,2,3,4,5,6,18,22,23,24,25,27)]
cpm_NOTpreg<-cpm_merged_datasets[,c(13,14,15,16,17,19,20,21,26,28,29)]


data_chart_1<-data.frame( stringsAsFactors=FALSE)
data_chart_2<-data.frame( stringsAsFactors=FALSE)

for (i in  seq(dim(variable_importance_09_plot)[1])){

  gene_id<-variable_importance_09_plot$genes[i]
  gene_symbol<-variable_importance_09_plot$gene_symbols[i]
  if(gene_symbol==""){gene_symbol<-gene_id}

  cpm_AI<-cpm_AIpreg[rownames(cpm_AIpreg)==gene_id,]
  cpm_NP<-cpm_NOTpreg[rownames(cpm_NOTpreg)==gene_id,]

  data_chart_1<-rbind(data.frame(cpm=cpm_AI), data.frame(cpm=cpm_NP))
  data_chart_1$group<-as.factor(rep(c("Preg_AI","Not_Preg"),c(12,11)))
  data_chart_1$shape<-as.factor(rep(c(2,1,2,1),c(6,6,5,6)) )
  data_chart_1$gene<-gene_symbol
  data_chart_1$chart<-i
  data_chart_1$fold_change<-DEG_AI_NP_mRNA_merged[DEG_AI_NP_mRNA_merged$Row.names==gene_id,]$logFC
  data_chart_1$pvalue<-DEG_AI_NP_mRNA_merged[DEG_AI_NP_mRNA_merged$Row.names==gene_id,]$PValue

  data_chart_2<-rbind(data_chart_2,data_chart_1)
}

data_chart_2$gene<-factor(data_chart_2$gene,levels=c("RPL39","SMIM26" ,"LONRF3", "GATA3" , "N6AMT1"))


plot<-ggplot(data=data_chart_2, aes(x=group , y=cpm, colour=group, shape=shape) )+
      geom_jitter(position = position_jitterdodge(dodge.width = 1), size=2)+
      scale_shape_manual(values=c(15,16), labels=c("year 1", "year 2"))+
      scale_colour_manual(name=NULL, values = c("#D55E00", "#0072B2"),labels=c("non preg", "AI preg"))+
      #stat_summary(fun.y = median, fun.ymin = median, fun.ymax = median,colour = "gray", size = 0.2,  geom = "cr
ossbar", alpha=0.5)+
      scale_y_continuous("CPM")+
      facet_wrap(~gene, scales="free", nrow=1)+
      theme_bw()+
      theme(panel.grid= element_blank(),
            panel.background = element_blank(),
            panel.grid.minor = element_blank(),
            panel.grid.major = element_blank(),
            plot.background = element_blank(),
            strip.background = element_rect(fill = "white"),
            strip.text.x = element_text(colour = 'black', face="italic",size = 10),
            legend.text = element_text( colour = 'black', size = 10 ),
            axis.text.x = element_blank(),
            axis.text.y = element_text( colour = 'black', size = 10 ),
            axis.title.y = element_text( colour = 'black', size = 10 ),
            axis.title.x = element_blank(),
            #legend.key.size = unit(0.9,"cm"),
            legend.position="bottom",
            legend.margin=margin(0,0,0,0),
            legend.box.margin=margin(-10,-10,-10,-10),
            legend.title = element_blank(),
            axis.ticks.x = element_blank()
      )

#png(file=paste(result_path,"2020_02_10_plot_DEGs_predictors_random_forest.png",sep="/"), width= 1100, height= 49
0,  bg="transparent", res=300)
#plot
#dev.off()
plot
```

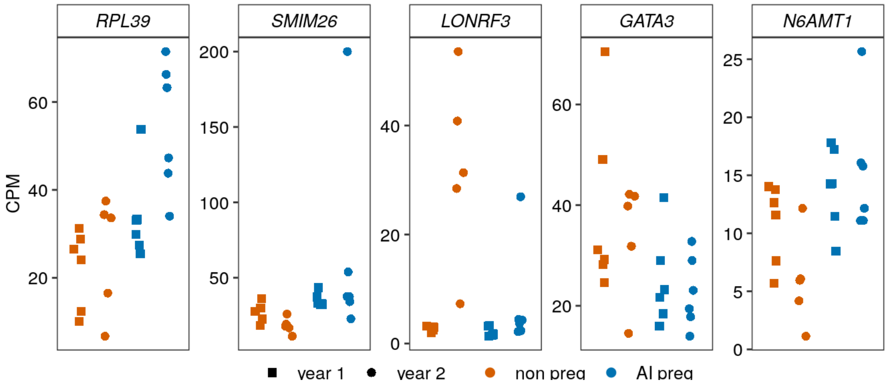


```
sessionInfo()
```

```
## R version 4.0.1 (2020-06-06)
## Platform: x86_64-pc-linux-gnu (64-bit)
## Running under: Ubuntu 18.04.4 LTS
## 
## Matrix products: default
## BLAS:   /usr/lib/x86_64-linux-gnu/blas/libblas.so.3.7.1
## LAPACK: /usr/lib/x86_64-linux-gnu/lapack/liblapack.so.3.7.1
## 
## locale:
##  [1] LC_CTYPE=en_US.UTF-8       LC_NUMERIC=C              
##  [3] LC_TIME=en_US.UTF-8        LC_COLLATE=en_US.UTF-8    
##  [5] LC_MONETARY=en_US.UTF-8    LC_MESSAGES=en_US.UTF-8   
##  [7] LC_PAPER=en_US.UTF-8       LC_NAME=C                 
##  [9] LC_ADDRESS=C               LC_TELEPHONE=C            
## [11] LC_MEASUREMENT=en_US.UTF-8 LC_IDENTIFICATION=C       
## 
## attached base packages:
##  [1] grid      parallel  stats4    stats     graphics  grDevices utils    
##  [8] datasets  methods   base     
## 
## other attached packages:
##  [1] networkD3_0.4               stringi_1.4.6              
##  [3] scales_1.1.1                TestCor_0.0.1.1            
##  [5] caretEnsemble_2.0.1         caret_6.0-86               
##  [7] lattice_0.20-41             circlize_0.4.10            
##  [9] ComplexHeatmap_2.4.2        parallelDist_0.2.4         
## [11] flashClust_1.01-2           gridExtra_2.3              
## [13] ggpubr_0.3.0                Rtsne_0.15                 
## [15] DT_0.13                     org.Bt.eg.db_3.11.4        
## [17] AnnotationDbi_1.50.0        gtools_3.8.2               
## [19] doParallel_1.0.15           iterators_1.0.12           
## [21] foreach_1.5.0               bigmemory_4.5.36           
## [23] reshape2_1.4.4              WGCNA_1.69                 
## [25] fastcluster_1.1.25          dynamicTreeCut_1.63-1      
## [27] goseq_1.40.0                geneLenDataBase_1.24.0     
## [29] BiasedUrn_1.07              biomaRt_2.44.1             
## [31] ggrepel_0.8.2               ggplot2_3.3.2              
## [33] statmod_1.4.34              edgeR_3.30.3               
## [35] limma_3.44.3                DESeq2_1.28.1              
## [37] SummarizedExperiment_1.18.1 DelayedArray_0.14.0        
## [39] matrixStats_0.56.0          Biobase_2.48.0             
## [41] GenomicRanges_1.40.0        GenomeInfoDb_1.24.2        
## [43] IRanges_2.22.2              S4Vectors_0.26.1           
## [45] BiocGenerics_0.34.0        
## 
## loaded via a namespace (and not attached):
##   [1] tidyselect_1.1.0         RSQLite_2.2.0            htmlwidgets_1.5.1       
##   [4] BiocParallel_1.22.0      pROC_1.16.2              munsell_0.5.0           
##   [7] codetools_0.2-16         preprocessCore_1.50.0    withr_2.2.0             
##  [10] colorspace_1.4-1         highr_0.8                knitr_1.28              
##  [13] rstudioapi_0.11          ggsignif_0.6.0           labeling_0.3            
##  [16] GenomeInfoDbData_1.2.3   farver_2.0.3             bit64_0.9-7             
##  [19] vctrs_0.3.1              generics_0.0.2           ipred_0.9-9             
##  [22] xfun_0.15                BiocFileCache_1.12.0     R6_2.4.1                
##  [25] markdown_1.1             clue_0.3-57              locfit_1.5-9.4          
##  [28] bitops_1.0-6             assertthat_0.2.1         nnet_7.3-14             
##  [31] gtable_0.3.0             timeDate_3043.102        rlang_0.4.6             
##  [34] genefilter_1.70.0        GlobalOptions_0.1.2      splines_4.0.1           
##  [37] rtracklayer_1.48.0       rstatix_0.6.0            ModelMetrics_1.2.2.2    
##  [40] acepack_1.4.1            impute_1.62.0            broom_0.5.6             
##  [43] checkmate_2.0.0          yaml_2.2.1               abind_1.4-5             
##  [46] GenomicFeatures_1.40.0   backports_1.1.8          Hmisc_4.4-0             
##  [49] tools_4.0.1              lava_1.6.7               ellipsis_0.3.1          
##  [52] RColorBrewer_1.1-2       Rcpp_1.0.4.6             plyr_1.8.6              
##  [55] base64enc_0.1-3          progress_1.2.2           zlibbioc_1.34.0         
##  [58] purrr_0.3.4              RCurl_1.98-1.2           prettyunits_1.1.1       
##  [61] rpart_4.1-15             openssl_1.4.1            pbapply_1.4-2           
##  [64] GetoptLong_1.0.0         cowplot_1.0.0            haven_2.3.1             
##  [67] cluster_2.1.0            magrittr_1.5             data.table_1.12.8       
##  [70] openxlsx_4.1.5           hms_0.5.3                mime_0.9                
##  [73] evaluate_0.14            xtable_1.8-4             XML_3.99-0.3            
##  [76] rio_0.5.16               jpeg_0.1-8.1             readxl_1.3.1            
##  [79] shape_1.4.4              compiler_4.0.1           tibble_3.0.1            
##  [82] crayon_1.3.4             htmltools_0.5.0          mgcv_1.8-31             
##  [85] Formula_1.2-3            tidyr_1.1.0              geneplotter_1.66.0      
##  [88] RcppParallel_5.0.1       lubridate_1.7.9          DBI_1.1.0               
##  [91] dbplyr_1.4.4             MASS_7.3-51.6            rappdirs_0.3.1          
##  [94] Matrix_1.2-18            car_3.0-8                igraph_1.2.5            
##  [97] gower_0.2.1              forcats_0.5.0            pkgconfig_2.0.3         
## [100] bigmemory.sri_0.1.3      GenomicAlignments_1.24.0 foreign_0.8-80          
## [103] recipes_0.1.12           annotate_1.66.0          XVector_0.28.0          
```

```
## [106] prodlim_2019.11.13       stringr_1.4.0            digest_0.6.25
## [109] Biostrings_2.56.0        rmarkdown_2.3            cellranger_1.1.0
## [112] htmlTable_2.0.0          curl_4.3                 Rsamtools_2.4.0
## [115] rjson_0.2.20             jsonlite_1.6.1           lifecycle_0.2.0
## [118] nlme_3.1-148             carData_3.0-4            askpass_1.1
## [121] pillar_1.4.4             httr_1.4.1               survival_3.2-3
## [124] GO.db_3.11.4             glue_1.4.1               zip_2.0.4
## [127] png_0.1-7                bit_1.1-15.2             class_7.3-17
## [130] blob_1.2.1               latticeExtra_0.6-29      memoise_1.1.0
## [133] dplyr_1.0.0
```
